# Trajectory and genetic correlation mapping of cross-disease risks following incident type 2 inflammatory diseases

**DOI:** 10.1038/s43856-026-02000-y

**Published:** 2026-11-06

**Authors:** Yask Gupta, Henning Olbrich, Diamant Thaci, Katja Bieber, Philip Curman, Ralf J. Ludwig

**Affiliations:** 1 Institute and Comprehensive Center for Inflammation Medicine, University of Lübeck, Lübeck, Germany; 2 Department of Dermatology, University-Hospital Schleswig-Holstein (UKSH), Lübeck, Germany; 3 Lübeck Institute of Experimental Dermatology, University of Lübeck, Lübeck, Germany; 4 Dermato-Venereology Clinic, Karolinska University Hospital, Stockholm, Sweden; 5 Department of Medical Epidemiology and Biostatistics, Karolinska Institutet, Stockholm, Sweden; 6 Dermatology and Venereology Division, Department of Medicine (Solna), Karolinska Institutet, Stockholm, Sweden

**Keywords:** Epidemiology, Allergy

## Abstract

**Background:**

Type 2 inflammatory diseases (T2IDs) such as atopic dermatitis (AD), asthma, food allergy, eosinophilic esophagitis (EoE), chronic sinusitis (CS), and others, are immune-mediated conditions that frequently co-occur across organ systems and contribute to substantial disease burden. While the atopic march has long served as a conceptual model for T2ID progression, it remains unclear whether this linear sequence captures the broader comorbidity structure of T2IDs.

**Methods:**

We systematically analyzed comorbidity patterns across nine T2IDs using two complementary methods: Large-scale real-world data (RWD) from over 129 million electronic health records, genome-wide and local genetic correlation, including three novel (asthma, chronic sinusitis, allergic rhinitis) metanalyses of genome-wide association studies.

**Results:**

Retrospective matched cohort analyses reveal a highly interconnected disease network in which CS emerged as a central node, increasing the risk of multiple other T2IDs across diverse patient populations. AD, food allergy, and vasomotor and allergic rhinitis also show strong cross-disease effects. Subgroup analyses uncover notable age-, sex-, and race-specific disparities. Genetic correlation analyses support shared heritability among many disease pairs, complemented by the temporal ordering of associations present in the RWD. Local genetic correlation position asthma as the central node of the shared polygenic architecture, converging on canonical type 2 inflammatory loci.

**Conclusions:**

Together, our results highlight that T2ID progression extends beyond the atopic march and is shaped by a complex and heterogeneous network of interactions. This work provides a data-driven framework to guide risk stratification, inform early preventive strategies, and prioritize upstream disease targets within the type 2 inflammatory spectrum.

## Plain language Summary

Some long-lasting diseases, for example such as eczema, asthma, food allergy and ongoing sinus inflammation, are driven by the same kind of overactive immune response. People who develop one often go on to develop others, but the order in which they appear is not well understood. We studied health records from more than 129 million people to see which conditions tended to follow which. We also compared the genetic make-up behind each condition. We found that these diseases form a connected network rather than a fixed sequence. Ongoing sinus inflammation often appeared first and was followed by several others. Risks also differed by age, sex and ethnic background. Knowing which condition may come next could help doctors identify people at risk earlier.

## Introduction

Type 2 inflammatory diseases (T2IDs) represent a group of chronic, immune-mediated diseases affecting the skin, airways and gastrointestinal tract. Despite their phenotypic diversity, T2IDs such as atopic dermatitis (AD), asthma, prurigo nodularis (PN), food allergy, eosinophilic esophagitis (EoE), chronic sinusitis (CS), vasomotor and allergic rhinitis (VMR/AR), chronic obstructive pulmonary disease (COPD) and bullous pemphigoid (BP) are united by overlapping inflammatory signatures, including the involvement of type 2 cytokines (interleukin [IL]-4, IL-5, IL-13), eosinophils, and IgE-driven pathways^1,2^.

T2IDs are highly prevalent and exert a substantial burden on patients and healthcare systems. Given the high degree of comorbidity among T2IDs, the cumulative prevalence of having at least one of these diseases in the general population ranges between 30 and 50%^3,4^. Although precise estimates of disability-adjusted life years (DALYs) and direct annual healthcare costs are not available for each of the mentioned T2IDs individually, available data suggest that DALYs range from 123 per 100,000 population (AD) to 926 per 100,000 population in COPD. In the United States alone, direct annual healthcare costs attributable to T2IDs are estimated between $5 billion and $31 billion, underscoring the substantial socioeconomic impact of these conditions ^5–9^.

Beyond their individual burden, T2IDs frequently co-occur, often across organ systems^10^. A well-known example for this comorbidity pattern is the atopic march, in which early-onset atopic dermatitis is frequently followed by food allergy, allergic rhinitis (AR) and asthma, highlighting a temporal and mechanistic progression across barrier organs^11,12^. The concept is supported by longitudinal birth cohort studies such as the German Multicenter Allergy Study (MAS), which demonstrates sequential disease onset and provide mechanistic insights into skin barrier dysfunction, allergen sensitization, and Th2-driven inflammation^13^. However, recent evidence has challenged the notion of a universal progression, i.e. the MAS noted that not all children with early-onset AD will subsequently develop asthma^14^. Thus, not all patients follow the proposed trajectory in the atopic march, and alternative comorbidity patterns have emerged from population-based studies using data-driven clustering or latent class analysis. These findings suggest that the atopic march represents one of several possible trajectories within a broader and more heterogeneous network of T2IDs^15,16^. However, this broader structure of T2ID comorbidity patterns across the full spectrum of T2IDs, and the extent to which these reflect shared genetic risk, remains incompletely understood. Furthermore, previous studies largely focused on single T2IDs, specific age groups, or limited outcome measures. In rare cases, disparities in sex or race were addressed.

In addition to clinical and epidemiologic evidence, genetic data are beginning to emerge that support shared susceptibility across T2IDs. Genome-wide genetic correlation analyses demonstrated heritability overlap for a subset of T2ID pairs, most notably between AD and asthma^17^, or asthma and AR^18,19^, where robust genetic correlations have been consistently reported. In some instances, Mendelian randomization (MR) studies demonstrated directional, potentially causal relationships, such as from AD to asthma^20^. However, for the majority of T2ID combinations, especially those encompassing diseases such as EoE, PN or BP, genetic correlation or data from MR studies remain limited or are entirely lacking. Overall, while initial evidence underscores the genetic interconnectedness of certain T2IDs, a comprehensive understanding of the genetic architecture underlying comorbidity across the full spectrum of T2IDs is still incomplete.

Taken together, several key knowledge gaps relating to T2ID trajectories remain: first, previous studies rarely examined comorbidity patterns across the full spectrum of T2IDs in a unified, systematic framework. Second, we are not aware of investigations have combined large-scale real-world data (RWD) with genetic correlation analyses to explore whether observed clinical associations reflect shared heritable risk. Third, although individual T2IDs have been associated with age-, sex- and racial disparities, it is unclear how these influence comorbidity trajectories across diseases. Addressing these gaps is essential for improving risk stratification and to guide the development of treatment decisions. To address these knowledge gaps, we conducted a comprehensive analysis of nine major T2IDs using retrospective cohort analyses from the TriNetX electronic health record (EHR) network, complemented by genome-wide genetic correlation estimates. This dual approach allowed us to (i) map cross-disease risks in a large, diverse populations, (ii) assess the degree of genetic overlap across T2IDs, (iii) integrate symmetric genome-wide genetic correlations with the temporal ordering available from retrospective cohort analyses, such that disease pairs with both shared heritability and a consistent clinical temporal sequence can be interpreted with greater confidence than either methodology alone, and (iv) compare cross-disease risks among T2IDs based on two independent methodologies, overall strengthening the results of our findings. From a clinical perspective, such insights could shape screening strategies for at-risk populations, support early intervention in disease trajectories, and help identify disease clusters that may benefit from shared therapeutic approaches.

Here, we show that T2IDs form an interconnected comorbidity network that extends beyond the atopic march, with chronic sinusitis occupying a central position in the clinical trajectory network and asthma at the centre of the shared polygenic architecture. Cross-disease risks differ markedly by age, sex and self-reported ancestry, and the shared genetic signal converges on canonical type 2 inflammatory loci. Together, these findings provide a framework for risk stratification and early preventive strategies across the type 2 inflammatory spectrum.

## Methods

### Retrospective cohort studies

#### Ethics

This study used data from the TriNetX US Collaborative Network and publicly available or summary-level genetic datasets from the GWAS Catalog, GBMI, GERA, UK Biobank-derived studies and FinnGen. No individual-level or controlled-access UK Biobank data were accessed directly for this study. UK Biobank-derived GWAS summary statistics were obtained from publicly available datasets deposited in the GWAS Catalog; therefore, no UK Biobank project/application number was required for the present analyses. FinnGen GWAS summary statistics were accessed through the FinnGen data portal following user registration. Access to these summary-level data does not involve a project-specific application or application ID. GWAS summary statistics obtained through the GWAS Catalog, GBMI and GERA were used in accordance with the access conditions and terms of use specified by the respective data providers and source studies. Where applicable, ethical approval and informed consent for the original collection and use of participant data were obtained by the corresponding contributing studies, cohorts or biobanks, as reported in the original publications and data repositories. Data from the TriNetX US Collaborative Network were accessed through approved institutional account. The analyses were conducted using de-identified and aggregated data in accordance with the applicable TriNetX terms of use and institutional requirements. All secondary datasets used in this study were analyzed in accordance with the terms and conditions established by the respective data providers. No additional participant recruitment or collection of new biological samples was performed as part of this study.

#### Study design and database

**Fig. 1:**
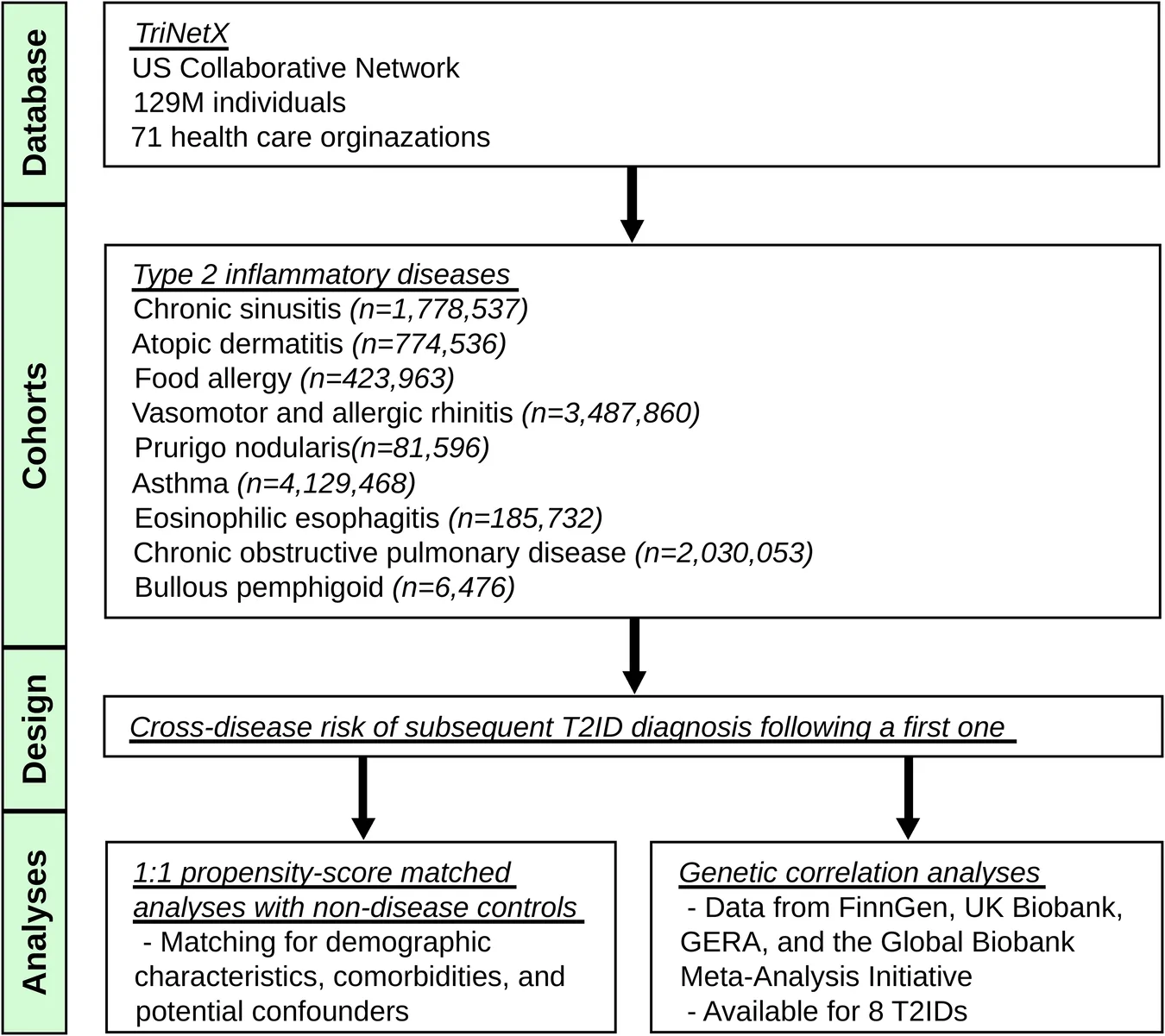
Flow chart of the study design and analytical framework. Patients with an incident diagnosis of one of nine type 2 inflammatory diseases (T2IDs) were identified in the TriNetX US Collaborative Network. Cohort sizes are shown before propensity-score matching. For each T2ID, the risk of subsequently developing each of the other T2IDs was assessed against controls without any T2ID. These cross-disease risks were complemented by genetic correlation analyses using publicly available genome-wide association summary statistics.

A propensity-score matched (PSM) retrospective cohort study was conducted using the US Collaborative Network from the federated EHR database TriNetX, following established protocols^21,22^. This network was selected due to the large number of documented EHRs and the high degree of covariate documentation^23^. Patients diagnosed with an incident diagnosis of the following T2IDs were identified: AD, PN, BP, asthma, VMR/AR, CS, COPD, food allergy and EoE. The risk for each T2ID to develop any of the other eight T2IDs was contrasted to that of non-T2ID controls, using PSM for better comparability. Four sensitivity analyses were performed to test the robustness of the findings, including an active comparator sensitivity analysis to probe the influence of healthcare engagement on the findings. In addition, negative control outcome analyses were performed to address residual confounding from factors not captured in the covariate matrix. Sex-, race- and age-stratified analyses were conducted to explore potential disparities relating to these factors. The analytical framework, definition of cohorts, matching factors and outcomes were defined prior to data collection (Fig. 1). The study was performed according to the STROBE guidelines^24^.

#### Study population

The study was conducted from March to May 2025. At the time of analysis, access to over 129 million patients across 71 HCOs in the United States was provided. Only EHRs from patients with incident T2IDs and no prior history of any other T2ID were included. T2IDs were categorised into dermatological T2IDs (AD, PN and BP), airway T2IDs (VMR/AR, CS, asthma and COPD), and gastrointestinal T2IDs (EoE and food allergy). All diseases were defined by their respective International Classification of Diseases, 10th Edition, Clinical Modification (ICD-10-CM) codes (Supplementary Table 1). To exclude miscoding of BP for pemphigus or other pemphigoid diseases, we excluded all EHRs with a documentation of any other pemphigus or pemphigoid disease (Supplementary Table 1). Non-T2ID controls were defined by Z00 (general healthcare encounter) and the absence of any T2ID prior to the healthcare visit. The same exclusions were applied as for cases. Index events were defined by the date of the incident T2ID diagnosis or, for controls, the date of the healthcare encounter.

#### Covariates

To minimise bias from potentially confounding variables, PSM was performed by establishing a covariate matrix that included demographic information, potential health hazards related to socioeconomic and psychosocial circumstances, and relevant concomitant diagnoses. Covariates included in all analyses were age at index (continuous), female sex (binary), self-reported White ethnicity (binary), potential health hazards related to socioeconomic and psychosocial circumstances (Z55-Z65, binary), overweight and obesity (E66, binary), and nicotine dependence (F17, binary). Matching was performed 1:1 using propensity-scores. Baseline characteristics were reported before and after matching (Supplementary Table 2). Reasons to include these covariates were: age is the single strongest determinant of T2ID incidence and progression, with sharply different risk profiles across childhood, adulthood and older age^25^. Sex differences in prevalence and severity are well-documented across T2IDs^26,27^. Self-reported White ethnicity was entered as a binary covariate in the propensity-score matching matrix because White individuals constituted the dominant demographic stratum in each T2ID and control cohort; this specification ensures balance on the principal demographic axis of the source population. Racial and ethnic heterogeneity was addressed separately and more directly through pre-specified subgroup analyses stratified by Black or African American, Hispanic or Latino and White ancestry, which allow group-specific effect sizes to be estimated without imposing exchangeability across strata. Socioeconomic and psychosocial health hazards are established determinants of both T2ID incidence and healthcare utilization and was included to mitigate socioeconomic confounding. We acknowledge that Z55–Z65 codes are known to be substantially under-documented in US EHRs, and therefore capture only a fraction of the true socioeconomic burden in the cohort^28^. Obesity is an established risk factor for several T2IDs and is also associated with altered immune function and differential healthcare engagement^29^. Nicotine dependence is a well-established driver of T2IDs and modifies disease trajectory across T2IDs^30^.

#### Outcomes

Outcomes were defined by the ICD-10-CM codes of the aforementioned T2IDs. For example, for AD, outcomes were PN (L28.1), BP (L12.0), VMR/AR (J30), CS (J32 or J33), asthma (J45), COPD (J44), EoE (K20.0) or food allergy (T78.0 or Z91.01). Any outcomes occurring prior to the index event were excluded during data retrieval. To probe residual confounding from factors not captured in the covariate matrix, we performed negative control outcome analyses. Fracture of clavicle (ICD-10-CM: S42.0) was pre-specified as the negative control outcome. For each of the nine T2ID index cohorts, we estimated the risk of clavicular fracture using the same PSM design and time windows as the primary analysis. The number of included EHRs in the negative control outcomes is higher because these were added during the review process. As such, more EHRs were captured in the live TriNetX setting.

#### Primary, sensitivity and subgroup analyses

In the primary analysis, outcomes occurring any time from 1 day after the index event were considered. In sensitivity analysis S1, to exclude already present but not diagnosed or coded outcomes at the index event, as well as to counter reverse causality, outcomes occurring any time from 3 months after the index event were considered. In S2, a minimal baseline time window of 6 months was mandated to ensure documentation of baseline characteristics and further mitigate detection bias. Like in the primary analysis, outcomes occurring any time from 1 day after the index event were considered. In S3, a stricter definition of the index event was used. In this analysis, a second documentation of the index event-defining criteria was required at least 6 months after the initial event. In S4, an active comparator sensitivity analysis was performed to address the possibility that differential healthcare engagement between exposed T2ID cohorts and Z00-based controls could influence the primary findings. Patients with incident hypothyroidism (E03.2/.3/.8/.9) and no prior history of any T2ID were selected as the active comparator cohort because hypothyroidism requires regular clinical encounters, laboratory monitoring and chronic disease management, but has no established pathophysiological link to type 2 inflammation. For each of the nine T2ID index cohorts, the primary analysis was re-run using this active comparator in place of the Z00-based control cohort, with the same covariate matrix and time windows. The subgroup analyses were stratified by age (1–12 years, 13–17 years or ≥18 years), sex (female, male) and self-reported ancestry (Black or African American, Hispanic or Latino or White).

#### Statistics and reproducibility

A propensity score for each patient was generated by logistic regression analysis (with exposure as the dependent variable) using the Python package scikit-learn. Matching was performed 1:1 using the greedy nearest neighbor approach with a cut-off distance of 0.1 pooled standard deviations of the logit of the propensity score. Baseline characteristics were re-evaluated and reported after matching; differences were compared by t-test for continuous and z-test for binary or categorical variables. Relative risks and risk difference (RD) were calculated. Survival analysis was performed using the Kaplan–Meier method in the Survival package v3.2-3 in R (R Foundation for Statistical Computing, Vienna, Austria) and validated by comparison with the outputs of SAS version 9.4 (SAS, Cary, NC). The proportionality assumption was tested by the coxph function in R’s Survival package. Kaplan–Meier-curves were compared using the Log-rank test. A univariate Cox proportional hazards regression was used to express hazard ratios (HR)s with 95% confidence intervals (CI)s. To account for multiple comparisons, *p*-values were corrected using the Bonferroni method (8 outcomes × 1 family per cohort), pre-defined. Sample sizes are indicated in the Supplementary Tables 2–21.

### Genetics

#### Ethics

The genetic analyses used exclusively publicly available, aggregate GWAS summary statistics; no individual-level genotype or phenotype data were accessed, and no new human data were generated.

#### Genetic correlation studies

Genome-wide association summary statistics for eight major T2IDs (AD, asthma, PN, EoE, CS, COPD, AR and BP) were obtained from large publicly available datasets, including the FinnGen consortium, UK Biobank, GERA and the Global Biobank Meta-Analysis Initiative (GBMI). AD data were retrieved from a meta-analysis including 60,653 cases and 804,329 controls^31^. For asthma: GWAS summary statistics were obtained from GBMI, comprising 121,940 cases and 1,254,131 controls from GERA^32^. For VMR/AR, summary statistics were obtained from both FinnGen (*n* = 70,158) and GERA^33^. Genetic data relating to COPD were drawn from GBMI^32^. For CS: GWAS summary statistics were obtained from FinnGen (16,395 cases; *n* = 299,737) and UK Biobank (3026 cases; *n* = 401,783). For EoE^34^ with 1930 cases and BP (FinnGen, Supplementary Table 3) with 507 cases, no sufficiently powered GWAS summary statistics were available. Hence, existing GWAS for the two diseases were used. Food allergy was not included in the genetic correlation analysis because of a lack of well-defined datasets. For asthma and AR, we conducted *p*-value weighted GWAS meta-analysis using METAL software. For each trait, we used GWAS summary statistics with European ancestry-based LD structure, and harmonised effect alleles and positions to GRCh37. Analyses were restricted to autosomal SNPs. All the GWAS summary statistics was aligned to the 1000 Genomes Project to correct for strand flips, allele mismatch and deviation from frequency from 1000 eur LD (>20%). We next estimated pairwise genome-wide genetic correlations using LD Score Regression (LDSC), implemented in ldsc v1.0.1. The 1000 Genomes Phase 3 European reference panel was used to calculate LD scores. SNP-based heritability (*h*²) was estimated for each trait using the same LDSC framework on the liability scale, using population prevalence estimates from published epidemiological studies (Table 1) and sample prevalence calculated from each study’s case-control ratio. The liability-scale transformation accounts for case-control ascertainment and expresses heritability on the underlying continuous liability distribution, enabling comparison across studies with different sampling schemes. LDSC intercepts and genomic control lambda (λGC) values were examined as quality-control metrics for residual confounding from population stratification or cryptic relatedness; intercepts close to 1.0 indicate well-controlled data. Heritability estimates, intercepts and λGC values are reported in Table 1. We note that rg is a symmetric measure of shared polygenic architecture and does not, by itself, encode causal or temporal directionality. We applied a Bonferroni correction across the 28 unique trait pairs to define significance thresholds, with *p* < 0.0018 (0.05/28) considered statistically significant. Note that the local genetic correlation analysis by LAVA, described below, uses a different multiple-testing framework (Benjamini–Hochberg FDR) appropriate to its higher-dimensional testing space. The presence of a significant genetic correlation (rg > 0) was interpreted as evidence of shared heritability between traits.

**Table 1:** SNP-based heritability and LD Score Regression quality-control metrics

Trait	*h*^²^ (liability)	SE	Intercept	SE	λ_GC	Pop. prevalence (*K*)	Sample prevalence (*P*)
Atopic dermatitis	0.055	0.007	1.031	0.009	1.121	0.20	0.075
Allergic rhinitis	0.076	0.006	0.940	0.008	1.105	0.20	0.060
Asthma	0.046	0.003	0.788	0.010	1.099	0.06	0.100
Chronic sinusitis	0.094	0.009	1.059	0.008	1.191	0.05	0.028
COPD	0.078	0.004	1.017	0.009	1.287	0.08	0.060
Eosinophilic esophagitis	0.097	0.020	1.210	0.008	1.250	0.00005	0.142
Bullous pemphigoid	0.112	0.085	0.989	0.006	1.014	0.00025	0.001
Prurigo nodularis	0.201	0.068	0.991	0.007	1.026	0.00083	0.002
Chronic urticaria	0.382	0.053	1.020	0.007	1.080	0.01	0.02

SNP-based heritability (*h*²) is reported on the liability scale, estimated by LD Score Regression (LDSC v1.0.1) using the population (*K*) and sample (*P*) prevalences shown. LDSC intercepts and genomic control lambda (λ_GC) are reported as quality-control metrics; intercepts near 1.0 indicate minimal confounding from population stratification or cryptic relatedness. Chronic urticaria was included in the local genetic correlation (LAVA) analysis as an additional type 2 inflammatory disease and was not part of the primary cohort study.

*SE* standard error.

#### Local genetic correlation

To localise the shared genetic signal underlying the T2ID for which sufficiently powered GWAS summary statistics were available (AD, asthma, COPD, VMR/AR, CS, EoE and chronic urticaria), we performed local genetic correlation analysis using LAVA (Local Analysis of [co]Variant Association)^35^. The trait set differed slightly from the global genetic correlation analysis described above: PN and BP were excluded from LAVA because their GWAS case counts (PN *n* = 606; BP *n* = 507) were too small for reliable estimation of local heritability at individual genomic loci, whereas chronic urticaria was included as an additional T2ID for which suitable GWAS summary statistics were available, although it was not analyzed in the retrospective cohort studies. The genome was partitioned into the default set of approximately 2500 nearly linkage-disequilibrium-independent blocks of ~1 Mb (GRCh37), using the 1000 Genomes phase 3 European reference panel for LD estimation. Sample overlap between GWAS was accounted for using the cross-trait intercepts from bivariate LD-score regression. At each locus, we first estimated the univariate local heritability of each disease; bivariate local genetic correlations were estimated only for trait pairs in which both diseases showed significant univariate local heritability at the default threshold (*P* < 0.05). Pairs not meeting this criterion at a given locus were not tested. This yielded 29,789 bivariate tests across 2485 loci for the 21 disease pairs. Local genetic correlations were considered significant at a false discovery rate (FDR) below 0.05, applying the Benjamini–Hochberg procedure across all bivariate tests performed. All analyses were conducted in LAVA (v0.1.5) in R (v4.5.3).

## Results

### Baseline characteristics

Data were sourced from a total of 129,702,895 EHRs. For the primary analysis, EHRs for patients diagnosed with incident T2IDs, specifically, CS (*n* = 1,778,537), AD (*n* = 774,536), food allergy (*n* = 423,963), VMR/AR (*n* = 3,487,860), PN (*n* = 81,596), asthma (*n* = 4,129,468), EoE (*n* = 185,732), COPD (*n* = 2,030,053) and BP (*n* = 6476), as well as between 13,108,725 and 13,421,991 for non-T2ID controls, were retrieved. Summarized baseline characteristics are found in Supplementary Table 2, while detailed characteristics for all cohorts are presented in Supplementary Tables 4–12. In all analyses, differences between groups observed prior to PSM were substantially reduced following matching.

### Chronic sinusitis is a central node in the T2ID comorbidity network

**Fig. 2:**
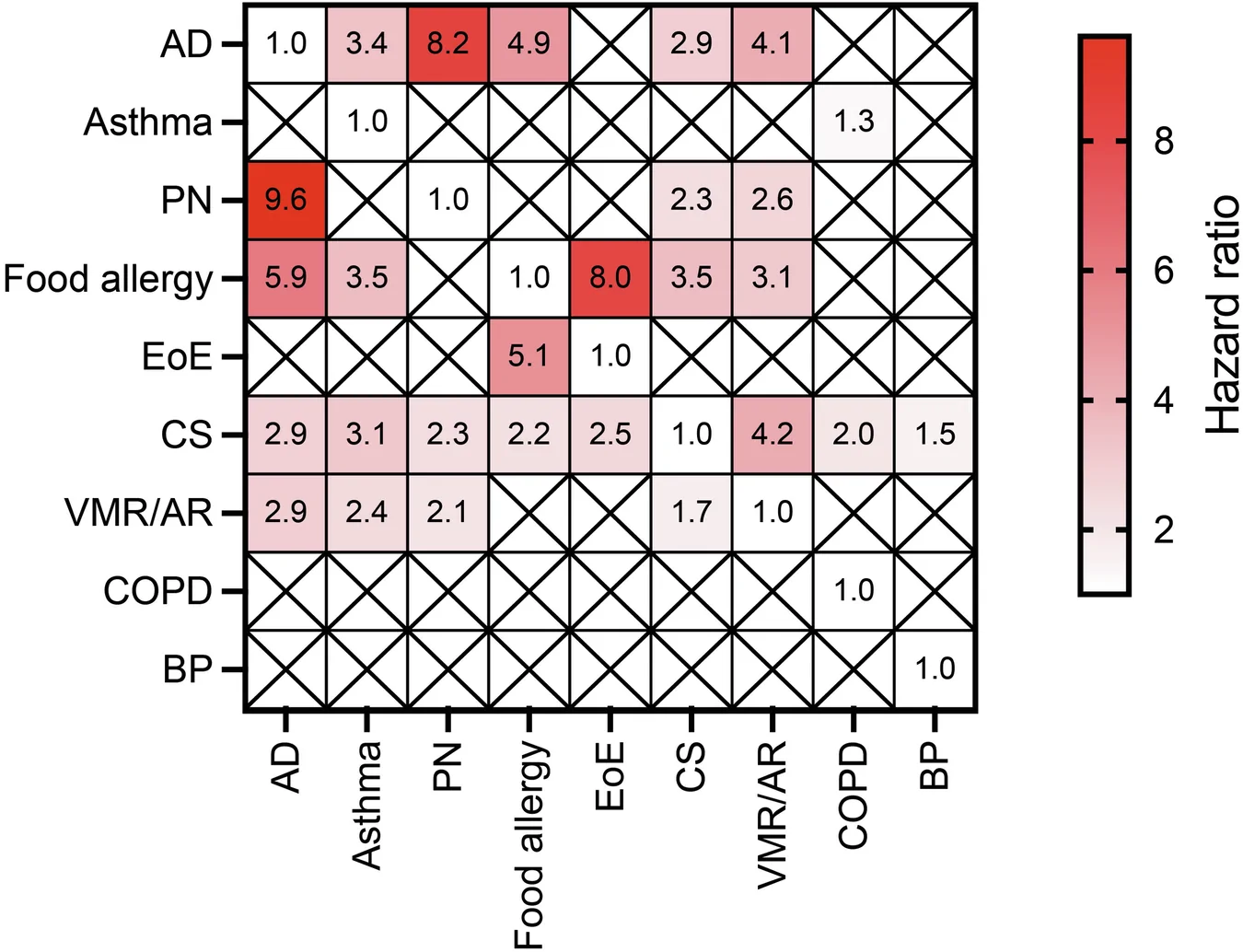
Cross-disease risk matrix of type 2 inflammatory diseases. Significant hazard ratios for incident type 2 inflammatory diseases (T2IDs) across all primary and sensitivity analyses (S1–S4). Each cell represents the HR from one T2ID (row) to another T2ID (column), calculated in longitudinal matched cohort analyses. Hazard ratios were estimated by univariate Cox proportional hazards regression in 1:1 propensity-score-matched cohorts; all tests were two-sided. Only statistically significant associations are shown after Bonferroni correction for multiple comparisons across the eight cross-T2ID outcomes assessed per exposure cohort (adjusted significance threshold *p* < 0.0063); nonsignificant comparisons are marked with an “X.” Analyses were conducted on the TriNetX electronic health record platform. AD, atopic dermatitis; PN, prurigo nodularis; VMR/AR, vasomotor and allergic rhinitis; EoE, eosinophilic esophagitis; CS, chronic sinusitis; COPD, chronic obstructive pulmonary disease; BP, bullous pemphigoid.

**Fig. 3:**
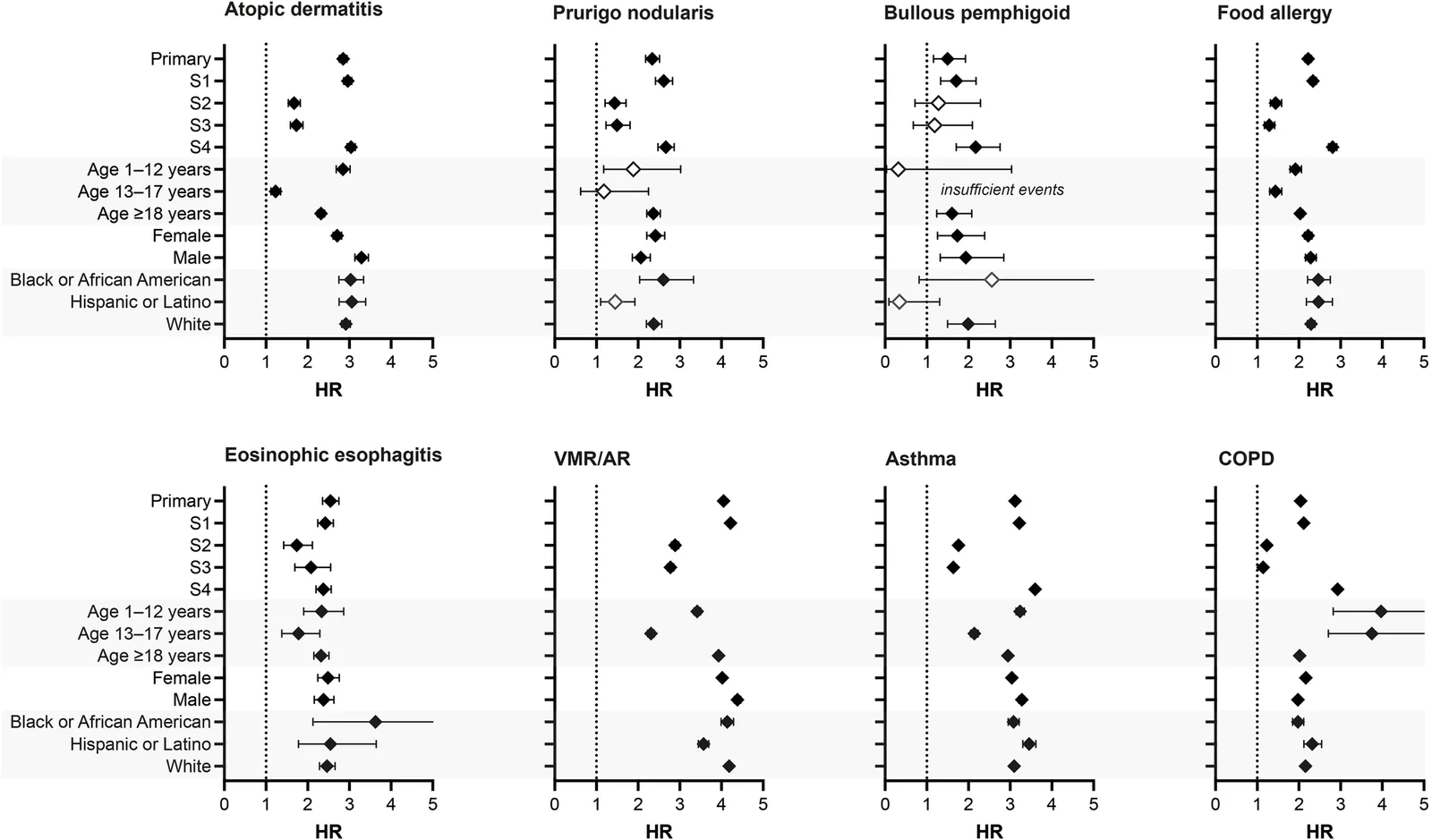
Risk of type 2 inflammatory diseases in patients with incident chronic sinusitis compared to non-type 2 inflammatory disease controls. Hazard ratios (diamonds) and 95% confidence intervals (error bars) for the risk of developing other type 2 inflammatory diseases (T2IDs) in patients with chronic sinusitis (CS) versus non-T2ID controls. Each panel represents the indicated T2ID as an outcome, and each row shows results from the primary analysis, sensitivity analyses (S1–S4), and subgroup analyses stratified by age, sex and race/ethnicity. Hazard ratios were estimated by univariate Cox proportional hazards regression in 1:1 propensity-score-matched cohorts; all tests were two-sided. Statistical significance was defined after Bonferroni correction for multiple comparisons across the eight cross-T2ID outcomes assessed per exposure cohort (adjusted threshold *p* < 0.0063). Black diamonds indicate statistically significant results; open diamonds represent non-significant results. Alternating row shading is used to visually separate subgroup levels. AD, atopic dermatitis; PN, prurigo nodularis; VMR/AR, vasomotor and allergic rhinitis; EoE, eosinophilic esophagitis; CS, chronic sinusitis; COPD, chronic obstructive pulmonary disease; BP, bullous pemphigoid.

In the primary analysis, incident CS was significantly associated with increased risk of seven of eight investigated downstream T2IDs: atopic dermatitis (HR 2.85, 95% CI 2.76–2.95, *p* < 0.0001), PN (HR 2.34, 2.18–2.51), asthma (HR 3.12, 3.08–3.16), VMR/AR (HR 4.05, 4.01–4.10), COPD (HR 2.043, 2.007–2.079), EoE (HR 2.55, 2.36–2.75) and food allergy (HR 2.22, 2.15–2.30). These associations persisted across all four sensitivity analyses. In the primary and S1 analyses, an increased risk for BP was noted. This, however, was not validated in sensitivity analyses S2 and S3 (Figs. 2, 3 and Supplementary Table 13).

Subgroup analyses revealed significant differences among stratified groups: for PN, which typically affects the elderly^36^, childhood or adolescent CS did not alter the risk of subsequent PN diagnosis, whereas in adults, CS increased this risk (HR 2.36, 2.21–2.53). Conversely, the risk for a subsequent COPD diagnosis was lower in adult CS patients (HR 2.02) compared to children or adolescents with CS (HR 3.98, or 3.75, respectively). By contrast, in the analyses stratified by either female of male risk, no impactful differences relating to the risk to be diagnosed with another T2ID following a CS diagnosis were noted. Racial disparities were noted for PN, where the highest risk increment imposed by CS was observed for those with Black or African American descent (HR 2.60), as opposed to White (HR 2.38) or Hispanic or Latino (HR 1.45) persons. Black or African American persons had also the highest risk for a subsequent EoE diagnosis (HR 3.63), compared to that of White (HR 2.46) or Hispanic or Latino (HR 2.55) persons (Fig. 3 and Supplementary Table 13).

### Atopic dermatitis, food allergy, and vasomotor and allergic rhinitis are secondary hubs in the T2ID network

AD also emerged as an important hub in the T2ID network, increasing the risk of six of the eight investigated T2IDs across the primary and all sensitivity analyses-specifically PN (HR 8.24, 7.12–9.55), VMR/AR (HR 4.16, 4.09–4.23), CS (HR 2.94, 2.85–3.03), asthma (HR 3.43, 3.37–3.50), EoE (HR 2.91, 2.55–3.32) and food allergy (HR 4.96, 4.77–5.15). In the primary analyses and in S1–2, AD was also associated with an increased risk for BP, EoE and COPD. However, in S3, this risk increment was not observed. Given the reciprocal risk increase between CS and AD, along with their high prevalence^37,38^, both conditions should be recognized as major contributors to the development and perpetuation of T2IDs (Fig. 3 and Supplementary Table 14). In the subgroup analyses stratified by age, major differences between childhood, adolescent and adult incident AD patients were noted. Regarding PN, the risk increment imposed by AD amounted to a HR of 9.22 (7.88–10.80) in adult AD patients, which was, albeit still increased, not as pronounced in childhood AD (HR 6.33, 4.49–8.93), and highest for adolescents (HR 10.63, 5.71–19.78). By contrast, the risks imposed for asthma and food allergy were comparatively higher in children with AD in contrast to adults (Supplement Table 12). When the analysis was stratified by sex, the risk imposed for other T2ID by AD was similar across both sexes, except for PN, with HR of 9.48 (7.62–11.79) in females and 8.39 (6.33–11.12) in males. Racial disparities were observed for the risks for PN (Hispanic or Latino <White <Black or African American) and food allergy (Black or African American <White <Hispanic or Latino) (Supplementary Table 14).

Food allergy also increased the risk for the subsequent diagnosis of five of the eight investigated T2IDs, namely, AD (HR 5.94), VMR/AR (3.18), CS (2.50), asthma (3.50) and EoE (8.01). These risks were observed in the primary and all three sensitivity analyses. Compared to adult patients with food allergy, a monotonic decline was noted in adolescents and children for AD and EoE risks. Regarding EoE, the HR in food allergy patients aged 1–12 years amounted to 9.82, to 9.30 in adolescents, while in those 18 years or older the HR for EoE was 5.39. In the subgroup analysis stratified by sex, no major differences in cross-T2ID risk were noted. When stratified by self-reported ancestry, racial disparities were noted. The risk of AD following a food allergy diagnosis was highest in those with Hispanic or Latino ethnicity (HR 6.10), as opposed to White (5.47) or Black or African American (5.14) individuals. Notably, for EoE, food allergy imposed the highest risk for EoE in Black or African Americans (8.67), while this risk increment was lower in Hispanic or Latinos (7.17) or Whites (6.10) (Supplementary Table 15).

Similarly, and across the primary and all sensitivity analyses, incident VMR/AR also increased the risk for four of the eight T2IDs investigated: AD, asthma, PN and CS, with HRs ranging from 2.10 for PN to 2.90 for AD. Significant increased risks for food allergy, EoE, COPD and BP were also noted in the primary and S1–2 analyses, but failed to replicate in S3. In the subgroup analysis, no major differences relating to this risk were observed (Supplementary Table 16).

### Prurigo nodularis also contributes to cross-T2ID risk

PN consistently increased the risk for the subsequent diagnosis of AD (HR 9.62, 8.25–11.26), CS (HR 2.39, 2.22–2.58) and VMR/AR (HR 2.63, 2.49–2.78) in the primary and all sensitivity analyses. Diagnosis of PN did not change the risk for the other investigated T2IDs. In the subgroup analyses, adults, but not none of the other two age-stratified analyses with PN demonstrated an increased risk for AD, CS and VMR/AR. Furthermore, quite substantial racial disparities were noted: in persons with Hispanic or Latino ancestry, PN increased the risk for a subsequent AD diagnosis (HR 4.17). This risk for AD was further increased in those with White (HR 8.26) and especially Black or African American persons (HR 10.26). No major sex differences regarding to the risk of AD, CS and VMR/AR were noted. Detailed information on these findings is presented in Supplementary Table 17.

### Asthma, eosinophilic esophagitis, COPD and bullous pemphigoid likely represent downstream manifestations of core T2IDs

Asthma increased the risk for a subsequent COPD diagnosis, which was most pronounced in children and in persons with self-reported Hispanic or Latino ethnicity (Supplementary Table 18). EoE increased the risk of food allergy, which was post pronounced in children (Supplementary Table 17). A diagnosis of COPD (Supplementary Table 20) or BP (Supplementary Table 21) was not associated with changes in the risk for any of the investigated T2IDs.

### Negative control outcome analyses

Pre-specified negative control outcome analyses using clavicular fracture as a biologically unrelated outcome detected null or near-null hazard ratios in seven of nine T2ID cohorts. Modest signals were observed for COPD (HR 1.21) and PN (HR 1.77) at the pre-specified Bonferroni threshold, indicating some residual confounding in these cohorts. In all nine cohorts, negative control HRs were substantially smaller than the corresponding cross-T2ID associations, indicating that residual confounding cannot account for the central findings (Supplementary Tables 13–21).

### Genetic correlation supports the comorbidity structure observed in the RWD and aligns with the central position of chronic sinusitis

**Fig. 4:**
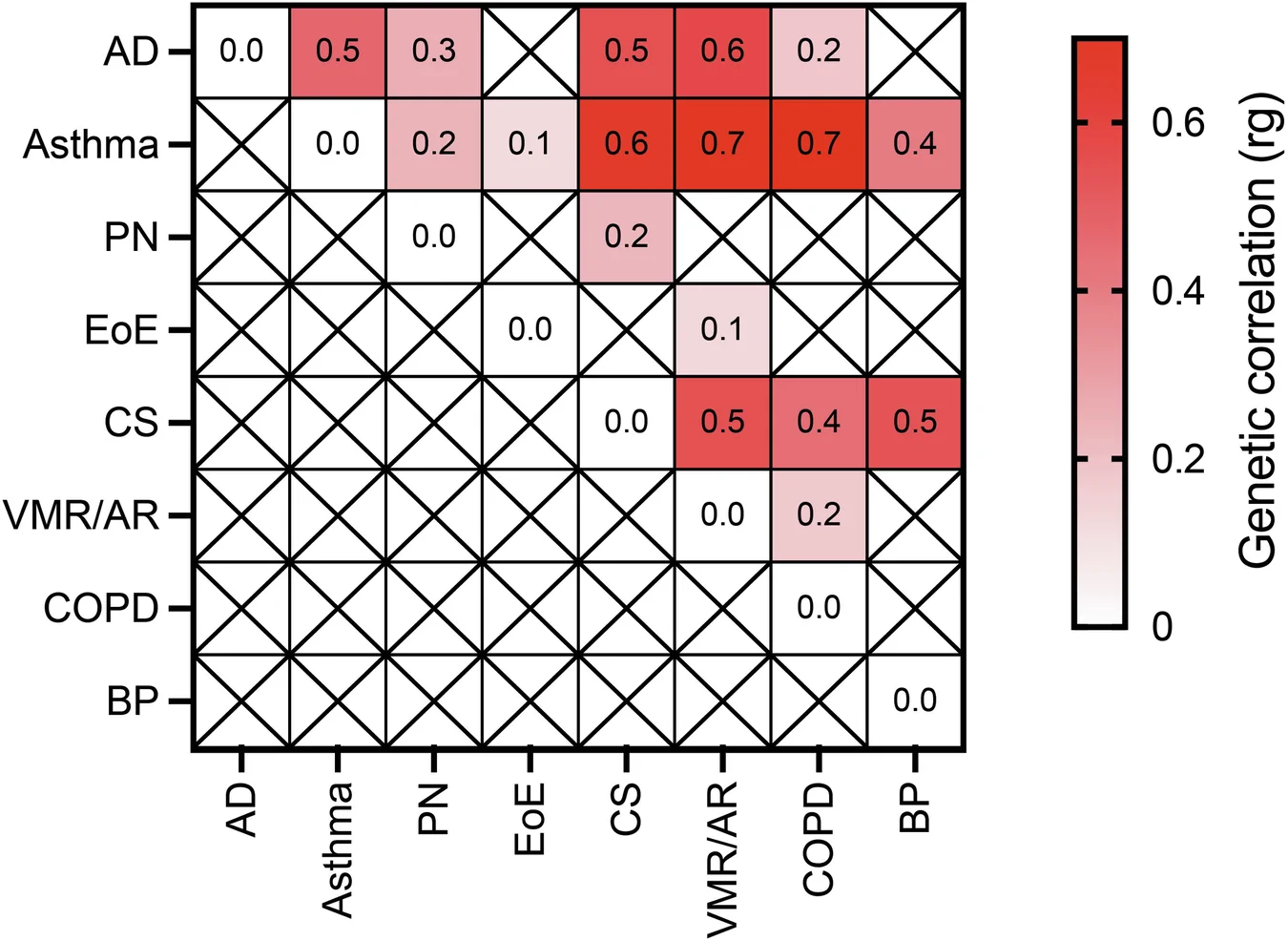
Genetic correlation matrix of type 2 inflammatory diseases. Pairwise genetic correlations (rg) between eight type 2 inflammatory diseases (T2IDs) estimated using LD Score Regression. Each cell indicates the magnitude of genetic correlation (rg) between the T2ID listed in the row and the T2ID listed in the column. Genetic correlations were estimated by LD Score Regression (LDSC v1.0.1) from GWAS summary statistics; all tests were two-sided. Only statistically significant correlations are shown after Bonferroni correction for multiple comparisons across the 28 unique trait pairs (adjusted threshold *p* < 0.0018); nonsignificant correlations are marked with an “X.” Colour intensity reflects the strength of rg, with darker red indicating higher correlation. Genetic correlation analyses were performed using publicly available GWAS summary statistics and European ancestry LD reference panels. AD, atopic dermatitis; PN, prurigo nodularis; VMR/AR, vasomotor and allergic rhinitis; EoE, eosinophilic esophagitis; CS, chronic sinusitis; COPD, chronic obstructive pulmonary disease; BP, bullous pemphigoid.

**Fig. 5:**
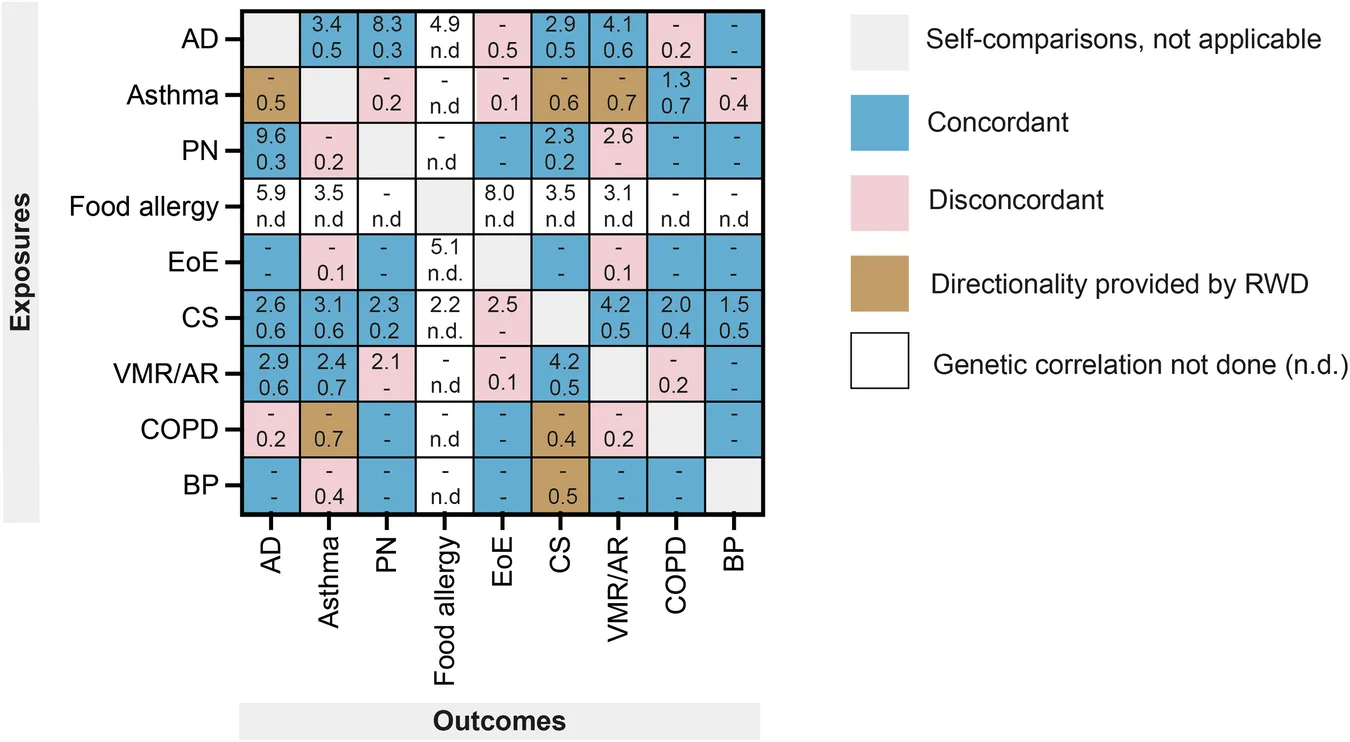
Cross-modal matrix of real-world and genetic correlations among type 2 inflammatory diseases. Each cell represents a disease pair, with the top number indicating the hazard ratio (HR) for the real-world risk of developing the outcome disease (based on retrospective electronic health record data) and the bottom number indicating the genetic correlation (rg) derived from LD Score Regression. Colour coding reflects the concordance of the associations: Blue indicates pairs with both a significant HR and a significant rg, representing a clinically observed temporal sequence accompanied by shared heritability; brown indicates pairs in which a directional association is supported by RWD only, with no significant rg detected; pink indicates discordant pairs, in which a significant rg was observed in the absence of a significant HR in either direction, or vice versa; white indicates that genetic correlation analysis was not performed due to the lack of sufficiently powered GWAS. Gray cells indicate self-comparisons. Hazard ratios were estimated by univariate Cox proportional hazards regression in 1:1 propensity-score-matched cohorts, and genetic correlations by LD Score Regression (LDSC v1.0.1); all tests were two-sided. Only statistically significant associations are shown after Bonferroni correction for multiple comparisons (*p* < 0.0063 for HRs, across the eight cross-T2ID outcomes assessed per exposure cohort; *p* < 0.0018 for rg, across the 28 unique trait pairs). Non-significant comparisons are marked with a dash (-), and missing genetic data are denoted as “n.d.” AD, atopic dermatitis; BP, bullous pemphigoid; COPD, chronic obstructive pulmonary disease; CS, chronic sinusitis; EoE, eosinophilic esophagitis; PN, prurigo nodularis; VMR/AR, vasomotor and allergic rhinitis.

To validate and extend the findings from our retrospective cohort studies on cross-disease risks among T2ID, we next performed genetic correlation analyses. These were based on previously published GWAS meta-analyses as well as three newly conducted meta-analyses for AR, asthma and CS. Detailed information on the non-FinnGen GWAS datasets is provided in Supplementary Data 1–4. In detail, we compared results from retrospective cohort analyses with genetic correlations for the 56 possible cross-disease comparisons. Among these, 34 combinations (61%) showed concordant signals, with both HR and rg being statistically significant and pointing in the same direction. In six of the comparisons (11%), a significant genetic correlation was observed, while only one direction of clinical association reached significance (Fig. 4). In these pairs, the RWD identified a significant temporal association in one direction only, while the symmetric genetic correlation indicated shared heritability; together, these results identify disease pairs in which a genetically correlated trait pair is accompanied by a clinically observed temporal sequence, without implying causality. In 16 of the comparisons (28%), the results were discordant. In 15 of these, a significant genetic correlation was observed in the absence of a significant HR in either direction. In two pairs, the inverse was true, with a significant HR but no detectable genetic correlation. Notably, most of these discordant significant genetic correlations were of modest magnitude. Together, these findings suggest that retrospective cohort studies may be more conservative in detecting disease associations compared to genetic correlation analyses, particularly when genetic overlap is limited (Fig. 5).

We next examined chronic sinusitis in more detail, given its central role in driving the T2ID comorbidity network. To continue with chronic sinusitis, we observed that genetic correlations aligned well with the increased risks identified in the retrospective cohort data for several diseases, including asthma, AD, PN, VMR/AR, COPD and BP. Notably, RWD also revealed an increased risk of EoE in patients with chronic sinusitis, which was not reflected in the genetic correlation analysis. In several instances, the temporal ordering observed in the RWD was accompanied by a significant shared polygenic architecture, supporting the biological plausibility of the clinical sequence. For example, CS and asthma share a strong genetic correlation (rg = 0.6), and RWD showed that patients with CS have an increased risk of subsequently developing asthma (HR 3.12, 3.087–3.16). In contrast, asthma does not confer a significantly increased risk for CS (HR 1.30, 1.29–1.32). The temporal ordering observed in the RWD, in the presence of a strong shared genetic architecture, is consistent with a clinical sequence in which CS typically precedes asthma, though causal inference cannot be made from these data.

### Local genetic correlation identifies shared mechanistic loci and positions asthma as the central node of the polygenic architecture

**Fig. 6:**
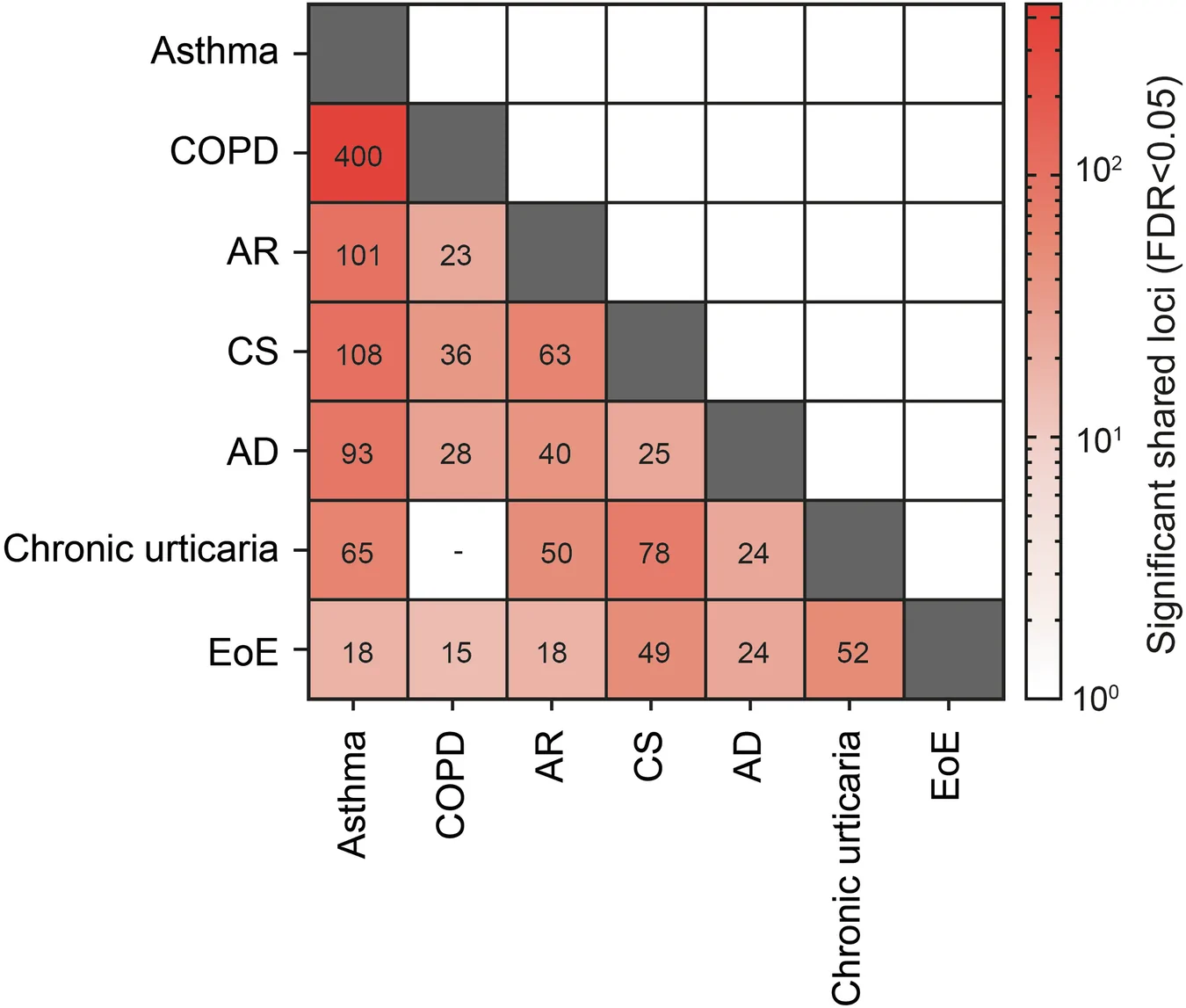
Local genetic correlation across type 2 inflammatory diseases identifies asthma as a central node in the shared polygenic architecture. Number of FDR-significant bivariate local genetic correlations per disease pair across seven type 2 inflammatory diseases (LAVA, Benjamini–Hochberg FDR < 0.05, 29,789 bivariate tests across 2485 LD-independent genomic blocks). Local genetic correlations were estimated using LAVA; all tests were two-sided. Bivariate correlations were tested only at loci where both diseases passed the univariate local heritability filter (*P* < 0.05), and significance was defined by Benjamini–Hochberg false discovery rate control (FDR < 0.05) across all 29,789 bivariate tests. Colour intensity (log scale) indicates the number of shared FDR-significant loci. Dark grey cells are self-comparisons; a dash indicates a pair with no locus at which both traits met the univariate heritability criterion (COPD and chronic urticaria). AD, atopic dermatitis; AR, allergic rhinitis; COPD, chronic obstructive pulmonary disease; CS, chronic sinusitis; EoE, eosinophilic esophagitis.

To complement the global genetic correlation analysis with locus-level resolution, we performed local genetic correlation analysis using LAVA for the seven T2IDs for which sufficiently powered GWAS summary statistics were available. Across 2485 LD-independent genomic blocks and 21 disease pairs, 1310 bivariate associations reached statistical significance at the pre-specified FDR threshold (FDR < 0.05, Benjamini–Hochberg), distributed across 852 distinct loci. The shared local genetic signal was predominantly concordant: 1163 of the 1310 significant correlations (89%) were positive, with a median local rg of 0.61. The number of FDR-significant shared loci differed substantially across disease pairs, with asthma–COPD showing the largest number (400 loci), followed by asthma–CS (108), asthma–VMR/AR (101) and asthma–AD (93). Asthma was involved in the largest number of shared loci with every other T2ID examined, positioning it as the central node of the shared polygenic architecture (Fig. 6A) and complementing the central position of CS in the temporal clinical network derived from the RWD analyses. Shared local genetic signal converged on canonical T2ID loci, including the *GATA3* region (10p14, significant for all seven diseases), the 17q12-21 asthma locus (*ORMDL3/GSDMB/IKZF3*, seven diseases), the alarmin/cytokine genes TSLP (5q22), *IL1RL1/IL18R1/IL18RAP* (2q12, encoding the IL-33 receptor *ST2*), *STAT6* (12q13.3), the 11q13.5 atopy locus (*LRRC32/C11orf30*), and the major histocompatibility complex. The 5q31.1 cytokine cluster (chr5:130.6–132.6 Mb; *IL4*, *IL5*, *IL13*, *CSF2*, *RAD50*, *IRF1*) showed eight FDR-significant, uniformly positive local genetic correlations, with rg values ranging from 0.40 (asthma–CS) to 0.74 (asthma–COPD) and including asthma–AD (rg = 0.70, *P* = 1.3 × 10⁻²⁹) and VMR/AR–asthma (rg = 0.64, *P* = 1.5 × 10⁻¹⁵). The convergence of the shared genetic signal onto canonical T2ID pathways, including alarmins (*TSLP, IL-33/ST2*), Th2 lineage regulators (*GATA3, STAT6*, 17q12-21), and effector cytokines (5q31.1 *IL4/IL5/IL13* cluster), provides locus-level mechanistic support for the shared polygenic architecture inferred from the global LDSC analyses^39^.

## Discussion

In this study, we used two independent and complementary approaches, namely retrospective cohort analyses from a large-scale EHR database and both global and local genetic correlation analyses, to systematically map comorbidity patterns across nine major T2IDs. Our findings provide an in-depth description of the interrelated network of disease trajectories amongst T2IDs. This network-based view goes far beyond the classical concept of the atopic march, which describes a unidirectional sequence from AD to asthma and AR^11^. Instead, our data reveal that the atopic march represents just one of many possible comorbidity trajectories within a broader, more heterogeneous spectrum of T2ID interactions that span multiple organ systems and diverse disease pairings. Within this network, CS emerged as a central hub in the temporal clinical network derived from the RWD, significantly increasing the risk for nearly all other T2IDs examined. The mean age at CS index (42.7 years) lies in the middle of the T2ID age distribution, and CS-associated cross-T2ID risks persist across childhood, adolescent and adult age strata, indicating that the central network position of CS is not attributable to age-related characteristics of the cohort. AD, food allergy and VMR/AR also contributed to network propagation, albeit with smaller impact. These observations remained robust across multiple sensitivity analyses and diverse patient subgroups.

The traditional concept of the atopic march describes a sequential progression of T2IDs, starting with AD in infancy, followed by food allergies, AR and asthma. This model has been instrumental in understanding the development of T2IDs^11^. However, emerging research suggests that this linear trajectory does not encompass the full complexity of T2ID progression^16,40,41^. Furthermore, the concept of the atopic march exclusively focuses on disease trajectories in childhood, with T2ID comorbidity patterns in adulthood being less clear^41^. Our results contribute to this evolving understanding, demonstrating a complex and intricate network of cross-disease trajectories among T2IDs, extending beyond the traditional atopic march. Notably, our data identify CS as a central node within the temporal clinical network of T2IDs, significantly increasing the risk for multiple downstream T2IDs. This observation challenges the conventional view that positions AD as the primary initiator of the T2ID comorbidity trajectory.

Our genetic correlation analyses revealed a complementary but distinct view of the network. While CS occupied the central position in the temporal clinical network, local genetic correlation analyses by LAVA identified asthma as the central node in the shared polygenic architecture, with the largest number of FDR-significant local genetic correlations to other T2IDs (400 with COPD, 108 with CS, 101 with VMR/AR, 93 with AD). These observations are not contradictory but describe different layers of the same disease network. The genetic correlation analyses identify shared polygenic susceptibility, which is non-directional and reflects co-inherited risk. The temporal RWE network reflects the order in which T2IDs become clinically apparent in patient trajectories. The shared local genetic signal converged on canonical components of the type 2 inflammatory pathway, including alarmins (*TSLP* at 5q22, the *IL1RL1/IL18R1/IL18RAP* cluster encoding the *IL-33* receptor ST2 at 2q12), Th2 lineage regulators (*GATA3* at 10p14, the 17q12-21 locus comprising *ORMDL3/GSDMB/IKZF3, STAT6* at 12q13.3), and effector cytokines (the 5q31.1 cluster encoding *IL-4, IL-5 and IL-13*)^39^. This convergence at canonical Th2 pathway nodes provides locus-level mechanistic support for the shared genetic architecture and offers a biological interpretation of the temporal clinical sequence: asthma anchors the genetic axis at the Th2 cytokine cluster, while CS is often the earliest clinically apparent manifestation of this shared susceptibility.

Importantly, our study incorporates stratified analyses by age, sex and race/ethnicity, revealing marked disparities in disease trajectories. For example, adults with CS or AD had substantially higher risks for developing PN or COPD, while children with food allergy showed an elevated risk for developing EoE. Racial disparities were also pronounced: Black or African American individuals, compared to White or Hispanic/Latino individuals, often displayed the highest cross-disease risks, particularly for PN and EoE, compared to other racial groups. These findings align with a higher PN incidence in Black or African American individuals compared to Whites^42^, but contrast findings in EoE that has an overrepresentation of White individuals^43^. This discrepancy may suggest that racial disparities in T2IDs differ between incident disease risk and subsequent disease manifestation within comorbidity trajectories. These findings reinforce the need to consider patient history as well as demographic context when evaluating disease progression and risk.

To determine whether observed comorbidities reflect shared etiologic mechanisms or simply co-occurrence, we complemented RWD analyses with genetic correlation studies. Across 56 possible disease pairs, the majority showed concordant associations in both data modalities, supporting shared heritability and potential biological connections. In a few cases, only one clinical direction was significant, but genetic correlation provided evidence of shared architecture, enabling inference of the temporal sequence observed clinically, while shared heritability remains symmetric. Some comparisons yielded discordant findings. Most of these involved a significant genetic correlation in the absence of a clinical signal, suggesting that genetic methods may be more sensitive to low-penetrance or latent disease relationships. Alternatively, these observed genetic correlations without RWD validation may reflect a shared genetic architecture due to pleiotropy, co-inheritance or polygenic overlap, but without any mechanistic links amongst the two diseases.

Our study has several key strengths. First, it leverages two independent and complementary methodological approaches: large-scale, RWD from over 129 million individuals and both global and local genetic correlation analyses. Second, the findings were validated across multiple sensitivity analyses and stratified by age, sex and race/ethnicity, allowing for robust assessment of disparities and generalisability. Third, the integration of global and local genetic correlation analyses enables insights into both genome-wide shared heritability and the specific genomic loci at which susceptibility converges, while informing inference of potential directionality where observational data alone may be ambiguous. Fourth, bidirectional Mendelian randomization supports a causal effect of CS on asthma^44^ concordant with the temporal sequence observed here. For atopic dermatitis, the predominant genetic causal direction identified by Mendelian randomization runs from AD toward CS, with no significant reverse effect^45^, differing from the temporal order in our cohort data and underscoring that genetic causal direction and clinical temporal sequence are distinct constructs. For the rarer T2IDs (PN, BP and EoE), causal inference by Mendelian randomization remains precluded by GWAS power and is an important direction for future work.

Nonetheless, several limitations should be considered when interpreting these findings: first, the study retrospectively analyzed RWD and is therefore subject to potential bias from unmeasured confounders. A key concern is the definition of incident T2IDs, as incident diagnoses are based on the first documentation at a participating HCO within the TriNetX network. This approach may fail to capture prior diagnoses made outside the network. Miscoding, i.e. COPD in up to 0.2% of adolescents, is another potential bias. In addition, RWD sources are susceptible to miscoding of ICD codes and other inaccuracies. Healthcare access disparities may also influence the documentation of comorbidities, potentially masking measured socioeconomic differences. Finally, while our findings demonstrate consistent associations between specific T2IDs, both clinically and genetically, they do not imply causality. Regarding the genetic correlation analysis, the limited sample sizes for BP and EoE GWAS likely resulted in reduced heritability estimates and diminished power to detect significant genetic correlations. Although temporal sequencing in RWD and concordant genetic correlations increase the biological plausibility of certain clinical sequences (e.g. CS preceding asthma), genetic correlation reflects shared heritability and does not, by itself, indicate causal or directional relationships. Residual confounding cannot be fully excluded, specifically for self-reported race and ethnicity because they are imperfect proxies for genetic ancestry and socioeconomic circumstances. To probe for residual confounding, we performed negative control outcome analyses using clavicular fracture as the outcome; signals were detected for COPD (HR 1.21) and PN (HR 1.77) at the pre-specified Bonferroni threshold, indicating that some residual confounding is present, particularly in the PN cohort. The magnitudes of these signals are, however, substantially smaller than the primary cross-T2ID associations and cannot account for the central findings. It is also important to emphasize that genetic correlation reflects shared polygenic architecture between traits and is inherently symmetric; it does not imply a causal or temporal direction. Temporal ordering in our study is derived exclusively from the RWD analyses, and concordance between the two modalities should be interpreted as biologically plausible co-occurrence with an observed clinical sequence, rather than as evidence of causation. Future studies using Mendelian randomization, longitudinal designs and mechanistic experimentation are needed to determine causal relationships more definitively.

Our findings underscore that T2IDs form a complex and tightly interwoven network of cross-disease trajectories. Within this network, CS is a key upstream node of downstream T2ID comorbidities in the clinical sequence, while asthma anchors the shared polygenic architecture at canonical Th2 pathway loci. CS, and to a lesser extent also AD, food allergy and VMR/AR, may serve as early indicators (and actionable targets) for preventive intervention to halt emerge of further T2ID. Crucially, the identified age-, sex- and race-specific disparities in comorbidity patterns highlight the need for personalized risk stratification and more equitable, precision-driven approaches in patients diagnosed with incident T2ID.

## Supplementary information

Transparent Peer Review File

Supplemental Information

Supplementary Data 1

Supplementary Data 2

Supplementary Data 3

Supplementary Data 4

Supplementary Data 5

## Data Availability

*Real-world data.* The retrospective cohort analyses were performed on de-identified electronic health records from the TriNetX US Collaborative Network. These data are the property of TriNetX LLC and its participating healthcare organizations and cannot be shared publicly or deposited in a repository. All aggregate results underlying the cohort analyses are provided in Supplementary Tables 1–21 and in the Source Data file. *Genetic data*. Publicly available GWAS summary statistics were used for atopic dermatitis (GWAS Catalog accession GCST90244787), eosinophilic esophagitis (GCST90027899), and allergic rhinitis (GCST90038664), all available at https://www.ebi.ac.uk/gwas/. Summary statistics from the Global Biobank Meta-analysis Initiative (asthma, chronic obstructive pulmonary disease) are available at https://www.globalbiobankmeta.org/. GERA data are available through dbGaP (accession phs000674). UK Biobank data are available to approved researchers via application at https://www.ukbiobank.ac.uk/. FinnGen-derived summary statistics (prurigo nodularis, bullous pemphigoid, chronic sinusitis, allergic rhinitis, and chronic urticaria) were used under a data use agreement and cannot be redistributed by the authors; they are available through the FinnGen consortium at https://www.finngen.fi/en/access_results. *Data generated in this study*. Summary statistics from the three new GWAS meta-analyses conducted here (asthma, chronic sinusitis, allergic rhinitis), and the full LD Score Regression and LAVA output, are available from the corresponding author on reasonable request. Because these meta-analyses incorporate FinnGen-derived summary statistics, release of the meta-analysed data is subject to the FinnGen data use terms and may require the requester to hold their own FinnGen data access approval. Source data underlying Figs. 2–6 are provided in Supplementary Data 5.
