# Supplementary material for: Trajectory and genetic correlation mapping of cross-disease risks following incident type 2 inflammatory diseases
Source: Commun Med (Lond). 2026 Nov 6;6:578. doi: 10.1038/s43856-026-02000-y (PMC13743556; doi:10.1038/s43856-026-02000-y)
Supplement: Supplementary file 1 [file 43856_2026_2000_MOESM1_ESM.pdf]

nature portfolio

Peer Review File

Trajectory and genetic correlation mapping of cross-disease risks following incident type 2 inflammatory diseases

Corresponding Author: Professor Ralf Ludwig

This file contains all reviewer reports in order by version, followed by all author rebuttals in order by version.

Version 0:

Reviewer comments:

Reviewer #1

(Remarks to the Author)
This manuscript integrates TriNetX EHR data with genome-wide genetic correlation analyses to investigate cross-disease risks among major T2IDs. The integrative framework and large-scale dataset are strengths. However, several methodological clarifications are required.
1. In lines 122–123 “(iii) provide direction of observed genetic correlations by integration with RWD,” , the manuscript states that the dual approach provides the “direction” of genetic correlations. This claim is not supported by the LDSC analysis described in lines 224–225 “We next estimated pairwise genome-wide genetic correlations using LD Score Regression (LDSC), implemented in ldsc v1.0.1.”. LDSC appropriately estimates genetic correlation (rg) as a measure of shared polygenic architecture (pleiotropy) between two traits, but rg is symmetric and does not encode causal or temporal direction. The manuscript should therefore revise the wording around “directionality” throughout, and restrict the interpretation of LDSC results to shared genetic basis. If the authors aim to make directional (causal) statements, additional causal inference methods (e.g., bidirectional Mendelian randomization) would be needed. If the intended meaning is temporal ordering in the EHR analysis, this should be stated explicitly and kept conceptually separate from genetic correlation.
2. In lines 169–170 “self reported White ethnicity (binary)”, race/ethnicity adjustment in the propensity score model appears limited to a binary “White vs. non-White” variable. For a heterogeneous US-based cohort, this specification is likely insufficient and may introduce residual confounding, particularly given that subgroup-specific risks are later reported. The covariate structure should be reconsidered or explicitly acknowledged as a limitation.
3. In line 189 “The subgroup analyses were stratified by age (1-12 years, >18 years)”, age subgroups are described as “1–12 years” and “>18 years,” with no mention of adolescents aged 13–17. This omission requires clarification, as adolescence is clinically relevant for several T2IDs.
4. In lines 207–208 “Genome-wide association summary statistics for seven major T2IDs (AD, asthma, PN, EoE, CS, COPD, AR and BP)”, the text refers to “seven” diseases while listing eight traits. This discrepancy should be corrected.
5. Lines 226–228 “We applied a Bonferroni correction for multiple testing to define significance thresholds. Pairwise correlations with FDR-adjusted p-values < 0.05 were considered statistically significant.” reference both Bonferroni correction and FDR-adjusted p-values as criteria for significance. These represent different multiple-testing frameworks and should not be used interchangeably. The statistical correction strategy must be clearly specified and applied consistently.
Overall, clarification of inferential boundaries and statistical consistency is necessary to strengthen the manuscript.

Reviewer #2

(Remarks to the Author)
The article by Gupta et al. provides an in-depth view of comorbidity relationships between type 2 inflammatory diseases. The manuscript demonstrates a convincing multimorbidity structure with novel findings, challenging the linear trajectory of the atopic march. The integration of large-scale epidemiologic data with genetic data is ambitious and conceptually valuable, and the work is likely to serve as a hypothesis-generating framework in type 2 inflammation across diverse fields. My primary concern relates to the somewhat strong language used by the authors on the interpretation relative to the evidentiary support provided by the analyses. Several limitations should be explicitly acknowledged and addressed whenever possible.
Major comments
--The manuscript repeatedly interprets asymmetric HRs as evidence for directional influence. This interpretation is not fully supported through the provided analytical framework, since HR asymmetry can arise from multiple non-causal mechanisms. Despite this is properly acknowledged at lines 425-428, stronger causal or upstream framing persists elsewhere, as for

example at lines 246-247, 355-357 and 401-402. I recommend moderating causal language and reframing these findings as directional associations rather than upstream disease effects.
--It is well-known that patients with chronic inflammatory diseases have higher healthcare engagement (PMID: 31906907, 40266617, 32266326), thus increasing diagnostic probability of additional conditions independent of biological risk. The propensity score model does not appear to control for any similar variable such as clinical encounter and diagnostic testing frequency among others. Since I suspect that such data are absent in the available dataset, the authors should include negative control outcomes like age-related cataract and non-atopic conditions (e.g., acne vulgaris, varicose veins) and/or negative control exposures like vaccination history and screening procedures to probe confounding by healthcare engagement and identify detection bias.
--The manuscript states that LD score regression was used to estimate heritability but does not report SNP-based heritability estimates or LDSC intercepts. Reporting these metrics, preferably in Table 2 on the liability scale, would improve transparency and interpretability of the genetic analyses.
-- There are multiple genetic-based evidence that could enhance the interpretation of the epidemiological findings of the present manuscript. Nevertheless, most of them are out of scope of the current work. However, since the authors do claim about shared genetic architecture, I would advise them to run local genetic correlations to investigate whether specific loci are consistently associated with all T2IDs. If, for example, the cytokine cluster at the 5q31-33 region shows significant genetic correlations, this would provide some mechanistic evidence about the findings.

Minor comments
--While the authors appropriately note limited power for Mendelian randomization due to GWAS sample size constraints at lines 424-425, the manuscript's central claims regarding directionality would benefit from causal inference analyses for at least the most central results. This is even more prominent at lines 104-106, where the authors acknowledge the lack of published data for T2ID combinations. Even exploratory Mendelian randomization analyses would substantially strengthen the interpretation of directional relationships. I do acknowledge that some stratified analysis findings cannot be validated without individual-level genetic data.
--At lines 226-227, the authors state using Bonferroni correction for LDSC results, while at lines 227-228 the significance of pairwise genetic correlation estimates is defined as FDR-adjusted p-values < 0.05. Finally, at lines 228-229, "the presence of a significant genetic correlation (rg>0) was interpreted as evidence of shared heritability between traits". It is not clear what the significance threshold of LDSC results was.
--The authors could in fact visualize the genetic correlation estimates using LDSC networks (e.g., https://github.com/mkanai/ldsc-network-plot) and combine with HR estimates to improve clarity of Figure 5, although consistency between Figure 2, Figure 4 and Figure 5 may be more important for the authors.
--I appreciate the authors' effort to provide colorblind-friendly images, and especially Figure 5. However, the white letters at Figure 5, concordant associations are not that much visible; kindly recolor them to black.

Reviewer #3

(Remarks to the Author)
This is a well written manuscript with interesting findings. The analyses is complex, but appears to answer their question. My minor comments are below.

Line 105: Abbreviations should be spelled out
Line 164: Control definition should include excluding those with the atopic disease
Line 174: shouldn't family history be considered a covariate?
Line 245: could the finding of chronic sinusitis be related to age of the patients? This is commonly

Version 1:

Reviewer comments:

Reviewer #2

(Remarks to the Author)
The manuscript has been substantially improved following recommendations from all reviewers. I recommend acceptance.

Reviewer #3

(Remarks to the Author)
I believe the authors have done a good job of answering my comments/questions, and I approve this manuscript.

Open Access This Peer Review File is licensed under a Creative Commons Attribution 4.0 International License, which permits use, sharing, adaptation, distribution and reproduction in any medium or format, as long as you give appropriate credit to the original author(s) and the source, provide a link to the Creative Commons license, and indicate if changes were made.
In cases where reviewers are anonymous, credit should be given to 'Anonymous Referee' and the source.
The images or other third party material in this Peer Review File are included in the article's Creative Commons license, unless indicated otherwise in a credit line to the material. If material is not included in the article's Creative Commons license and your intended use is not permitted by statutory regulation or exceeds the permitted use, you will need to obtain permission directly from the copyright holder.
To view a copy of this license, visit https://creativecommons.org/licenses/by/4.0/

Reviewer #1
1. In lines 122–123 “(iii) provide direction of observed genetic correlations by integration with RWD,” the manuscript states that the dual approach provides the “direction” of genetic correlations. This claim is not supported by the LDSC analysis described in lines 224–225 “We next estimated pairwise genome-wide genetic correlations using LD Score Regression (LDSC), implemented in ldsc v1.0.1.”. LDSC appropriately estimates genetic correlation (rg) as a measure of shared polygenic architecture (pleiotropy) between two traits, but rg is symmetric and does not encode causal or temporal direction. The manuscript should therefore revise the wording around “directionality” throughout and restrict the interpretation of LDSC results to shared genetic basis. If the authors aim to make directional (causal) statements, additional causal inference methods (e.g., bidirectional Mendelian randomization) would be needed. If the intended meaning is temporal ordering in the EHR analysis, this should be stated explicitly and kept conceptually separate from genetic correlation.
We thank the Reviewer for this important conceptual clarification and fully agree. LDSC-derived genetic correlations (rg) are symmetric and reflect shared polygenic architecture rather than causal or temporal direction. We have revised the manuscript to separate these concepts clearly throughout. Specifically, in the Introduction (previously lines 122–123), we have replaced "provide direction of observed genetic correlations by integration with RWD" with: "integrate symmetric genome-wide genetic correlations with the temporal ordering available from retrospective cohort analyses, such that disease pairs with both shared heritability and a consistent clinical temporal sequence can be interpreted with greater confidence than either methodology alone."<br><br>In the Methods, we now explicitly state that "rg is a symmetric measure of shared polygenic architecture between two traits and does not, by itself, encode causal or temporal directionality.", and explicitly address this as a limitation in the Discussion by stating: “It is also important to emphasize that genetic correlation reflects shared polygenic architecture between traits and is inherently symmetric; it does not imply a causal or temporal direction. Temporal ordering in our study is derived exclusively from the RWD analyses, and concordance between the two modalities should be interpreted as biologically plausible co-occurrence with an observed clinical sequence, rather than as evidence of causation.”.<br><br>In the Results and Discussion, we have replaced "directional interpretation," "directionality," and "upstream" (when applied to the combined RWD+rg findings) with either "temporal ordering in RWD" or "directional association observed in RWD," depending on context. "Shared heritability" and "shared polygenic architecture" are now used exclusively for the

genetic correlation findings. We have also added a sentence to the Discussion emphasizing that formal causal inference - in particular bidirectional Mendelian randomization - would be required to support causal directional claims, and that such analyses are currently constrained by GWAS sample sizes for several T2IDs in our panel (see also our response to Reviewer #2, point 5).

We have additionally extended the genetic correlation analyses to include locus-level local genetic correlation by LAVA (see also response to Reviewer #2, Comment 4). Like LDSC, LAVA yields symmetric estimates of shared polygenic architecture and does not, by itself, encode causal or temporal direction. The added value of LAVA over LDSC is mechanistic: it identifies the specific genomic loci at which the shared architecture is concentrated, including the 5q31.1 cytokine cluster, the *GATA3* region, the 17q12-21 locus, *TSLP*, and *IL1RL1/IL18R1/IL18RAP*. The conceptual separation between symmetric genetic correlation findings and the temporally ordered RWD findings that you highlighted, applies equally to LDSC and LAVA throughout the revised manuscript.

Several corresponding changes were made throughout the manuscript and have been highlighted using the Word-tracking mode:

- Abstract, changed “Genetic correlation analyses supported shared heritability among many disease pairs and integration with RWD allowed to infer directionality.” → “Genetic correlation analyses supported shared heritability among many disease pairs, complemented by the temporal ordering of associations observed in the RWD.”
- Results: changed “This allows to provide a direction of the association of the two diseases when combining genetic correlation with retrospective cohort studies.” → “In these pairs, the RWD identified a significant temporal association in one direction only, while the symmetric genetic correlation indicated shared heritability; together, these results identify disease pairs in which a genetically correlated trait pair is accompanied by a clinically observed temporal sequence, without implying causality.”
- Results: changed “In several instances, the combination of RWD and genetic correlation enabled directional interpretation of the associations.” → “In several instances, the temporal ordering observed in the RWD was accompanied by a significant shared polygenic architecture, supporting the biological plausibility of the clinical sequence.”
- Legend to figure 5: changed “Blue indicates concordant associations where both HR and rg are significant and directionally consistent; pink indicates discordant associations; brown indicates that directionality is provided by real-world data; and white indicates that genetic correlation analysis was not performed due to lack of GWAS of well-defined cohorts.” → “Blue indicates pairs with both a significant HR and a significant

rg, representing a clinically observed temporal sequence accompanied by shared heritability; brown indicates pairs in which a directional association is supported by RWD only, with no significant rg detected; pink indicates discordant pairs, in which a significant rg was observed in the absence of a significant HR in either direction, or vice versa; white indicates that genetic correlation analysis was not performed due to the lack of sufficiently powered GWAS."

- Several changes of the causal language in the headings in the results section, i.e., "Chronic sinusitis is the core driver disease within the T2ID network" → "Chronic sinusitis is a central node in the T2ID comorbidity network"

2. In lines 169–170 "self-reported White ethnicity (binary)", race/ethnicity adjustment in the propensity score model appears limited to a binary "White vs. non-White" variable. For a heterogeneous US-based cohort, this specification is likely insufficient and may introduce residual confounding, particularly given that subgroup-specific risks are later reported. The covariate structure should be reconsidered or explicitly acknowledged as a limitation.

We thank the Reviewer for this important methodological observation. We would like to clarify the rationale for the covariate structure and explain why we believe the current design, in combination with the stratified subgroup analyses, addresses this concern more effectively than a more granular multi-category race covariate in the primary propensity-score model.

Self-reported White ethnicity was included as a binary covariate in the matching matrix because White individuals represent the largest racial/ethnic group in the US TriNetX network and in each of the T2ID cohorts (see baseline characteristics in Table 1 and Supplement Tables 2–10). Including this indicator ensures that exposed and unexposed cohorts are balanced on the dominant demographic axis, which is a principled choice for propensity-score matching when the goal is to create comparable primary cohorts rather than to model effect modification by race.

To explicitly address potential racial and ethnic heterogeneity, which you correctly identified as a source of residual confounding in pooled analyses, we performed pre-specified stratified subgroup analyses for Black or African American, Hispanic or Latino, and White patients separately (Methods; Supplement Tables 11–19). These subgroup analyses were defined in the analytical framework prior to data extraction, together with the age and sex strata. We believe that this design choice is methodologically preferable to pooled adjustment with a multi-category race covariate, because stratification surfaces group-specific effect sizes rather than averaging them out under an assumption of exchangeability.

The obtained results support this rationale: Cross-disease risks varied substantially by ancestry in ways that would have been collapsed into a single adjusted HR by a pooled multi-category model. For example, the risk of AD following PN ranged approximately 2.5-fold across strata - from HR 4.17 in Hispanic or Latino, to HR 8.26 in White, to HR 10.26 in Black or African American patients. The risk of EoE following food allergy ranged from HR 6.10 (White) to HR 7.17 (Hispanic or Latino) to HR 8.67 (Black or African American), and the risk of PN following CS ranged approximately 1.8-fold, from HR 1.45 (Hispanic or Latino) to HR 2.38 (White) to HR 2.60 (Black or African American). These differences are large, reproducible across sensitivity analyses, and clinically meaningful - they illustrate that the stratified design surfaces heterogeneity that a pooled adjustment would obscure. We have revised the Results accordingly to make these cross-strata HR ranges more prominent and have added a sentence to the Discussion that ties the observed magnitude of heterogeneity back to the rationale for stratifying rather than pooling.

We have additionally revised the Methods to make this rationale explicit, consistent with the Editor's invitation to expand the justification for the study design and analytical parameters. Specifically, the Covariates subsection now includes the following clarification:

"Self-reported White ethnicity was entered as a binary covariate in the propensity-score matching matrix because White individuals constituted the dominant demographic stratum in each T2ID and control cohort; this specification ensures balance on the principal demographic axis of the source population. Racial and ethnic heterogeneity was addressed separately and more directly through pre-specified subgroup analyses stratified by Black or African American, Hispanic or Latino, and White ancestry, which allow group-specific effect sizes to be estimated without imposing exchangeability across strata."

To further probe residual confounding from healthcare engagement (which may correlate with self-reported ancestry in the US healthcare system), we also performed a new active comparator sensitivity analysis (S4) using incident hypothyroidism, in which all cross-T2ID associations significant at the Bonferroni threshold in the primary analysis remained consistent.

Finally, we have added a sentence to the Limitations paragraph acknowledging that self-reported race and ethnicity are imperfect proxies for genetic ancestry, socioeconomic circumstances, and healthcare access, and that residual confounding from these constructs cannot be fully excluded.

3. In line 189 “The subgroup analyses were stratified by age (1-12 years, >18 years)”, age subgroups are described as “1–12 years” and “>18 years,” with no mention of adolescents aged 13–17. This omission requires clarification, as adolescence is clinically relevant for several T2IDs.

We thank the Reviewer for highlighting this gap. We have added an adolescent age subgroup (13–17 years) using the same propensity-score-matched framework as the existing childhood and adult subgroups. Six of seven evaluable cross-T2ID outcomes following CS, specifically AD, food allergy, asthma, VMR/AR, EoE, and PN, showed lower hazard ratios in adolescence (mean HR 1.62) than in childhood (2.54) or adulthood (2.59). This pattern is consistent with established clinical observations of partial T2ID remission during adolescence. COPD showed an opposite age pattern (childhood 3.98, adolescent 3.75, adult 1.58), which may reflect either ICD-10 J44 coding heterogeneity in pediatric and adolescent strata or a distinct biological process; we have addressed this in the Discussion. BP was not evaluable in the adolescent stratum due to insufficient events.

4. In lines 207–208 “Genome-wide association summary statistics for seven major T2IDs (AD, asthma, PN, EoE, CS, COPD, AR and BP)”, the text refers to “seven” diseases while listing eight traits. This discrepancy should be corrected.

Thanks – we corrected to “eight” in the text.

5. Lines 226–228 “We applied a Bonferroni correction for multiple testing to define significance thresholds. Pairwise correlations with FDR-adjusted p-values < 0.05 were considered statistically significant.” reference both Bonferroni correction and FDR-adjusted p-values as criteria for significance. These represent different multiple-testing frameworks and should not be used interchangeably. The statistical correction strategy must be clearly specified and applied consistently.

We thank the Reviewer for identifying this inconsistency, which reflected unclear writing rather than inconsistency in the underlying analyses. The two multiple-testing frameworks in this paper apply to analyses with structurally different testing dimensions and were chosen appropriately for each.

For the propensity-score-matched retrospective cohort analyses, the analytical framework, including the definition of cohorts, matching factors, and outcomes, was defined prior to data collection. A Bonferroni correction was specified at this stage across the eight cross-T2ID outcomes per exposure cohort, with significance defined as $p < 0.0063$ (0.05/8). We

have retained this framework in the revision to preserve the integrity of the pre-defined analytical plan.<br><br>For the local genetic correlation analyses by LAVA (29,789 bivariate tests across 2,485 genomic loci), false discovery rate (FDR) control by the Benjamini–Hochberg procedure was applied with FDR < 0.05 considered statistically significant - the standard approach for high-dimensional genome-wide screening.<br><br>The original Methods text inadvertently referred to both Bonferroni and FDR in the same passage for the genetic correlation analyses, which understandably read as inconsistent. The Methods have been revised to specify each correction strategy clearly and to explain the rationale for the different approaches across analyses.
6. Overall, clarification of inferential boundaries and statistical consistency is necessary to strengthen the manuscript.
We believe the specific concerns raised in Comments 1–5 have all been addressed in the revised manuscript: Specifically: (1) The directionality language has been audited and revised throughout. (2) The rationale for the propensity-score covariate matrix and the use of stratified subgroup analyses for racial and ethnic heterogeneity has been expanded. (3) A third age stratum (adolescents 13–17 years) has been added with corresponding analyses. (4) The count of T2IDs has been corrected and is now consistent throughout. (5) The multiple-testing frameworks have been clarified and made internally consistent for each analysis (Bonferroni for the pre-defined cohort analyses; Benjamini–Hochberg FDR for the high-dimensional LAVA local genetic correlation analyses); the rationale for the different approaches is now explicit in the Methods. We hope the revised manuscript now meets the Reviewer's expectations on these points; we welcome any further specific concerns the Reviewer may identify on re-review.

**Reviewer #2**
1. The manuscript repeatedly interprets asymmetric HRs as evidence for directional influence. This interpretation is not fully supported through the provided analytical framework, since HR asymmetry can arise from multiple non-causal mechanisms. Despite this is properly acknowledged at lines 425-428, stronger causal or upstream framing persists elsewhere, as for example at lines 246-247, 355-357 and 401-402. I recommend moderating causal language and reframing these findings as directional associations rather than upstream disease effects.
Thank you for pointing this out. Please also see our answer to a similar question of Reviewer 1 (question 1). Regarding the specific passages, these were changed:<br>- Lines 246-247 “Across the disease progression network, chronic sinusitis emerged as the core driver disease risk within T2IDs.” → “In the primary analysis, incident CS was significantly associated with an increased risk of seven of the eight investigated T2IDs”<br>- Lines 355-357 “This directional asymmetry suggests that the shared genetic architecture between CS and asthma predominantly contributes to asthma development in individuals with CS, rather than vice versa.” → “The temporal ordering observed in the RWD, in the presence of a strong shared genetic architecture, is consistent with a clinical sequence in which CS typically precedes asthma, though causal inference cannot be made from these data.<br>- Lines 401-402 “In a few cases, only one clinical direction was significant, but genetic correlation provided evidence of shared architecture, enabling directional inference.” → “In a few cases, only one clinical direction was significant, but genetic correlation provided evidence of shared architecture, enabling inference of the temporal sequence observed clinically, while shared heritability remains symmetric.”<br>- Lines 425-428 “Although temporal sequencing in RWD and concordant genetic correlations increase the biological plausibility of certain disease transitions (e.g., CS to asthma), genetic correlation reflects shared heritability rather than direct causation” → “Although temporal sequencing in RWD and concordant genetic correlations increase the biological plausibility of certain clinical sequences (e.g., CS preceding asthma), genetic correlation reflects shared heritability and does not, by itself, indicate causal or directional relationships.”

2. It is well-known that patients with chronic inflammatory diseases have higher healthcare engagement (PMID: 31906907, 40266617, 32266326), thus increasing diagnostic probability of additional conditions independent of biological risk. The propensity score model does not appear to control for any similar variable such as clinical encounter and diagnostic testing frequency among others. Since I suspect that such data are absent in the available dataset, the authors should include negative control outcomes like age-related cataract and non-atopic conditions (e.g., acne vulgaris, varicose veins) and/or negative control exposures like vaccination history and screening procedures to probe confounding by healthcare engagement and identify detection bias.
To address these valid concerns, we have now implemented two complementary robustness analyses that together probe for both comparator-induced bias and residual confounding from unmeasured factors:<br><br>1. Active comparator sensitivity analysis (S4). Patients with incident hypothyroidism (E03.2 / .3 / .8 / .9) were selected as an active comparator cohort, because hypothyroidism requires regular clinical encounters, laboratory monitoring, and chronic disease management, but has no established pathophysiological link to type 2 inflammation. Using this comparator in place of the Z00-based control cohort better equalizes the baseline healthcare engagement profile between exposed and unexposed cohorts and directly probes the reviewer's concern. For each of the T2ID index cohorts, the primary analysis was re-run with hypothyroidism as the comparator, applying the same covariate matrix and time windows. Results of this sensitivity analysis are shown in Supplement Tables 11-19 and described in the Results. In conclusion, across all nine T2ID index cohorts, all cross-T2ID associations significant in the primary and S1–S3 analyses remained consistent under S4. This new data has been integrated in Figure 3. This confirms the robustness of the primary findings against differential healthcare engagement.<br><br>2. To probe residual confounding from factors not captured in the covariate matrix, we also performed pre-specified negative control outcome analyses. Fracture of clavicle (ICD-10-CM: S42.0) was selected as the negative control outcome. For each of the T2ID index cohorts, we estimated the risk of clavicular fracture using the same PSM design and time windows as the primary analysis. Results were integrated into Supplement Tables 11-18. Across the T2ID index cohorts, negative control HRs were broadly null or near-null in seven cohorts (AD, AR, food allergy, EoE, CS, BP, asthma; HRs 0.55–1.07), supporting the interpretation that the primary cross-T2ID findings are not explained by residual confounding. A modest signal was observed for COPD (HR 1.21), and a more substantial signal for PN (HR 1.77), indicating that residual confounding contributes to the cross-T2ID risks observed in these two cohorts. Importantly, even in PN and COPD the negative control

HRs are substantially smaller than the cross-T2ID HRs reported in the primary analysis (typically 2- to 10-fold larger), indicating that residual confounding cannot account for the observed cross-disease findings. We have added a corresponding sentence to the Limitations paragraph acknowledging that residual confounding cannot be entirely excluded, particularly for the PN cohort.

Taken together, these two complementary analyses provide robust reassurance that the cross-T2ID associations reported in the primary analysis are not principally explained by either differential healthcare engagement (probed by the active comparator) or unmeasured residual confounding (probed by the negative control outcomes). Corresponding Methods, Results, and Limitations text has been added to the revised manuscript.

3. The manuscript states that LD score regression was used to estimate heritability but does not report SNP-based heritability estimates or LDSC intercepts. Reporting these metrics, preferably in Table 2 on the liability scale, would improve transparency and interpretability of the genetic analyses.

We thank you for this important suggestion to improve the transparency and interpretability of our genetic analyses. We have added SNP-based heritability estimates and LDSC intercepts on the liability scale, reported in a new Table 3.

Liability-scale h^2 estimates ranged from 0.046 (SE = 0.003) for asthma to 0.201 (SE = 0.068) for prurigo nodularis, indicating that common genetic variants explain approximately 5–20% of disease liability across the type 2 inflammatory diseases studied. The estimates for prurigo nodularis and bullous pemphigoid had relatively large standard errors, reflecting the smaller case counts available for these conditions (n = 606 and n = 507, respectively), and are interpreted with corresponding caution.

LDSC intercepts ranged from 0.788 to 1.210. The asthma intercept (0.788) was below 1.0, which can occur in large, well-powered GWAS meta-analyses and does not indicate confounding; the remaining intercepts were close to 1.0 (0.94–1.21), consistent with minimal genomic inflation from population stratification or cryptic relatedness. Genomic control lambda (λGC) values ranged from 1.014 to 1.287, consistent with polygenic architectures.

Because these per-trait LDSC heritability and quality-control metrics are conceptually distinct from the per-dataset GWAS provenance information in Table 2 (which lists multiple source datasets per trait for the meta-analyzed phenotypes), we present the heritability metrics in a dedicated Table 3 for clarity, with all estimates on the liability scale as requested. The estimation procedure has been added to the Methods (Genetic correlation studies), and the metrics are reported in Table 3.

4. There are multiple genetic-based evidence that could enhance the interpretation of the epidemiological findings of the present manuscript. Nevertheless, most of them are out of scope of the current work. However, since the authors do claim about shared genetic architecture, I would advise them to run local genetic correlations to investigate whether specific loci are consistently associated with all T2IDs. If, for example, the cytokine cluster at the 5q31-33 region shows significant genetic correlations, this would provide some mechanistic evidence about the findings.
We thank you for this excellent and constructive suggestion, which we have implemented in the revised version of the manuscript. We performed local genetic correlation analyses using LAVA to localize the shared genetic signal underlying the T2IDs at the resolution of individual genomic loci, complementing the genome-wide average estimates from LDSC.<br><br>The analysis was performed for the seven T2IDs with sufficiently powered GWAS summary statistics (AD, asthma, COPD, VMR/AR, CS, EoE, and chronic urticaria). PN and BP were excluded because their GWAS case counts (PN n=606; BP n=507) were too small for reliable estimation of local heritability at individual loci. Chronic urticaria was included as an additional T2ID for which suitable GWAS summary statistics were available, although it was not analyzed in the retrospective cohort studies. Across 2,485 LD-independent genomic blocks and 21 disease pairs (29,789 bivariate tests), 1,310 bivariate associations reached statistical significance at FDR < 0.05 (Benjamini–Hochberg), distributed across 852 distinct loci. The shared local genetic signal was predominantly concordant (1,163 of 1,310 significant correlations, 89%, were positive; median local rg=0.61).<br><br>Directly addressing your interest in the 5q31-33 region, the 5q31.1 cytokine gene cluster (chr5:130.6–132.6 Mb; *IL4, IL5, IL13, CSF2, RAD50, IRF1*) showed eight FDR-significant, uniformly positive local genetic correlations, with rg values ranging from 0.40 (asthma–CS) to 0.74 (asthma–COPD), including asthma–AD (rg=0.70, $P=1.3\times10^{-29}$) and VMR/AR–asthma (rg=0.64, $P=1.5\times10^{-15}$).<br><br>Addressing your question of whether specific loci are consistently associated across the T2IDs: the *GATA3* region (10p14) and the 17q12-21 locus (*ORMDL3/GSDMB/IKZF3*) reached significance for all seven diseases tested. The shared signal more broadly converged on canonical components of the type 2 inflammatory pathway, including the alarmin genes *TSLP* (5q22) and *IL1RL1/IL18R1/IL18RAP* (2q12, encoding the IL-33 receptor *ST2*), the Th2 lineage regulators *STAT6* (12q13.3) and *GATA3*, and the 5q31.1 effector cytokine cluster. This convergence provides locus-level mechanistic support for the shared polygenic architecture inferred from the global LDSC analyses.

Asthma was involved in the largest number of shared loci with every other T2ID examined (asthma–COPD, 400 loci; asthma–CS, 108; asthma–VMR/AR, 101; asthma–AD, 93), positioning it as the central node of the shared polygenic architecture; this complements rather than contradicts the central position of CS in the temporal clinical network, as the two findings describe different layers of the network (shared polygenic susceptibility versus clinical temporal ordering).<br><br>These analyses are now reported in the Methods (Local genetic correlation), the Results (new section: 'Local genetic correlation identifies shared mechanistic loci and positions asthma as the central node of the polygenic architecture'), Figure 6, and Supplementary Dataset 5. We are grateful to the Reviewer for prompting this valuable addition, which materially strengthens the mechanistic interpretation of the epidemiological findings.
5. While the authors appropriately note limited power for Mendelian randomization due to GWAS sample size constraints at lines 424-425, the manuscript's central claims regarding directionality would benefit from causal inference analyses for at least the most central results. This is even more prominent at lines 104-106, where the authors acknowledge the lack of published data for T2ID combinations. Even exploratory Mendelian randomization analyses would substantially strengthen the interpretation of directional relationships. I do acknowledge that some stratified analysis findings cannot be validated without individual-level genetic data.
We thank you for this comment. We have addressed it in three ways. *First*, following the directionality concerns of you and R1Q1 we have removed causal language throughout; directional statements are now restricted to the temporal ordering observed in the RWD, and genetic correlations are interpreted strictly as symmetric shared heritability. The claims as written therefore do not require causal inference.<br><br>*Second*, the causal directionality of the central relationships in our network has been examined by published bidirectional MR. For CS and asthma, Zhang et al. (PMID: 36585990) reported a bidirectional causal relationship, including a causal effect of CS on asthma (OR 1.08) concordant with the temporal sequence we observe. For CS and AD, a bidirectional MR identified the predominant genetic causal direction as AD→CS, with no significant reverse effect (Luo et al. PMID: 41806534) - a direction that differs from the temporal sequence captured in our healthcare data. This divergence illustrates the central methodological distinction of our study: genetic causal direction and the clinical temporal order observed in EHR data are conceptually different constructs, and we interpret our RWD findings strictly as temporal associations rather than causal effects. For the remaining, rarer

associations (CS with COPD, EoE, PN, BP, and food allergy), MR has not been reported and is currently precluded by GWAS power for the rare phenotypes.<br>*Third*, additional MR within our own panel would be partly redundant with this work and methodologically compromised: our local genetic correlation analyses (Figure 6) show extensive shared polygenic architecture (89% of significant local correlations positive), which violates the exclusion-restriction assumption of MR. We have therefore retained the non-causal framing and noted in the Discussion that comprehensive causal inference will require larger, ancestry-diverse GWAS and pleiotropy-robust methods.
6. At lines 226-227, the authors state using Bonferroni correction for LDSC results, while at lines 227-228 the significance of pairwise genetic correlation estimates is defined as FDR-adjusted p-values < 0.05. Finally, at lines 228-229, "the presence of a significant genetic correlation (rg>0) was interpreted as evidence of shared heritability between traits". It is not clear what the significance threshold of LDSC results was.
We thank you for identifying this inconsistency. The same point was raised by Reviewer #1 (Q5). The global LDSC genetic correlation analyses used a Bonferroni correction across the 28 unique trait pairs, with $p < 0.0018$ (0.05/28) considered statistically significant. This threshold was pre-specified. The contradictory reference to FDR-adjusted p-values in the original Methods was an error: false discovery rate (Benjamini–Hochberg) control was applied only to the local genetic correlation analyses by LAVA, which involve a much higher-dimensional testing space (29,789 bivariate tests across 2,485 loci) for which FDR is the appropriate and standard framework. We have revised the Methods to state each threshold unambiguously and to remove the erroneous cross-reference.
7. The authors could in fact visualize the genetic correlation estimates using LDSC networks (e.g., https://github.com/mkanai/ldsc-network-plot) and combine with HR estimates to improve clarity of Figure 5, although consistency between Figure 2, Figure 4 and Figure 5 may be more important for the authors.
Thank you for pointing us towards this interesting visualization. We would, however, like to keep consistency amongst the figures.
8. I appreciate the authors' effort to provide colorblind-friendly images, and especially Figure 5. However, the white letters at Figure 5, concordant associations are not that much visible; kindly recolor them to black.
Thanks – changed as suggested.

**Reviewer #3**
1. Line 105: Abbreviations should be spelled out
Thanks – all abbreviations are “introduced” in the first paragraph or the introduction. In addition, we now spell out AR in line 88.
2. Line 164: Control definition should include excluding those with the atopic disease<br>Line 174: shouldn't family history be considered a covariate?
Line 164: Thanks for pointing this out! This was done (see Supplement Table 1), but indeed not mentioned here. We added the missing information. By stating “Same exclusions were applied as for cases.”<br>Line 174: We are in full agreement with the reviewer. We, however, decided not to include the following codes because they are not consistently recorded and, in most cases, unspecific:<br>- Z82.5 (Family history of asthma and other chronic lower respiratory diseases), <0.5% in cases and controls<br>- Z83.6 (Family history of other diseases of the respiratory system), <0.5% in cases and controls<br>- Z84.0 (Family history of diseases of the skin and subcutaneous tissue), <0.5% in cases and controls3<br>In addition, inclusion of these would most likely have been challenging for comparisons between cases and controls with a high total n because the number of covariates on the TriNetX platform has computational constraints.
3. Line 245: could the finding of chronic sinusitis be related to age of the patients? This is commonly.
We thank the Reviewer for this well-placed question. The mean age at CS index (42.7 ± 21.8 years) sits in the middle of the age distribution across the nine T2IDs studied, with three T2IDs having substantially older index populations (BP 74.3 years, COPD 67.3 years, PN 56.4 years) and five having younger ones (food allergy 8.5 years, AD 16.3 years, EoE 18.6 years, asthma 20.6 years, AR/VMR 37.3 years). The central network position of CS is therefore not attributable to either an unusually old or an unusually young index population, as CS does not occupy an extreme of the age distribution. This is further supported by our pre-specified age-stratified subgroup analyses: incident CS was significantly associated with downstream T2IDs in both the childhood (1–12 years) and adult (≥18 years) strata, with HRs of comparable magnitude across age groups (e.g., asthma HR 3.24 in children vs. 2.95 in adults; AD HR 2.85 vs. 2.32; AR/VMR HR 3.42 vs. 3.93). The persistence of CS-driven cross-T2ID risk across both younger and older age strata indicates that the central position

of CS in the network reflects a biological feature of the disease rather than age-related characteristics of the cohort. We have added a brief clarifying sentence to the Discussion to make this point explicit for readers who may share this notion (first paragraph of the discussion): “The mean age at CS index (42.7 years) lies in the middle of the T2ID age distribution, and CS-associated cross-T2ID risks persist across both childhood and adult age strata, indicating that the central network position of CS is not attributable to age-related characteristics of the cohort.”
