# Supplementary material for: Trajectory and genetic correlation mapping of cross-disease risks following incident type 2 inflammatory diseases
Source: Commun Med (Lond). 2026 Nov 6;6:578. doi: 10.1038/s43856-026-02000-y (PMC13743556; doi:10.1038/s43856-026-02000-y)
Supplement: Supplementary file 2 [file 43856_2026_2000_MOESM2_ESM.pdf]

Supplementary Information

Trajectory and genetic correlation mapping of cross-disease risks following incident type 2 inflammatory diseases

Gupta, Olbrich, Thaci, Bieber, Curman & Ludwig

Supplementary Table 1

Cohort/Disease description	Inclusion: ICD-10-CM (description)	Exclusion: ICD-10-CM (description)
CS	J33 ("Nasal polyp"), J32 ("Chronic sinusitis")	J30 ("Vasomotor and allergic rhinitis"), L20 ("Atopic dermatitis"), L28.1 ("Prurigo nodularis"), Z91.01 ("Food allergy status"), J44 ("Other chronic obstructive pulmonary disease"), J45 ("Asthma"), T78.0 ("Anaphylactic reaction due to food"), L12.0 ("Bullous pemphigoid"), L14 ("Bullous disorders in diseases classified elsewhere"), L12.9 ("Pemphigoid, unspecified"), L13 ("Other bullous disorders"), L12.3 ("Acquired epidermolysis bullosa"), L12.2 ("Chronic bullous disease of childhood"), L12.1 ("Cicatricial pemphigoid"), L12.8 ("Other pemphigoid"), L10 ("Pemphigus")
AD	L20 ("Atopic dermatitis")	J30 ("Vasomotor and allergic rhinitis"), L28.1 ("Prurigo nodularis"), K20.0 ("Eosinophilic esophagitis"), J33 ("Nasal polyp"), J32 ("Chronic sinusitis"), Z91.01 ("Food allergy status"), J44 ("Other chronic obstructive pulmonary disease"), J45 ("Asthma"), T78.0 ("Anaphylactic reaction due to food"), L12.0 ("Bullous pemphigoid"), L14 ("Bullous disorders in diseases classified elsewhere"), L12.9 ("Pemphigoid, unspecified"), L13 ("Other bullous disorders"), L12.3 ("Acquired epidermolysis bullosa"), L12.2 ("Chronic bullous disease of childhood"), L12.1 ("Cicatricial pemphigoid"), L12.8 ("Other pemphigoid"), L10 ("Pemphigus")
Food allergy	Z91.01 ("Food allergy status"), T78.0 ("Anaphylactic reaction due to food")	J30 ("Vasomotor and allergic rhinitis"), L20 ("Atopic dermatitis"), L28.1 ("Prurigo nodularis"), K20.0 ("Eosinophilic esophagitis"), J33 ("Nasal polyp"), J32 ("Chronic sinusitis"), J44 ("Other chronic obstructive pulmonary disease"), J45 ("Asthma"), L12.0 ("Bullous pemphigoid"), L14 ("Bullous disorders in diseases classified elsewhere"), L12.9 ("Pemphigoid, unspecified"), L13 ("Other bullous disorders"), L12.3 ("Acquired epidermolysis bullosa"), L12.2 ("Chronic bullous disease of childhood"), L12.1 ("Cicatricial pemphigoid"), L12.8 ("Other pemphigoid"), L10 ("Pemphigus")
VMR/AR	J30 ("Vasomotor and allergic rhinitis")	L20 ("Atopic dermatitis"), L28.1 ("Prurigo nodularis"), K20.0 ("Eosinophilic esophagitis"), J33 ("Nasal polyp"), J32 ("Chronic sinusitis"), Z91.01 ("Food allergy status"), J44 ("Other chronic obstructive pulmonary disease"), J45 ("Asthma"), T78.0 ("Anaphylactic reaction due to food"), L12.0 ("Bullous pemphigoid"), L14 ("Bullous disorders in diseases classified elsewhere"), L12.9 ("Pemphigoid, unspecified"), L13 ("Other bullous disorders"), L12.3 ("Acquired epidermolysis bullosa"), L12.2 ("Chronic bullous disease of childhood"), L12.1 ("Cicatricial pemphigoid"), L12.8 ("Other pemphigoid"), L10 ("Pemphigus")
PN	L28.1 ("Prurigo nodularis")	J30 ("Vasomotor and allergic rhinitis"), L20 ("Atopic dermatitis"), K20.0 ("Eosinophilic esophagitis"), J33 ("Nasal polyp"), J32 ("Chronic sinusitis"), Z91.01 ("Food allergy status"), J44 ("Other chronic obstructive pulmonary disease"), J45 ("Asthma"), T78.0 ("Anaphylactic reaction due to food"), L12.0 ("Bullous pemphigoid"), L14 ("Bullous disorders in diseases classified elsewhere"), L12.9 ("Pemphigoid, unspecified"), L13 ("Other bullous disorders"), L12.3 ("Acquired epidermolysis bullosa"), L12.2 ("Chronic bullous disease of childhood"), L12.1 ("Cicatricial pemphigoid"), L12.8 ("Other pemphigoid"), L10 ("Pemphigus")
Asthma	J45 ("Asthma")	J30 ("Vasomotor and allergic rhinitis"), L20 ("Atopic dermatitis"), L28.1 ("Prurigo nodularis"), K20.0 ("Eosinophilic esophagitis"), J33 ("Nasal polyp"), J32 ("Chronic sinusitis"), Z91.01 ("Food allergy status"), J44 ("Other chronic obstructive pulmonary disease"), T78.0 ("Anaphylactic reaction due to food"), L12.0 ("Bullous pemphigoid"), L14 ("Bullous disorders in diseases classified elsewhere"), L12.9 ("Pemphigoid, unspecified"), L13 ("Other bullous disorders"), L12.3 ("Acquired epidermolysis bullosa"), L12.2 ("Chronic bullous disease of childhood"), L12.1 ("Cicatricial pemphigoid"), L12.8 ("Other pemphigoid"), L10 ("Pemphigus")
EoE	K20.0 ("Eosinophilic esophagitis")	J30 ("Vasomotor and allergic rhinitis"), L20 ("Atopic dermatitis"), L28.1 ("Prurigo nodularis"), J33 ("Nasal polyp"), J32 ("Chronic sinusitis"), Z91.01 ("Food allergy status"), J44 ("Other chronic obstructive pulmonary disease"), J45 ("Asthma"), T78.0 ("Anaphylactic reaction due to food"), L12.0 ("Bullous pemphigoid"), L14 ("Bullous disorders in diseases classified elsewhere"), L12.9 ("Pemphigoid, unspecified"), L13 ("Other bullous disorders"), L12.3 ("Acquired epidermolysis bullosa"), L12.2 ("Chronic bullous disease of childhood"), L12.1 ("Cicatricial pemphigoid"), L12.8 ("Other pemphigoid"), L10 ("Pemphigus")
COPD	J44 ("Other chronic obstructive pulmonary disease")	J30 ("Vasomotor and allergic rhinitis"), L20 ("Atopic dermatitis"), L28.1 ("Prurigo nodularis"), K20.0 ("Eosinophilic esophagitis"), J33 ("Nasal polyp"), J32 ("Chronic sinusitis"), Z91.01 ("Food allergy status"), J45 ("Asthma"), T78.0 ("Anaphylactic reaction due to food"), L12.0 ("Bullous pemphigoid"), L14 ("Bullous disorders in diseases classified elsewhere"), L12.9 ("Pemphigoid, unspecified"), L13 ("Other bullous disorders"), L12.3 ("Acquired epidermolysis bullosa"), L12.2 ("Chronic bullous disease of childhood"), L12.1 ("Cicatricial pemphigoid"), L12.8 ("Other pemphigoid"), L10 ("Pemphigus")

Cohort/Disease description	Inclusion: ICD-10-CM (description)	Exclusion: ICD-10-CM (description)
BP	L12.0 ("Bullous pemphigoid")	J30 ("Vasomotor and allergic rhinitis"), L20 ("Atopic dermatitis"), L28.1 ("Prurigo nodularis"), K20.0 ("Eosinophilic esophagitis"), J33 ("Nasal polyp"), J32 ("Chronic sinusitis"), Z91.01 ("Food allergy status"), J44 ("Other chronic obstructive pulmonary disease"), J45 ("Asthma"), T78.0 ("Anaphylactic reaction due to food"), L14 ("Bullous disorders in diseases classified elsewhere"), L12.9 ("Pemphigoid, unspecified"), L13 ("Other bullous disorders"), L12.3 ("Acquired epidermolysis bullosa"), L12.2 ("Chronic bullous disease of childhood"), L12.1 ("Cicatricial pemphigoid"), L12.8 ("Other pemphigoid"), L10 ("Pemphigus")
Non-T2ID controls	Z00 ("Encounter for general examination without complaint, suspected or reported diagnosis")	J30 ("Vasomotor and allergic rhinitis"), L20 ("Atopic dermatitis"), L28.1 ("Prurigo nodularis"), K20.0 ("Eosinophilic esophagitis"), J33 ("Nasal polyp"), J32 ("Chronic sinusitis"), Z91.01 ("Food allergy status"), J44 ("Other chronic obstructive pulmonary disease"), J45 ("Asthma"), T78.0 ("Anaphylactic reaction due to food"), L12.0 ("Bullous pemphigoid"), L14 ("Bullous disorders in diseases classified elsewhere"), L12.9 ("Pemphigoid, unspecified"), L13 ("Other bullous disorders"), L12.3 ("Acquired epidermolysis bullosa"), L12.2 ("Chronic bullous disease of childhood"), L12.1 ("Cicatricial pemphigoid"), L12.8 ("Other pemphigoid"), L10 ("Pemphigus")

Supplementary Table 2

Summarized baseline characteristics for all study cohorts

Characteristic	Before propensity-score matching			After propensity-score matching		
	Exposed	Unexposed	Std. diff.	Exposed	Unexposed	Std. diff.
Chronic sinusitis						
n	1,778,537	13,421,991	-	1,649,126	1,649,126	-
Age at index (years, SD)	42.7 ± 21.8	35.6 ± 24.6	0.309	42.7 ± 21.8	42.7 ± 21.8	<0.001
Female (%)	55.6	50.1	0.111	55.6	55.6	<0.001
Follow-up time (days)	1,685	1,431	-	1,685	1,491	-
Atopic dermatitis						
n	774,536	13,108,725	-	733,240	733,240	-
Age at index (years, SD)	16.3 ± 22.7	35.5 ± 24.7	0.81	16.3 ± 22.7	16.4 ± 22.7	<0.001
Female (%)	51.2	50	0.022	51.2	51.2	<0.001
Follow-up time (days)	1,000	931	-	1,000	931	-
Food allergy						
n	423,963	13,108,725	-	402,977	402,977	-
Age at index (years, SD)	8.5 ± 14.7	35.5 ± 24.7	1.166	8.5 ± 14.7	8.5 ± 14.7	<0.001
Female (%)	44.9	50	0.102	44.9	44.9	<0.001
Follow-up time (days)	1,052	931	-	1,052	1,045	-
Allergic rhinitis and vasomotor rhinitis						
n	3,487,860	13,421,991	-	3,336,609	3,336,609	-
Age at index (years, SD)	37.3 ± 24.1	35.6 ± 24.6	0.07	37.3 ± 24.1	37.5 ± 24.1	0.009
Female (%)	55.1	50.1	0.101	55.1	55.1	0.002
Follow-up time (days)	1,670	1,431	-	1,669	1,458	-
Prurigo nodularis						
n	81,596	13,108,725	-	76,872	76,872	-
Age at index (years, SD)	56.4 ± 16.6	35.5 ± 24.7	0.967	56.4 ± 16.6	56.4 ± 16.6	<0.001
Female (%)	63.4	50	0.271	63.4	63.3	<0.001
Follow-up time (days)	1,065	931	-	1,065	934	-
Asthma						
n	4,129,468	13,108,725	-	3,831,861	3,831,861	-
Age at index (years, SD)	20.6 ± 22.2	35.5 ± 24.7	0.684	20.6 ± 22.2	20.6 ± 22.2	<0.001
Female (%)	54.1	50	0.082	54.1	54.1	<0.001
Follow-up time (days)	1,112	931	-	1,112	931	-
Eosinophilic esophagitis						
n	185,732	13,108,725	-	176,818	176,818	-
Age at index (years, SD)	18.6 ± 16.4	35.5 ± 24.7	0.795	18.6 ± 16.4	18.6 ± 16.4	<0.001
Female (%)	33.4	50	0.343	33.4	33.4	<0.001
Follow-up time (days)	1,015	931	-	1,015	1,028	-
Chronic obstructive pulmonary disease						
n	2,030,053	13,421,991	-	1,906,021	1,906,021	-
Age at index (years, SD)	67.3 ± 12.7	35.6 ± 24.6	1.619	67.1 ± 12.7	67.5 ± 13.0	0.039
Female (%)	45	50.1	0.103	45.2	45.1	<0.001
Follow-up time (days)	1,056	1,431	-	1,059	1,391	-

Characteristic	Before propensity-score matching			After propensity-score matching		
	Exposed	Unexposed	Std. diff.	Exposed	Unexposed	Std. diff.
Bullous pemphigoid						
n	6,476	13,421,991	-	6,458	6,458	-
Age at index (years, SD)	74.3 ± 14.0	35.6 ± 24.6	1.934	74.3 ± 14.0	74.3 ± 14.0	<0.001
Female (%)	50.3	50.1	0.003	50.3	50.3	<0.001
Follow-up time (days)	722	1,432	-	722	1,215	-

Summarized baseline characteristics for all study cohorts including cohort sizes, age at index, sex, and follow-up times for the primary analysis. Abbreviations: SD, standard deviation; Std. diff., standardized difference.

Supplementary Table 3

Sources of data for genetic correlation analysis

Trait	ICD-10	FinnGen ICD	Dataset	Cases	Controls	Effective sample size	Effective sample size (total)	Total (N)	PMID
Atopic dermatitis	L20	L12_ATOPIC	GCST90244787	60,653	804,329	225,600	225,600	864,982	37,794,016
Prurigo nodularis	L28.1	L12_PRURIGONOD	FinnGen	606	336,589	2,420	2,420	337,195	
Asthma	J45	ALLERG_ASTHMA	GBMI	121,940	1,254,131	444,537	475,384	1,376,071	36,777,996
			GERA	9,209	47,428	30,847		56,637	33,893,285
Eosinophilic esophagitis	K20.0	K11_OESITIS	GCST90027899	1,930	13,634	6,763	15,564	15,564	34,506,852
Chronic sinusitis	J32	J10_CHRONSINUSITIS	FinnGen	16,395	283,342	61,993	74,006	299,737	
			UK Biobank	3,026	398,757	12,013		401,783	
COPD	J44	COPD_MODE	GBMI	58,559	937,358	220,463	220,463	995,917	36,777,996
Allergic rhinitis	J30	ALLERG_RHINITIS	FinnGen	11,009	359,149	42,726	188,210	370,158	
			GCST90038664	27,415	457,183	103,456		484,598	33,959,723
			GERA	13,936	42,701	42,028		56,637	33,893,285
Bullous pemphigoid	L12.0	L12_PEMPHIGOID_BULL	FinnGen	507	375,767	2,025	2,025	376,274	

Table 2. Sources of data for genetic correlation analysis. New meta-analyses are shown in red. Summary statistics from non-FinnGen datasets are available upon request. FinnGen-derived summary statistics were used under data use restrictions and cannot be publicly shared owing to ownership and licensing agreements with the FinnGen consortium. Abbreviations: COPD, chronic obstructive pulmonary disease; GBMI, Global Biobank Meta-Analysis Initiative; GERA, Genetic Epidemiology Research on Adult Health and Aging.

Supplementary Table 4

Baseline characteristics: CS												
**Characteristic**	**Before PSM**						**After PSM**					
	cases		controls		p-value	SMD	cases		controls		p-value	SMD
Age at Index (mean ± SD)	42.741	21.82	35.553	24.637	0	0.3089	42.741	21.82	42.742	21.819	0.9838	2.24e-05
	n =	%	n =	%			n =	%	n =	%		
Total	1,649,126	1	13,058,833	1			1,649,126	1	1,649,126	1		
White	1,152,159	0.6986	7,475,218	0.5724	0	0.2645	1,152,159	0.6986	1,152,149	0.6986	0.9904	1.32e-05
Female	917,562	0.5564	6,541,309	0.5009	0	0.1113	917,562	0.5564	917,567	0.5564	0.9956	6.10e-06
Black or African American	153,333	0.093	1,777,749	0.1361	0	0.1358	153,333	0.093	165,301	0.1002	0	0.0246
Hispanic or Latino	110,618	0.0671	1,449,552	0.111	0	0.1547	110,618	0.0671	156,440	0.0949	0	0.102
Asian	42,461	0.0257	575,755	0.0441	0	0.1	42,461	0.0257	53,236	0.0323	0	0.0389
Overweight and obesity (E66)	152,470	0.0925	710,744	0.0544	0	0.1462	152,470	0.0925	152,463	0.0925	0.9894	1.47e-05
Nicotine dependence (F17)	98,335	0.0596	390,090	0.0299	0	0.1443	98,335	0.0596	98,332	0.0596	0.9944	7.68e-06
Persons with potential health hazards related to socioeconomic and psychosocial circumstances (Z55-65)	24,416	0.0148	141,209	0.0108	0	0.0355	24,416	0.0148	24,412	0.0148	0.9854	2.01e-05

Supplementary Table 5

Baseline characteristics: AD												
**Characteristic**	**Before PSM**						**After PSM**					
	cases		controls		p-value	SMD	cases		controls		p-value	SMD
Age at Index (mean ± SD)	16.333	22.673	35.515	24.675	0	0.8095	16.336	22.674	16.355	22.669	0.6067	0.0009
	n =	%	n =	%			n =	%	n =	%		
Total	733,369	1	12,764,742	1			733,240	1	733,240	1		
White	375,167	0.5116	6,387,892	0.5004	0	0.0223	375,091	0.5115	375,326	0.5119	0.6979	0.0006
Female	323,307	0.4409	7,257,640	0.5686	0	0.2576	323,307	0.4409	323,258	0.4409	0.935	0.0001
Black or African American	155,688	0.2123	1,746,934	0.1369	0	0.1997	155,615	0.2122	139,478	0.1902	0	0.0549
Hispanic or Latino	116,725	0.1592	1,432,841	0.1123	0	0.1373	116,689	0.1591	116,391	0.1587	0.5009	0.0011
Asian	48,448	0.0661	564,827	0.0442	0	0.0957	48,443	0.0661	37,853	0.0516	0	0.0614
Overweight and obesity (E66)	35,167	0.0479	693,427	0.0543	0	0.0289	35,079	0.0478	35,296	0.0481	0.4018	0.0014
Nicotine dependence (F17)	16,111	0.022	137,875	0.0108	0	0.0881	15,982	0.0218	16,469	0.0225	0.0063	0.0045
Persons with potential health hazards related to socioeconomic and psychosocial circumstances (Z55-65)	13,825	0.0188	376,818	0.0295	0	0.0695	13,825	0.0188	13,992	0.0191	0.3121	0.0017

Supplementary Table 6

Baseline characteristics: Food allergy												
**Characteristic**	**Before PSM**						**After PSM**					
	cases		controls		p-value	SMD	cases		controls		p-value	SMD
Age at Index (mean ± SD)	26.829	25.459	35.553	24.637	0	0.3482	26.829	25.459	26.829	25.459	0.9962	1.03e-05
	n =	%	n =	%			n =	%	n =	%		
Total	426,575	1	13,058,833	1			426,574	1	426,574	1		
White	242,837	0.5693	7,475,218	0.5724	4.16e-05	0.0064	242,836	0.5693	242,835	0.5693	0.9983	4.73e-06
Female	233,439	0.5472	6,541,309	0.5009	0	0.0929	233,438	0.5472	233,438	0.5472	1	0
Black or African American	67,219	0.1576	1,777,749	0.1361	0	0.0606	67,219	0.1576	62,739	0.1471	1.61e-41	0.0292
Hispanic or Latino	41,955	0.0984	1,449,552	0.111	0	0.0413	41,955	0.0984	57,804	0.1355	0	0.1158
Asian	24,604	0.0577	575,755	0.0441	0	0.0619	24,604	0.0577	18,122	0.0425	0	0.0697
Overweight and obesity (E66)	30,284	0.071	710,744	0.0544	0	0.0684	30,283	0.071	30,283	0.071	1	0
Nicotine dependence (F17)	18,608	0.0436	390,090	0.0299	0	0.0731	18,607	0.0436	18,606	0.0436	0.9958	1.15e-05
Persons with potential health hazards related to socioeconomic and psychosocial circumstances (Z55-65)	9,683	0.0227	141,209	0.0108	0	0.0927	9,682	0.0227	9,686	0.0227	0.9768	6.30e-05

Supplementary Table 7

Baseline characteristics: VMR/AR											
**Characteristic**	**Before PSM**						**After PSM**				
	cases		controls		p-value	SMD	cases		controls		p-value	SMD
Age at Index (mean ± SD)	37.27	24.102	35.553	24.637	0	0.0705	37.27	24.102	37.485	24.087	8.56e-31	0.0089
	n =	%	n =	%			n =	%	n =	%		
Total	3,336,609	1	13,058,833	1			3,336,609	1	3,336,609	1		
White	2,007,799	0.6018	7,475,218	0.5724	0	0.0596	2,007,799	0.6018	2,007,993	0.6018	0.8781	0.0001
Female	1,839,871	0.5514	6,541,309	0.5009	0	0.1013	1,839,871	0.5514	1,837,266	0.5506	0.0426	0.0016
Black or African American	489,615	0.1467	1,777,749	0.1361	0	0.0304	489,615	0.1467	443,558	0.1329	0	0.0398
Hispanic or Latino	402,864	0.1207	1,449,552	0.111	0	0.0304	402,864	0.1207	357,688	0.1072	0	0.0426
Asian	134,976	0.0404	575,755	0.0441	0	0.0181	134,976	0.0404	136,986	0.0411	8.31e-05	0.003
Overweight and obesity (E66)	323,607	0.097	710,744	0.0544	0	0.1614	323,607	0.097	319,738	0.0958	3.89e-07	0.0039
Nicotine dependence (F17)	120,880	0.0362	390,090	0.0299	0	0.0356	120,880	0.0362	124,214	0.0372	6.81e-12	0.0053
Persons with potential health hazards related to socioeconomic and psychosocial circumstances (Z55-65)	63,357	0.019	141,209	0.0108	0	0.0675	63,357	0.019	65,948	0.0198	3.43e-13	0.0056

Supplementary Table 8

Baseline characteristics: PN												
Characteristic	Before PSM						After PSM					
	cases		controls		p-value	SMD	cases		controls		p-value	SMD
Age at Index (mean ± SD)	54.688	17.847	35.553	24.637	0	0.8895	54.688	17.847	54.688	17.847	1	0
	n =	%	n =	%			n =	%	n =	%		
Total	71,694	1	13,058,833	1			71,694	1	71,694	1		
White	42,555	0.5936	7,475,218	0.5724	3.75e-30	0.0429	42,555	0.5936	42,556	0.5936	0.9957	2.84e-05
Female	36,292	0.5062	6,541,309	0.5009	0.0047	0.0106	36,292	0.5062	36,293	0.5062	0.9958	2.79e-05
Black or African American	8,742	0.1219	1,777,749	0.1361	1.99e-28	0.0424	8,742	0.1219	9,800	0.1367	8.31e-17	0.044
Hispanic or Latino	4,527	0.0631	1,449,552	0.111	0	0.1704	4,527	0.0631	5,062	0.0706	1.55e-08	0.0299
Asian	2,614	0.0365	575,755	0.0441	3.17e-23	0.0388	2,614	0.0365	2,997	0.0418	1.83e-07	0.0276
Overweight and obesity (E66)	9,449	0.1318	710,744	0.0544	0	0.2686	9,449	0.1318	9,450	0.1318	0.9938	4.12e-05
Nicotine dependence (F17)	5,449	0.076	390,090	0.0299	0	0.2071	5,449	0.076	5,449	0.076	1	0
Persons with potential health hazards related to socioeconomic and psychosocial circumstances (Z55-65)	1,598	0.0223	141,209	0.0108	0	0.09	1,598	0.0223	1,597	0.0223	0.9857	9.45e-05

Supplementary Table 9

Baseline characteristics: Asthma												
**Characteristic**	**Before PSM**						**After PSM**					
	cases		controls		p-value	SMD	cases		controls		p-value	SMD
Age at Index (mean ± SD)	34.045	22.875	35.553	24.637	0	0.0635	34.045	22.875	34.092	22.873	0.0029	0.0021
	n =	%	n =	%			n =	%	n =	%		
Total	4,058,716	1	13,058,833	1			4,058,716	1	4,058,716	1		
White	2,319,339	0.5715	6,541,309	0.5009	0	0.1418	2,319,339	0.5715	2,319,699	0.5715	0.7985	0.0002
Female	2,198,633	0.5417	7,475,218	0.5724	0	0.0619	2,198,633	0.5417	2,199,076	0.5418	0.755	0.0002
Black or African American	796,046	0.1961	1,777,749	0.1361	0	0.1617	796,046	0.1961	609,624	0.1502	0	0.1216
Hispanic or Latino	451,290	0.1112	1,449,552	0.111	0.2906	0.0006	451,290	0.1112	450,151	0.1109	0.2032	0.0009
Asian	128,306	0.0316	575,755	0.0441	0	0.0654	128,306	0.0316	198,866	0.049	0	0.0885
Overweight and obesity (E66)	259,292	0.0639	710,744	0.0544	0	0.0401	259,292	0.0639	258,075	0.0636	0.0804	0.0012
Nicotine dependence (F17)	159,859	0.0394	390,090	0.0299	0	0.0521	159,859	0.0394	162,055	0.0399	7.83e-05	0.0028
Persons with potential health hazards related to socioeconomic and psychosocial circumstances (Z55-65)	50,541	0.0124	141,209	0.0108	0	0.0153	50,541	0.0124	51,853	0.0128	3.69e-05	0.0029

Supplementary Table 10

Baseline characteristics: EoE												
**Characteristic**	**Before PSM**						**After PSM**					
	cases		controls		p-value	SMD	cases		controls		p-value	SMD
Age at Index (mean ± SD)	35.377	20.504	35.553	24.637	0.0651	0.0078	35.377	20.504	35.376	20.504	0.9949	3.50e-05
	n =	%	n =	%			n =	%	n =	%		
Total	66,822	1	13,058,833	1			66,822	1	66,822	1		
White	50,830	0.7607	7,475,218	0.5724	0	0.4075	50,830	0.7607	50,829	0.7607	0.9949	3.51e-05
Female	24,228	0.3626	6,541,309	0.5009	0	0.282	24,228	0.3626	24,227	0.3626	0.9955	3.11e-05
Black or African American	3,210	0.048	1,449,552	0.111	0	0.2343	3,210	0.048	7,189	0.1076	0	0.2237
Hispanic or Latino	3,070	0.0459	1,777,749	0.1361	0	0.3175	3,070	0.0459	4,820	0.0721	0	0.1113
Asian	1,067	0.016	575,755	0.0441	0	0.1653	1,067	0.016	1,656	0.0248	3.99e-30	0.0624
Overweight and obesity (E66)	5,250	0.0786	710,744	0.0544	0	0.097	5,250	0.0786	5,250	0.0786	1	0
Nicotine dependence (F17)	2,170	0.0325	390,090	0.0299	8.11e-05	0.015	2,170	0.0325	2,168	0.0324	0.9754	0.0002
Persons with potential health hazards related to socioeconomic and psychosocial circumstances (Z55-65)	925	0.0138	141,209	0.0108	4.44e-14	0.0275	925	0.0138	925	0.0138	1	0

Supplementary Table 11

Baseline characteristics: COPD											
**Characteristic**	**Before PSM**						**After PSM**				
	cases		controls		p-value	SMD	cases		controls		p-value	SMD
Age at Index (mean ± SD)	67.264	12.678	35.553	24.637	0	1.619	67.051	12.702	67.549	13.022	0	0.0387
	n =	%	n =	%			n =	%	n =	%		
Total	1,947,926	1	13,058,833	1			1,906,021	1	1,906,021	1		
White	1,380,944	0.7089	7,475,218	0.5724	0	0.2874	1,346,391	0.7064	1,340,276	0.7032	6.59e-12	0.007
Female	875,704	0.4496	6,541,309	0.5009	0	0.103	861,133	0.4518	860,178	0.4513	0.3257	0.001
Black or African American	192,777	0.099	1,777,749	0.1361	0	0.1156	190,470	0.0999	180,858	0.0949	0	0.017
Hispanic or Latino	60,208	0.0309	1,449,552	0.111	0	0.3158	58,988	0.0309	81,087	0.0425	0	0.0617
Asian	32,484	0.0167	575,755	0.0441	0	0.1602	31,798	0.0167	53,435	0.028	0	0.0768
Overweight and obesity (E66)	290,888	0.1493	390,090	0.0299	0	0.4277	251,852	0.1321	210,232	0.1103	0	0.0669
Nicotine dependence (F17)	151,759	0.0779	710,744	0.0544	0	0.0946	148,129	0.0777	145,458	0.0763	2.88e-07	0.0053
Persons with potential health hazards related to socioeconomic and psychosocial circumstances (Z55-65)	25,073	0.0129	141,209	0.0108	0	0.019	24,278	0.0127	23,516	0.0123	0.0005	0.0036

Supplementary Table 12

Baseline characteristics: BP												
**Characteristic**	**Before PSM**						**After PSM**					
	cases		controls		p-value	SMD	cases		controls		p-value	SMD
Age at Index (mean ± SD)	74.269	13.962	35.553	24.637	0	1.934	74.269	13.962	74.27	13.958	0.997	6.66e-05
	n =	%	n =	%			n =	%	n =	%		
Total	6,458	1	13,058,833	1			6,458	1	6,458	1		
White	4,119	0.6378	7,475,218	0.5724	2.44e-26	0.1341	4,119	0.6378	4,123	0.6384	0.9416	0.0013
Female	3,246	0.5026	6,541,309	0.5009	0.782	0.0034	3,246	0.5026	3,249	0.5031	0.9579	0.0009
Black or African American	586	0.0907	1,777,749	0.1361	2.04e-26	0.1435	586	0.0907	675	0.1045	0.0083	0.0464
Hispanic or Latino	345	0.0534	575,755	0.0441	0.0003	0.0433	345	0.0534	231	0.0358	1.18e-06	0.0856
Asian	213	0.033	1,449,552	0.111	0	0.3053	213	0.033	251	0.0389	0.0724	0.0316
Overweight and obesity (E66)	602	0.0932	710,744	0.0544	6.20e-43	0.1488	602	0.0932	600	0.0929	0.9517	0.0011
Nicotine dependence (F17)	252	0.039	390,090	0.0299	1.58e-05	0.0502	252	0.039	251	0.0389	0.9637	0.0008
Persons with potential health hazards related to socioeconomic and psychosocial circumstances (Z55-65)	98	0.0152	141,209	0.0108	0.0007	0.0385	98	0.0152	99	0.0153	0.9428	0.0013

Supplementary Table 13

KM-Analysis: CS									
Analysis/Strata	Outcome	# (cases)	% (cases)	# (controls)	% (controls)	p-value	HR	HR (95%-CI lower limit)	HR (95%-CI upper limit)
Primary	BP	1,649,126	0.01	1,649,126	0.0057	0.0017	1.496	1.161	1.928
Primary	PN	1,649,126	0.1694	1,649,126	0.0639	< 0.0001	2.34	2.18	2.512
Primary	Asthma	1,649,126	5.831	1,649,126	1.765	< 0.0001	3.118	3.077	3.159
Primary	Food allergy	1,649,126	0.666	1,649,126	0.2701	< 0.0001	2.221	2.145	2.3
Primary	COPD	1,649,126	2.457	1,649,126	1.095	< 0.0001	2.043	2.007	2.079
Primary	VMR/AR	1,649,126	10.245	1,649,126	2.476	< 0.0001	4.052	4.009	4.096
Primary	EoE	1,649,126	0.1526	1,649,126	0.0528	< 0.0001	2.547	2.358	2.751
Primary	AD	1,649,126	0.9053	1,649,126	0.2871	< 0.0001	2.85	2.758	2.945
Primary	Fracture of clavicle	1,544,593	0.448	1,545,907	0.395	0.1292	1.027	0.992	1.063
S1	BP	1,652,818	0.0114	1,652,814	0.0057	< 0.0001	1.704	1.33	2.183
S1	PN	1,652,694	0.1522	1,652,745	0.0511	< 0.0001	2.614	2.417	2.826
S1	Asthma	1,642,267	5.481	1,648,481	1.595	< 0.0001	3.221	3.178	3.266
S1	Food allergy	1,651,931	0.7521	1,652,060	0.2861	< 0.0001	2.34	2.263	2.42
S1	COPD	1,648,884	2.408	1,650,422	1.026	< 0.0001	2.115	2.077	2.154
S1	VMR/AR	1,629,878	9.189	1,648,128	2.118	< 0.0001	4.218	4.169	4.267
S1	EoE	1,652,699	0.1476	1,652,729	0.0535	< 0.0001	2.42	2.24	2.614
S1	AD	1,651,780	0.9222	1,652,331	0.2811	< 0.0001	2.96	2.865	3.059
S2	BP	209,866	0.0172	209,866	0.0081	0.3973	1.283	0.7193	2.289
S2	PN	209,866	0.1963	209,866	0.0872	< 0.0001	1.437	1.207	1.711
S2	Asthma	209,866	5.341	209,866	2.033	< 0.0001	1.763	1.701	1.826
S2	Food allergy	209,866	0.6904	209,866	0.3083	< 0.0001	1.445	1.317	1.586
S2	COPD	209,866	2.382	209,866	1.264	< 0.0001	1.229	1.172	1.288
S2	VMR/AR	209,866	11.147	209,866	2.677	< 0.0001	2.889	2.806	2.975
S2	EoE	209,866	0.1777	209,866	0.0653	< 0.0001	1.736	1.426	2.113
S2	AD	209,866	0.9196	209,866	0.3531	< 0.0001	1.673	1.536	1.821
S3	BP	211,403	0.0161	211,403	0.009	0.548	1.189	0.6756	2.092
S3	PN	211,403	0.1613	211,403	0.0728	< 0.0001	1.493	1.232	1.808
S3	Asthma	211,403	5.092	211,403	2.293	< 0.0001	1.637	1.582	1.694
S3	Food allergy	211,403	0.578	211,403	0.3174	< 0.0001	1.291	1.174	1.419
S3	COPD	211,403	2.296	211,403	1.428	< 0.0001	1.143	1.092	1.197
S3	VMR/AR	211,403	10.638	211,403	2.922	< 0.0001	2.778	2.701	2.858
S3	EoE	211,403	0.1764	211,403	0.0577	< 0.0001	2.078	1.692	2.553
S3	AD	211,403	0.8581	211,403	0.3458	< 0.0001	1.728	1.585	1.884
S4	BP	1,541,196	0.015	1,541,196	0.006	< 0.0001	2.171	1.708	2.76
S4	PN	1,541,196	0.18	1,541,196	0.063	< 0.0001	2.664	2.476	2.867
S4	Asthma	1,541,196	5.979	1,541,196	1.631	< 0.0001	3.598	3.548	3.649
S4	Food allergy	1,541,196	0.83	1,541,196	0.276	< 0.0001	2.809	2.714	2.909
S4	COPD	1,541,196	3.249	1,541,196	1.06	< 0.0001	2.93	2.879	2.982
S4	VMR/AR	1,541,196	10.692	1,541,196	1.993	< 0.0001	5.494	5.428	5.561
S4	EoE	1,541,196	0.152	1,541,196	0.059	< 0.0001	2.376	2.201	2.565
S4	AD	1,541,196	0.949	1,541,196	0.292	< 0.0001	3.041	2.941	3.144
Adult	BP	1,417,729	0.0114	1,417,729	0.0063	0.0003	1.605	1.239	2.08

KM-Analysis: CS									
Analysis/Strata	Outcome	# (cases)	% (cases)	# (controls)	% (controls)	p-value	HR	HR (95%-CI lower limit)	HR (95%-CI upper limit)
Adult	PN	1,417,729	0.2046	1,417,729	0.0791	< 0.0001	2.364	2.207	2.533
Adult	Asthma	1,417,729	5.505	1,417,729	1.803	< 0.0001	2.95	2.909	2.992
Adult	Food allergy	1,417,729	0.6741	1,417,729	0.3061	< 0.0001	2.035	1.963	2.109
Adult	COPD	1,417,729	2.824	1,417,729	1.311	< 0.0001	2.02	1.985	2.056
Adult	VMR/AR	1,417,729	9.719	1,417,729	2.481	< 0.0001	3.933	3.888	3.98
Adult	EoE	1,417,729	0.1578	1,417,729	0.0621	< 0.0001	2.322	2.148	2.51
Adult	AD	1,417,729	0.7127	1,417,729	0.2821	< 0.0001	2.322	2.239	2.409
Adolescent	BP	NA	NA	NA	NA	NA	NA	NA	NA
Adolescent	PN	88,413	0.025	88,413	0.018	0.6176	1.179	0.618	2.25
Adolescent	Asthma	88,413	7.564	88,413	3.366	< 0.0001	2.139	2.048	2.233
Adolescent	Food allergy	88,413	1.197	88,413	0.74	< 0.0001	1.438	1.304	1.585
Adolescent	COPD	88,413	0.224	88,413	0.051	< 0.0001	3.751	2.712	5.187
Adolescent	VMR/AR	88,413	12.102	88,413	5.202	< 0.0001	2.306	2.228	2.387
Adolescent	EoE	88,413	0.206	88,413	0.103	< 0.0001	1.777	1.381	2.287
Adolescent	AD	88,413	1.146	88,413	0.843	< 0.0001	1.228	1.117	1.35
Childhood	BP	163,866	0.0061	163,866	0.0061	0.2915	0.3155	0.0328	3.035
Childhood	PN	163,866	0.0342	163,866	0.0153	0.0075	1.885	1.176	3.023
Childhood	Asthma	163,866	11.046	163,866	3.247	< 0.0001	3.239	3.141	3.34
Childhood	Food allergy	163,866	1.497	163,866	0.678	< 0.0001	1.919	1.788	2.06
Childhood	COPD	163,866	0.1129	163,866	0.0244	< 0.0001	3.975	2.823	5.598
Childhood	VMR/AR	163,866	17.748	163,866	5.189	< 0.0001	3.42	3.339	3.504
Childhood	EoE	163,866	0.2154	163,866	0.0769	< 0.0001	2.336	1.905	2.863
Childhood	AD	163,866	2.926	163,866	0.9258	< 0.0001	2.846	2.686	3.016
Female	BP	991,112	0.0114	991,112	0.0057	0.0007	1.734	1.258	2.391
Female	PN	991,112	0.184	991,112	0.0676	< 0.0001	2.416	2.211	2.64
Female	Asthma	991,112	6.752	991,112	2.124	< 0.0001	3.038	2.991	3.086
Female	Food allergy	991,112	0.8165	991,112	0.3337	< 0.0001	2.223	2.135	2.315
Female	COPD	991,112	2.518	991,112	1.064	< 0.0001	2.166	2.117	2.216
Female	VMR/AR	991,112	11.539	991,112	2.864	< 0.0001	4.017	3.965	4.069
Female	EoE	991,112	0.137	991,112	0.0487	< 0.0001	2.487	2.241	2.76
Female	AD	991,112	1.117	991,112	0.3749	< 0.0001	2.71	2.611	2.813
Male	BP	715,006	0.0117	715,006	0.0053	0.0006	1.938	1.321	2.845
Male	PN	715,006	0.1631	715,006	0.071	< 0.0001	2.065	1.861	2.293
Male	Asthma	715,006	4.838	715,006	1.417	< 0.0001	3.278	3.206	3.351
Male	Food allergy	715,006	0.5692	715,006	0.2296	< 0.0001	2.284	2.157	2.419
Male	COPD	715,006	2.777	715,006	1.304	< 0.0001	1.98	1.932	2.03
Male	VMR/AR	715,006	9.529	715,006	2.173	< 0.0001	4.385	4.309	4.462
Male	EoE	715,006	0.2001	715,006	0.0755	< 0.0001	2.382	2.157	2.631
Male	AD	715,006	0.9973	715,006	0.2808	< 0.0001	3.29	3.131	3.457
White	BP	1,274,332	0.0123	1,274,332	0.0054	< 0.0001	1.992	1.5	2.645
White	PN	1,274,332	0.1828	1,274,332	0.0698	< 0.0001	2.377	2.2	2.568
White	Asthma	1,274,332	5.856	1,274,332	1.834	< 0.0001	3.097	3.052	3.143
White	Food allergy	1,274,332	0.68	1,274,332	0.2757	< 0.0001	2.292	2.203	2.383

KM-Analysis: CS									
**Analysis/Strata**	**Outcome**	**# (cases)**	**% (cases)**	**# (controls)**	**% (controls)**	**p-value**	**HR**	**HR (95%-CI lower limit)**	**HR (95%-CI upper limit)**
White	COPD	1,274,332	2.924	1,274,332	1.271	< 0.0001	2.158	2.119	2.198
White	VMR/AR	1,274,332	10.709	1,274,332	2.589	< 0.0001	4.183	4.133	4.233
White	EoE	1,274,332	0.1941	1,274,332	0.0718	< 0.0001	2.462	2.282	2.657
White	AD	1,274,332	0.9646	1,274,332	0.3076	< 0.0001	2.917	2.814	3.024
Black	BP	97,151	0.0113	97,151	0.0103	0.0953	2.559	0.8144	8.037
Black	PN	97,151	0.245	97,151	0.0896	< 0.0001	2.604	2.037	3.329
Black	Asthma	97,151	8.592	97,151	2.856	< 0.0001	3.081	2.951	3.216
Black	Food allergy	97,151	1.149	97,151	0.4508	< 0.0001	2.467	2.209	2.756
Black	COPD	97,151	2.628	97,151	1.291	< 0.0001	1.979	1.849	2.117
Black	VMR/AR	97,151	14.426	97,151	3.758	< 0.0001	4.137	3.989	4.291
Black	EoE	97,151	0.0669	97,151	0.0175	< 0.0001	3.628	2.126	6.188
Black	AD	97,151	1.713	97,151	0.5517	< 0.0001	3.03	2.749	3.34
Hispanic	BP	118,761	0.0084	118,761	0.0084	0.1012	0.3463	0.0918	1.307
Hispanic	PN	118,761	0.1078	118,761	0.0682	0.008	1.454	1.101	1.922
Hispanic	Asthma	118,761	6.996	118,761	2.001	< 0.0001	3.457	3.303	3.619
Hispanic	Food allergy	118,761	0.7385	118,761	0.2796	< 0.0001	2.474	2.18	2.807
Hispanic	COPD	118,761	1.352	118,761	0.5456	< 0.0001	2.322	2.12	2.544
Hispanic	VMR/AR	118,761	11.745	118,761	3.382	< 0.0001	3.569	3.446	3.697
Hispanic	EoE	118,761	0.0951	118,761	0.0345	< 0.0001	2.548	1.782	3.643
Hispanic	AD	118,761	1.262	118,761	0.3907	< 0.0001	3.057	2.754	3.393

Supplementary Table 14

KM-Analysis: AD									
Analysis/Strata	Outcome	# (cases)	% (cases)	# (controls)	% (controls)	p-value	HR	HR (95%-CI lower limit)	HR (95%-CI upper limit)
Primary	BP	733,240	0.013	733,240	0.0014	< 0.0001	15.494	6.79	35.357
Primary	PN	733,240	0.2299	733,240	0.0271	< 0.0001	8.244	7.117	9.549
Primary	Asthma	733,240	6.701	733,240	1.988	< 0.0001	3.434	3.371	3.498
Primary	Food allergy	733,240	2.106	733,240	0.4179	< 0.0001	4.956	4.767	5.152
Primary	COPD	733,240	0.6342	733,240	0.3696	< 0.0001	1.667	1.59	1.748
Primary	VMR/AR	733,240	10.024	733,240	2.54	< 0.0001	4.159	4.092	4.226
Primary	EoE	733,240	0.1195	733,240	0.0396	< 0.0001	2.909	2.547	3.323
Primary	CS	733,240	2.359	733,240	0.7931	< 0.0001	2.939	2.853	3.027
Primary	Fracture of clavicle	755,444	0.445	756,128	0.425	0.0132	1.063	1.013	1.116
S1	BP	781,335	0.0113	781,342	0.0022	< 0.0001	5.165	3.073	8.681
S1	PN	781,261	0.2038	781,338	0.0219	< 0.0001	9.261	7.909	10.843
S1	Asthma	780,100	6.666	780,528	1.9	< 0.0001	3.666	3.6	3.734
S1	Food allergy	780,627	2.094	780,964	0.393	< 0.0001	5.358	5.155	5.569
S1	COPD	781,210	0.6676	781,172	0.3767	< 0.0001	1.768	1.689	1.849
S1	VMR/AR	779,768	9.961	780,519	2.318	< 0.0001	4.65	4.575	4.725
S1	EoE	781,314	0.1174	781,331	0.0387	< 0.0001	3.018	2.65	3.438
S1	CS	780,985	2.525	781,132	0.8571	< 0.0001	2.985	2.903	3.068
S2	BP	205,203	0.0273	205,203	0.0049	< 0.0001	10.366	4.464	24.07
S2	PN	205,203	0.3309	205,203	0.0409	< 0.0001	9.427	7.513	11.829
S2	Asthma	205,203	5.765	205,203	2.39	< 0.0001	2.907	2.812	3.006
S2	Food allergy	205,203	1.318	205,203	0.4118	< 0.0001	3.751	3.472	4.053
S2	COPD	205,203	0.9824	205,203	0.7256	< 0.0001	1.602	1.498	1.713
S2	VMR/AR	205,203	8.548	205,203	2.894	< 0.0001	3.687	3.58	3.798
S2	EoE	205,203	0.1316	205,203	0.0585	< 0.0001	2.745	2.212	3.406
S2	CS	205,203	3.023	205,203	1.286	< 0.0001	2.811	2.686	2.943
S3	BP	103,286	0.0097	103,286	0.0097	0.0103	9.213	1.167	72.744
S3	PN	103,286	0.2072	103,286	0.0358	< 0.0001	6.118	4.314	8.676
S3	Asthma	103,286	4.462	103,286	2.934	< 0.0001	1.604	1.532	1.679
S3	Food allergy	103,286	1.347	103,286	0.6526	< 0.0001	2.158	1.968	2.366
S3	COPD	103,286	0.4173	103,286	0.64	< 0.0001	0.6805	0.6026	0.7684
S3	VMR/AR	103,286	6.464	103,286	2.957	< 0.0001	2.375	2.275	2.479
S3	EoE	103,286	0.0852	103,286	0.0639	< 0.0001	1.39	1.01	1.914
S3	CS	103,286	1.533	103,286	1.124	< 0.0001	1.433	1.328	1.546
S4	BP	391,609	0.038	391,609	0.006	< 0.0001	6.51	4.229	10.022
S4	PN	391,609	0.42	391,609	0.052	< 0.0001	8.727	7.54	10.1
S4	Asthma	391,609	4.899	391,609	1.883	< 0.0001	2.886	2.81	2.965
S4	Food allergy	391,609	1.197	391,609	0.365	< 0.0001	3.552	3.348	3.769
S4	COPD	391,609	1.682	391,609	0.777	< 0.0001	2.364	2.265	2.468
S4	VMR/AR	391,609	7.759	391,609	2.122	< 0.0001	4.154	4.055	4.257
S4	EoE	391,609	0.146	391,609	0.071	< 0.0001	2.241	1.941	2.587
S4	CS	391,609	3.116	391,609	1.232	< 0.0001	2.796	2.704	2.891
Adult	BP	234,464	0.0324	234,464	0.0047	2.44e-12	6.968	3.703	13.115

KM-Analysis: AD									
Analysis/Strata	Outcome	# (cases)	% (cases)	# (controls)	% (controls)	p-value	HR	HR (95%-CI lower limit)	HR (95%-CI upper limit)
Adult	PN	234,464	0.6632	234,464	0.0729	2.06e-247	9.225	7.877	10.803
Adult	Asthma	234,464	4.406	234,464	1.834	0	2.5	2.413	2.591
Adult	Food allergy	234,464	0.6419	234,464	0.3224	2.80e-58	2.021	1.851	2.205
Adult	COPD	234,464	1.811	234,464	1.176	3.10e-78	1.575	1.501	1.653
Adult	VMR/AR	234,464	6.792	234,464	2.418	0	2.987	2.898	3.079
Adult	EoE	234,464	0.1518	234,464	0.0478	2.35e-30	3.232	2.613	3.997
Adult	CS	234,464	3.377	234,464	1.43	0	2.455	2.358	2.556
Adolescent	BP	NA	NA	NA	NA	NA	NA	NA	NA
Adolescent	PN	63,591	0.165	63,591	0.017	< 0.0001	10.629	5.711	19.782
Adolescent	Asthma	63,591	4.968	63,591	3.035	< 0.0001	1.856	1.754	1.964
Adolescent	Food allergy	63,591	1.53	63,591	0.695	< 0.0001	2.459	2.197	2.751
Adolescent	COPD	63,591	0.053	63,591	0.039	0.1056	1.527	0.911	2.56
Adolescent	VMR/AR	63,591	7.223	63,591	4.664	< 0.0001	1.765	1.685	1.848
Adolescent	EoE	63,591	0.113	63,591	0.071	0.0025	1.765	1.216	2.561
Adolescent	CS	63,591	1.797	63,591	1.736	0.0007	1.153	1.061	1.252
Childhood	BP	349,034	0.0029	349,034	0.0029	0.5334	2.107	0.191	23.248
Childhood	PN	349,034	0.0653	349,034	0.0109	< 0.0001	6.334	4.492	8.93
Childhood	Asthma	349,034	7.556	349,034	2.665	< 0.0001	3.121	3.048	3.196
Childhood	Food allergy	349,034	2.189	349,034	0.5633	< 0.0001	4.127	3.927	4.337
Childhood	COPD	349,034	0.039	349,034	0.016	< 0.0001	2.53	1.853	3.454
Childhood	VMR/AR	349,034	11.584	349,034	4.032	< 0.0001	3.292	3.229	3.356
Childhood	EoE	349,034	0.1198	349,034	0.049	< 0.0001	2.576	2.156	3.078
Childhood	CS	349,034	1.961	349,034	0.975	< 0.0001	2.136	2.05	2.225
Female	BP	370,568	0.0143	370,568	0.0027	< 0.0001	6.726	3.198	14.148
Female	PN	370,568	0.2259	370,568	0.024	< 0.0001	9.476	7.615	11.79
Female	Asthma	370,568	6.169	370,568	2.012	< 0.0001	3.23	3.146	3.315
Female	Food allergy	370,568	1.961	370,568	0.4374	< 0.0001	4.559	4.32	4.811
Female	COPD	370,568	0.632	370,568	0.3451	< 0.0001	1.86	1.738	1.992
Female	VMR/AR	370,568	10.073	370,568	2.634	< 0.0001	4.18	4.088	4.275
Female	EoE	370,568	0.0847	370,568	0.0305	< 0.0001	2.784	2.245	3.453
Female	CS	370,568	2.818	370,568	0.9453	< 0.0001	3.075	2.96	3.195
Male	BP	284,571	0.0102	284,571	0.0035	6.07e-05	5.592	2.165	14.446
Male	PN	284,571	0.1655	284,571	0.019	< 0.0001	8.389	6.33	11.118
Male	Asthma	284,571	8.089	284,571	2.106	< 0.0001	3.912	3.803	4.025
Male	Food allergy	284,571	2.589	284,571	0.4997	< 0.0001	5.058	4.779	5.354
Male	COPD	284,571	0.6118	284,571	0.3433	< 0.0001	1.713	1.584	1.852
Male	VMR/AR	284,571	11.763	284,571	2.565	< 0.0001	4.854	4.732	4.978
Male	EoE	284,571	0.1553	284,571	0.0517	< 0.0001	2.878	2.388	3.469
Male	CS	284,571	2.572	284,571	0.758	< 0.0001	3.326	3.17	3.489
White	BP	337,469	0.0154	337,469	0.003	< 0.0001	13.039	4.716	36.05
White	PN	337,469	0.2676	337,469	0.0323	< 0.0001	8.357	6.85	10.194
White	Asthma	337,469	6.369	337,469	2.081	< 0.0001	3.215	3.13	3.303
White	Food allergy	337,469	2.002	337,469	0.448	< 0.0001	4.543	4.297	4.804

KM-Analysis: AD									
Analysis/Strata	Outcome	# (cases)	% (cases)	# (controls)	% (controls)	p-value	HR	HR (95%-CI lower limit)	HR (95%-CI upper limit)
White	COPD	337,469	1.015	337,469	0.5772	< 0.0001	1.779	1.683	1.881
White	VMR/AR	337,469	10.402	337,469	2.888	< 0.0001	3.926	3.839	4.015
White	EoE	337,469	0.1805	337,469	0.0652	< 0.0001	2.795	2.396	3.261
White	CS	337,469	3.496	337,469	1.183	< 0.0001	3.058	2.95	3.169
Black	BP	176,932	0.0102	176,932	0	< 0.0001	NA	NA	NA
Black	PN	176,932	0.2114	176,932	0.0232	< 0.0001	8.464	6.131	11.686
Black	Asthma	176,932	9.985	176,932	2.931	< 0.0001	3.381	3.278	3.487
Black	Food allergy	176,932	2.622	176,932	0.5793	< 0.0001	4.247	3.969	4.544
Black	COPD	176,932	0.507	176,932	0.2956	< 0.0001	1.579	1.418	1.759
Black	VMR/AR	176,932	13.704	176,932	3.193	< 0.0001	4.404	4.278	4.533
Black	EoE	176,932	0.0808	176,932	0.0277	< 0.0001	2.637	1.906	3.649
Black	CS	176,932	1.977	176,932	0.6404	< 0.0001	2.899	2.711	3.1
Hispanic	BP	70,339	0.0142	70,339	0	0.159	NA	NA	NA
Hispanic	PN	70,339	0.1336	70,339	0.0213	< 0.0001	6.214	3.603	10.716
Hispanic	Asthma	70,339	7.334	70,339	2.053	< 0.0001	3.728	3.517	3.953
Hispanic	Food allergy	70,339	1.774	70,339	0.3085	< 0.0001	5.734	4.964	6.624
Hispanic	COPD	70,339	0.1933	70,339	0.0967	< 0.0001	1.976	1.477	2.644
Hispanic	VMR/AR	70,339	13.29	70,339	3.5	< 0.0001	4.161	3.98	4.35
Hispanic	EoE	70,339	0.0611	70,339	0.0171	< 0.0001	3.568	1.882	6.767
Hispanic	CS	70,339	1.786	70,339	0.5033	< 0.0001	3.549	3.155	3.994

Supplementary Table 15

KM-Analysis: Food allergy									
**Analysis/Strata**	**Outcome**	**# (cases)**	**% (cases)**	**# (controls)**	**% (controls)**	**p-value**	**HR**	**HR (95%-CI lower limit)**	**HR (95%-CI upper limit)**
Primary	BP	426,574	0.003	426,574	0.0045	0.8553	1.069	0.5235	2.181
Primary	PN	426,574	0.0558	426,574	0.0368	< 0.0001	2.155	1.759	2.639
Primary	Asthma	426,574	4.78	426,574	1.897	< 0.0001	3.5	3.41	3.592
Primary	AD	426,574	2.066	426,574	0.4564	< 0.0001	5.939	5.654	6.239
Primary	COPD	426,574	0.7902	426,574	0.643	< 0.0001	1.661	1.579	1.747
Primary	VMR/AR	426,574	5.734	426,574	2.551	< 0.0001	3.18	3.109	3.253
Primary	EoE	426,574	0.2668	426,574	0.0452	< 0.0001	8.008	6.871	9.334
Primary	CS	426,574	1.825	426,574	1.01	< 0.0001	2.502	2.41	2.597
Primary	Fracture of clavicle	393,802	0.322	394,285	0.381	0.3341	1.038	0.963	1.118
S1	BP	366,642	0.003	366,642	0.0027	0.0217	3.26	1.123	9.462
S1	PN	366,629	0.0627	366,632	0.0357	< 0.0001	2.493	2.008	3.094
S1	Asthma	364,603	4.542	365,720	1.726	< 0.0001	3.661	3.556	3.769
S1	AD	365,132	1.688	366,403	0.4146	< 0.0001	5.382	5.087	5.694
S1	COPD	366,211	0.7518	366,332	0.6145	< 0.0001	1.655	1.565	1.751
S1	VMR/AR	363,658	5.237	365,650	2.21	< 0.0001	3.336	3.25	3.425
S1	EoE	366,438	0.2393	366,624	0.0436	< 0.0001	7.572	6.393	8.968
S1	CS	366,052	1.995	366,306	1.034	< 0.0001	2.651	2.549	2.757
S2	BP	145,830	0.0069	145,830	0.0089	0.7357	1.155	0.4999	2.668
S2	PN	145,830	0.1035	145,830	0.0617	< 0.0001	2.351	1.805	3.062
S2	Asthma	145,830	5.146	145,830	2.211	< 0.0001	3.183	3.053	3.319
S2	AD	145,830	1.657	145,830	0.4642	< 0.0001	4.712	4.324	5.135
S2	COPD	145,830	1.162	145,830	0.96	< 0.0001	1.617	1.506	1.737
S2	VMR/AR	145,830	6.624	145,830	2.734	< 0.0001	3.359	3.236	3.487
S2	EoE	145,830	0.3367	145,830	0.0651	< 0.0001	6.968	5.585	8.695
S2	CS	145,830	2.502	145,830	1.329	< 0.0001	2.602	2.461	2.751
S3	BP	92,400	0.0108	92,400	0.0108	0.1634	0.3403	0.0696	1.663
S3	PN	92,400	0.0379	92,400	0.0498	0.8583	0.9601	0.614	1.501
S3	Asthma	92,400	3.731	92,400	2.451	< 0.0001	1.805	1.71	1.904
S3	AD	92,400	1.329	92,400	0.592	< 0.0001	2.633	2.378	2.915
S3	COPD	92,400	0.7219	92,400	0.8777	0.4014	0.9567	0.8627	1.061
S3	VMR/AR	92,400	4.461	92,400	3.084	< 0.0001	1.739	1.657	1.825
S3	EoE	92,400	0.1894	92,400	0.0671	< 0.0001	3.591	2.677	4.818
S3	CS	92,400	1.496	92,400	1.39	< 0.0001	1.279	1.185	1.381
S4	BP	349,282	0.008	349,282	0.006	0.0492	1.779	0.994	3.184
S4	PN	349,282	0.099	349,282	0.047	< 0.0001	2.94	2.437	3.546
S4	Asthma	349,282	4.903	349,282	1.956	< 0.0001	3.329	3.236	3.425
S4	AD	349,282	1.472	349,282	0.401	< 0.0001	4.758	4.483	5.05
S4	COPD	349,282	1.342	349,282	0.76	< 0.0001	2.322	2.213	2.436
S4	VMR/AR	349,282	6.053	349,282	2.16	< 0.0001	3.756	3.658	3.857
S4	EoE	349,282	0.309	349,282	0.07	< 0.0001	5.825	5.063	6.702
S4	CS	349,282	2.471	349,282	1.253	< 0.0001	2.654	2.558	2.753
Adult	BP	248,697	0.0052	248,697	0.0048	0.2137	1.646	0.7445	3.64

KM-Analysis: Food allergy									
Analysis/Strata	Outcome	# (cases)	% (cases)	# (controls)	% (controls)	p-value	HR	HR (95%-CI lower limit)	HR (95%-CI upper limit)
Adult	PN	248,697	0.0989	248,697	0.0655	< 0.0001	2.351	1.923	2.875
Adult	Asthma	248,697	3.822	248,697	1.87	< 0.0001	2.952	2.85	3.059
Adult	AD	248,697	0.6506	248,697	0.3084	< 0.0001	3.096	2.838	3.377
Adult	COPD	248,697	1.414	248,697	1.194	< 0.0001	1.71	1.628	1.797
Adult	VMR/AR	248,697	4.568	248,697	2.465	< 0.0001	2.687	2.604	2.772
Adult	EoE	248,697	0.2083	248,697	0.0567	< 0.0001	5.392	4.467	6.509
Adult	CS	248,697	2.192	248,697	1.41	< 0.0001	2.283	2.187	2.383
Adolescent	BP	NA	NA	NA	NA	NA	NA	NA	NA
Adolescent	PN	NA	NA	NA	NA	NA	NA	NA	NA
Adolescent	Asthma	44,075	5.754	44,075	3.106	< 0.0001	2.585	2.419	2.763
Adolescent	AD	44,075	1.649	44,075	0.908	< 0.0001	2.524	2.232	2.854
Adolescent	COPD	44,075	0.064	44,075	0.043	0.0075	2.193	1.216	3.956
Adolescent	VMR/AR	44,075	6.575	44,075	4.828	< 0.0001	1.848	1.746	1.955
Adolescent	EoE	44,075	0.663	44,075	0.093	< 0.0001	9.295	6.692	12.91
Adolescent	CS	44,075	1.686	44,075	1.956	0.002	1.168	1.058	1.289
Childhood	BP	123,429	0	123,429	0	1	NA	NA	NA
Childhood	PN	123,429	0.0089	123,429	0.0105	0.8493	1.081	0.4836	2.417
Childhood	Asthma	123,429	7.344	123,429	2.787	< 0.0001	3.526	3.39	3.668
Childhood	AD	123,429	3.57	123,429	0.8718	< 0.0001	5.215	4.878	5.575
Childhood	COPD	123,429	0.0429	123,429	0.0154	< 0.0001	3.551	2.096	6.014
Childhood	VMR/AR	123,429	9.273	123,429	4.451	< 0.0001	2.854	2.763	2.947
Childhood	EoE	123,429	0.4383	123,429	0.0591	< 0.0001	9.815	7.68	12.544
Childhood	CS	123,429	1.636	123,429	1.14	< 0.0001	1.898	1.773	2.033
Female	BP	159,632	0.0063	159,632	0.0063	0.4129	1.637	0.4972	5.391
Female	PN	159,632	0.0658	159,632	0.0395	< 0.0001	2.35	1.716	3.218
Female	Asthma	159,632	4.83	159,632	2.087	< 0.0001	3.146	3.02	3.277
Female	AD	159,632	2.11	159,632	0.4955	< 0.0001	5.466	5.058	5.908
Female	COPD	159,632	0.7918	159,632	0.706	< 0.0001	1.486	1.371	1.611
Female	VMR/AR	159,632	6.215	159,632	2.763	< 0.0001	3.08	2.972	3.192
Female	EoE	159,632	0.1917	159,632	0.0363	< 0.0001	7.228	5.448	9.588
Female	CS	159,632	2.263	159,632	1.268	< 0.0001	2.423	2.294	2.559
Male	BP	174,506	0.0057	174,506	0.008	0.0964	0.4015	0.1319	1.222
Male	PN	174,506	0.0447	174,506	0.0292	< 0.0001	2.162	1.516	3.085
Male	Asthma	174,506	4.647	174,506	1.71	< 0.0001	3.699	3.546	3.858
Male	AD	174,506	2.125	174,506	0.463	< 0.0001	5.923	5.489	6.393
Male	COPD	174,506	0.8487	174,506	0.6871	< 0.0001	1.635	1.515	1.765
Male	VMR/AR	174,506	5.278	174,506	2.412	< 0.0001	3.025	2.916	3.137
Male	EoE	174,506	0.3129	174,506	0.0602	< 0.0001	6.904	5.599	8.512
Male	CS	174,506	1.556	174,506	0.9226	< 0.0001	2.296	2.159	2.443
White	BP	224,072	0.0045	224,072	0.0045	0.607	1.278	0.5006	3.263
White	PN	224,072	0.0611	224,072	0.0558	< 0.0001	1.747	1.368	2.233
White	Asthma	224,072	4.555	224,072	2.087	< 0.0001	3.278	3.165	3.395
White	AD	224,072	1.429	224,072	0.3664	< 0.0001	5.468	5.062	5.907

KM-Analysis: Food allergy									
**Analysis/Strata**	**Outcome**	**# (cases)**	**% (cases)**	**# (controls)**	**% (controls)**	**p-value**	**HR**	**HR (95%-CI lower limit)**	**HR (95%-CI upper limit)**
White	COPD	224,072	1.016	224,072	0.9091	< 0.0001	1.639	1.543	1.741
White	VMR/AR	224,072	5.342	224,072	2.691	< 0.0001	3.009	2.916	3.104
White	EoE	224,072	0.316	224,072	0.079	< 0.0001	6.102	5.166	7.208
White	CS	224,072	2.222	224,072	1.381	< 0.0001	2.45	2.341	2.563
Black	BP	73,407	0.0136	73,407	0.0136	0.8404	0.837	0.1477	4.742
Black	PN	73,407	0.0504	73,407	0.045	0.0115	1.834	1.138	2.955
Black	Asthma	73,407	5.787	73,407	2.535	< 0.0001	3.2	3.029	3.38
Black	AD	73,407	2.426	73,407	0.6362	< 0.0001	5.136	4.636	5.69
Black	COPD	73,407	0.861	73,407	0.8187	< 0.0001	1.469	1.312	1.644
Black	VMR/AR	73,407	6.13	73,407	2.979	< 0.0001	2.937	2.789	3.092
Black	EoE	73,407	0.1063	73,407	0.0163	< 0.0001	8.67	4.712	15.955
Black	CS	73,407	1.334	73,407	0.8473	< 0.0001	2.207	1.994	2.442
Hispanic	BP	36,734	0	36,734	0.0272	0.3736	NA	NA	NA
Hispanic	PN	36,734	0.0354	36,734	0.0354	0.5831	1.24	0.5741	2.679
Hispanic	Asthma	36,734	6.278	36,734	2.129	< 0.0001	3.709	3.419	4.023
Hispanic	AD	36,734	3.485	36,734	0.6751	< 0.0001	6.102	5.326	6.992
Hispanic	COPD	36,734	0.3376	36,734	0.2858	< 0.0001	1.443	1.112	1.874
Hispanic	VMR/AR	36,734	8.338	36,734	3.09	< 0.0001	3.464	3.236	3.709
Hispanic	EoE	36,734	0.1661	36,734	0.0272	< 0.0001	7.174	3.674	14.007
Hispanic	CS	36,734	1.524	36,734	0.6479	< 0.0001	2.876	2.471	3.348

Supplementary Table 16

KM-Analysis: VMR/AR									
Analysis/Strata	Outcome	# (cases)	% (cases)	# (controls)	% (controls)	p-value	HR	HR (95%-CI lower limit)	HR (95%-CI upper limit)
Primary	BP	3,336,609	0.0079	3,336,609	0.0049	0.0011	1.383	1.138	1.681
Primary	PN	3,336,609	0.129	3,336,609	0.0535	< 0.0001	2.097	1.985	2.217
Primary	Asthma	3,336,609	4.665	3,336,609	1.769	< 0.0001	2.399	2.376	2.422
Primary	Food allergy	3,336,609	0.5848	3,336,609	0.2832	< 0.0001	1.831	1.786	1.876
Primary	COPD	3,336,609	1.375	3,336,609	0.8988	< 0.0001	1.355	1.336	1.375
Primary	AD	3,336,609	1.067	3,336,609	0.3275	< 0.0001	2.903	2.842	2.966
Primary	EoE	3,336,609	0.1209	3,336,609	0.0493	< 0.0001	2.148	2.028	2.274
Primary	CS	3,336,609	3.781	3,336,609	1.188	< 0.0001	2.888	2.855	2.92
Primary	Fracture of clavicle	3,508,584	0.457	3,510,707	0.398	0.3816	0.99	0.968	1.013
S1	BP	3,210,677	0.0093	3,210,682	0.005	< 0.0001	1.553	1.282	1.881
S1	PN	3,210,476	0.1334	3,210,544	0.0456	< 0.0001	2.496	2.352	2.648
S1	Asthma	3,192,857	4.609	3,202,053	1.581	< 0.0001	2.612	2.586	2.638
S1	Food allergy	3,208,418	0.7255	3,208,978	0.2979	< 0.0001	2.11	2.06	2.161
S1	COPD	3,206,381	1.47	3,206,997	0.8282	< 0.0001	1.546	1.523	1.57
S1	AD	3,207,494	1.141	3,209,406	0.3221	< 0.0001	3.105	3.038	3.174
S1	EoE	3,210,297	0.1238	3,210,469	0.0486	< 0.0001	2.183	2.059	2.315
S1	CS	3,195,427	3.915	3,207,096	1.184	< 0.0001	2.981	2.947	3.015
S2	BP	1,421,326	0.0108	1,421,326	0.007	0.0007	1.542	1.199	1.983
S2	PN	1,421,326	0.1455	1,421,326	0.0654	< 0.0001	2.218	2.052	2.396
S2	Asthma	1,421,326	4.754	1,421,326	2.004	< 0.0001	2.393	2.36	2.426
S2	Food allergy	1,421,326	0.5705	1,421,326	0.3037	< 0.0001	1.863	1.795	1.933
S2	COPD	1,421,326	1.763	1,421,326	1.185	< 0.0001	1.479	1.451	1.509
S2	AD	1,421,326	1.019	1,421,326	0.3524	< 0.0001	2.879	2.788	2.973
S2	EoE	1,421,326	0.1323	1,421,326	0.056	< 0.0001	2.35	2.163	2.553
S2	CS	1,421,326	4.572	1,421,326	1.53	< 0.0001	3.025	2.979	3.072
S3	BP	823,631	0.0073	823,631	0.0058	0.9974	0.9994	0.6832	1.462
S3	PN	823,631	0.1166	823,631	0.0796	0.001	1.181	1.069	1.304
S3	Asthma	823,631	2.934	823,631	2.315	< 0.0001	1.061	1.041	1.082
S3	Food allergy	823,631	0.4232	823,631	0.3746	0.0139	0.9409	0.8963	0.9877
S3	COPD	823,631	0.9587	823,631	1.237	< 0.0001	0.6451	0.6264	0.6643
S3	AD	823,631	0.9038	823,631	0.4328	< 0.0001	1.724	1.656	1.794
S3	EoE	823,631	0.0839	823,631	0.0684	0.8771	1.009	0.9024	1.128
S3	CS	823,631	2.835	823,631	1.341	< 0.0001	1.757	1.718	1.797
S4	BP	2,198,645	0.017	2,198,645	0.007	< 0.0001	2.036	1.688	2.456
S4	PN	2,198,645	0.185	2,198,645	0.062	< 0.0001	2.61	2.455	2.776
S4	Asthma	2,198,645	4.985	2,198,645	1.547	< 0.0001	2.926	2.891	2.962
S4	Food allergy	2,198,645	0.752	2,198,645	0.254	< 0.0001	2.613	2.535	2.694
S4	COPD	2,198,645	2.717	2,198,645	1.028	< 0.0001	2.361	2.325	2.398
S4	AD	2,198,645	1.029	2,198,645	0.277	< 0.0001	3.282	3.191	3.376
S4	EoE	2,198,645	0.138	2,198,645	0.052	< 0.0001	2.337	2.183	2.502
S4	CS	2,198,645	5.229	2,198,645	1.33	< 0.0001	3.587	3.541	3.633
Adult	BP	2,242,633	0.0101	2,242,633	0.0052	< 0.0001	1.75	1.399	2.189

KM-Analysis: VMR/AR									
**Analysis/Strata**	**Outcome**	**# (cases)**	**% (cases)**	**# (controls)**	**% (controls)**	**p-value**	**HR**	**HR (95%-CI lower limit)**	**HR (95%-CI upper limit)**
Adult	PN	2,242,633	0.1814	2,242,633	0.0785	< 0.0001	2.083	1.97	2.203
Adult	Asthma	2,242,633	4.321	2,242,633	1.767	< 0.0001	2.274	2.247	2.3
Adult	Food allergy	2,242,633	0.4255	2,242,633	0.252	< 0.0001	1.536	1.486	1.588
Adult	COPD	2,242,633	1.81	2,242,633	1.23	< 0.0001	1.342	1.321	1.362
Adult	AD	2,242,633	0.7689	2,242,633	0.2662	< 0.0001	2.625	2.549	2.704
Adult	EoE	2,242,633	0.1355	2,242,633	0.0564	< 0.0001	2.177	2.039	2.325
Adult	CS	2,242,633	4.751	2,242,633	1.519	< 0.0001	2.924	2.889	2.96
Adolescent	BP	NA	NA	NA	NA	NA	NA	NA	NA
Adolescent	PN	292,017	0.039	292,017	0.02	< 0.0001	1.857	1.354	2.548
Adolescent	Asthma	292,017	6.377	292,017	3.02	< 0.0001	2.089	2.036	2.142
Adolescent	Food allergy	292,017	1.194	292,017	0.632	< 0.0001	1.809	1.71	1.914
Adolescent	COPD	292,017	0.078	292,017	0.045	< 0.0001	1.65	1.331	2.045
Adolescent	AD	292,017	1.599	292,017	0.77	< 0.0001	2.002	1.903	2.105
Adolescent	EoE	292,017	0.208	292,017	0.091	< 0.0001	2.182	1.889	2.521
Adolescent	CS	292,017	3.829	292,017	1.934	< 0.0001	1.928	1.867	1.99
Childhood	BP	788,705	0.0013	788,705	0.0013	0.1414	0.3203	0.0647	1.587
Childhood	PN	788,705	0.0314	788,705	0.0143	< 0.0001	2.104	1.684	2.628
Childhood	Asthma	788,705	7.933	788,705	3.33	< 0.0001	2.376	2.342	2.411
Childhood	Food allergy	788,705	1.384	788,705	0.701	< 0.0001	1.904	1.844	1.967
Childhood	COPD	788,705	0.0481	788,705	0.024	< 0.0001	1.923	1.615	2.289
Childhood	AD	788,705	2.536	788,705	1.039	< 0.0001	2.37	2.31	2.432
Childhood	EoE	788,705	0.16	788,705	0.0732	< 0.0001	2.098	1.902	2.316
Childhood	CS	788,705	3.206	788,705	1.477	< 0.0001	2.12	2.074	2.167
Female	BP	2,017,113	0.0081	2,017,113	0.0053	0.0319	1.305	1.023	1.666
Female	PN	2,017,113	0.1351	2,017,113	0.056	< 0.0001	2.112	1.97	2.263
Female	Asthma	2,017,113	5.351	2,017,113	2.077	< 0.0001	2.37	2.343	2.397
Female	Food allergy	2,017,113	0.8707	2,017,113	0.3929	< 0.0001	1.973	1.922	2.026
Female	COPD	2,017,113	1.537	2,017,113	0.9428	< 0.0001	1.457	1.431	1.483
Female	AD	2,017,113	1.284	2,017,113	0.3997	< 0.0001	2.894	2.823	2.968
Female	EoE	2,017,113	0.1009	2,017,113	0.0426	< 0.0001	2.088	1.927	2.261
Female	CS	2,017,113	4.396	2,017,113	1.425	< 0.0001	2.834	2.796	2.872
Male	BP	1,499,387	0.0083	1,499,387	0.0051	0.0207	1.399	1.051	1.862
Male	PN	1,499,387	0.127	1,499,387	0.0477	< 0.0001	2.313	2.123	2.521
Male	Asthma	1,499,387	4.419	1,499,387	1.579	< 0.0001	2.563	2.525	2.601
Male	Food allergy	1,499,387	0.6633	1,499,387	0.2969	< 0.0001	1.99	1.921	2.061
Male	COPD	1,499,387	1.535	1,499,387	0.9503	< 0.0001	1.437	1.408	1.468
Male	AD	1,499,387	1.161	1,499,387	0.3379	< 0.0001	3.085	2.99	3.184
Male	EoE	1,499,387	0.1673	1,499,387	0.0692	< 0.0001	2.121	1.973	2.281
Male	CS	1,499,387	3.583	1,499,387	1.095	< 0.0001	2.975	2.923	3.027
White	BP	2,152,224	0.0095	2,152,224	0.006	0.0016	1.424	1.142	1.776
White	PN	2,152,224	0.1411	2,152,224	0.0576	< 0.0001	2.221	2.079	2.373
White	Asthma	2,152,224	4.974	2,152,224	1.863	< 0.0001	2.536	2.507	2.565
White	Food allergy	2,152,224	0.747	2,152,224	0.3275	< 0.0001	2.103	2.045	2.163

KM-Analysis: VMR/AR									
**Analysis/Strata**	**Outcome**	**# (cases)**	**% (cases)**	**# (controls)**	**% (controls)**	**p-value**	**HR**	**HR (95%-CI lower limit)**	**HR (95%-CI upper limit)**
White	COPD	2,152,224	1.862	2,152,224	1.136	< 0.0001	1.52	1.496	1.544
White	AD	2,152,224	1.048	2,152,224	0.3248	< 0.0001	2.997	2.918	3.079
White	EoE	2,152,224	0.1659	2,152,224	0.0693	< 0.0001	2.189	2.06	2.325
White	CS	2,152,224	4.746	2,152,224	1.535	< 0.0001	2.94	2.904	2.977
Black	BP	550,179	0.0064	550,179	0.004	0.2838	1.338	0.7842	2.282
Black	PN	550,179	0.1309	550,179	0.0573	< 0.0001	2.014	1.764	2.3
Black	Asthma	550,179	5.944	550,179	2.671	< 0.0001	2.057	2.017	2.097
Black	Food allergy	550,179	1.158	550,179	0.5869	< 0.0001	1.762	1.689	1.839
Black	COPD	550,179	1.358	550,179	0.9806	< 0.0001	1.233	1.191	1.277
Black	AD	550,179	1.767	550,179	0.5985	< 0.0001	2.683	2.579	2.791
Black	EoE	550,179	0.0558	550,179	0.0251	< 0.0001	1.97	1.611	2.408
Black	CS	550,179	2.798	550,179	1.001	< 0.0001	2.548	2.471	2.628
Hispanic	BP	358,306	0.0028	358,306	0.0028	0.8723	0.9287	0.377	2.288
Hispanic	PN	358,306	0.0882	358,306	0.0413	< 0.0001	2.139	1.76	2.601
Hispanic	Asthma	358,306	4.711	358,306	2	< 0.0001	2.393	2.328	2.46
Hispanic	Food allergy	358,306	0.6087	358,306	0.355	< 0.0001	1.715	1.6	1.837
Hispanic	COPD	358,306	0.5696	358,306	0.4172	< 0.0001	1.369	1.28	1.463
Hispanic	AD	358,306	1.234	358,306	0.4097	< 0.0001	3.016	2.843	3.199
Hispanic	EoE	358,306	0.0815	358,306	0.0299	< 0.0001	2.773	2.222	3.461
Hispanic	CS	358,306	2.574	358,306	0.8889	< 0.0001	2.927	2.811	3.047

Supplementary Table 17

KM-Analysis: PN									
**Analysis/Strata**	**Outcome**	**# (cases)**	**% (cases)**	**# (controls)**	**% (controls)**	**p-value**	**HR**	**HR (95%-CI lower limit)**	**HR (95%-CI upper limit)**
Primary	BP	71,694	0.067	71,694	0.0139	< 0.0001	6.382	2.727	14.937
Primary	AD	71,694	2.76	71,694	0.2371	< 0.0001	9.623	8.226	11.256
Primary	Asthma	71,694	4.561	71,694	1.575	< 0.0001	2.365	2.21	2.532
Primary	Food allergy	71,694	0.5133	71,694	0.2497	< 0.0001	1.604	1.34	1.919
Primary	COPD	71,694	3.854	71,694	1.689	< 0.0001	1.829	1.709	1.957
Primary	VMR/AR	71,694	6.995	71,694	2.209	< 0.0001	2.631	2.486	2.784
Primary	EoE	71,694	0.1213	71,694	0.0446	0.0001	2.184	1.454	3.28
Primary	CS	71,694	3.786	71,694	1.28	< 0.0001	2.392	2.219	2.579
Primary	Fracture of clavicle	66,795	0.84	66,994	0.407	< 0.0001	1.766	1.528	2.042
S1	BP	68,895	0.0639	68,900	0.0145	< 0.0001	6.221	2.649	14.611
S1	AD	68,689	2.526	68,873	0.2715	< 0.0001	8.075	6.943	9.39
S1	Asthma	68,707	4.407	68,743	1.424	< 0.0001	2.694	2.506	2.895
S1	Food allergy	68,879	0.5357	68,874	0.2846	< 0.0001	1.581	1.329	1.88
S1	COPD	68,747	3.759	68,736	1.616	< 0.0001	1.989	1.854	2.135
S1	VMR/AR	68,637	6.223	68,722	2.075	< 0.0001	2.664	2.509	2.829
S1	EoE	68,895	0.1118	68,898	0.0363	< 0.0001	2.643	1.682	4.153
S1	CS	68,750	3.769	68,822	1.286	< 0.0001	2.557	2.37	2.758
S2	BP	27,185	0.0625	27,185	0.0368	0.0071	4.651	1.359	15.923
S2	AD	27,185	3.083	27,185	0.2796	< 0.0001	9.362	7.401	11.842
S2	Asthma	27,185	4.749	27,185	1.865	< 0.0001	2.151	1.94	2.384
S2	Food allergy	27,185	0.4046	27,185	0.2685	0.1292	1.258	0.9346	1.693
S2	COPD	27,185	4.263	27,185	1.935	< 0.0001	1.841	1.66	2.041
S2	VMR/AR	27,185	6.897	27,185	2.391	< 0.0001	2.495	2.282	2.728
S2	EoE	27,185	0.114	27,185	0.0478	0.0363	1.976	1.032	3.783
S2	CS	27,185	4.414	27,185	1.703	< 0.0001	2.203	1.978	2.453
S3	BP	11,255	0.0888	11,255	0.0888	0.1843	2.364	0.6384	8.755
S3	AD	11,255	2.701	11,255	0.3732	< 0.0001	5.543	4.012	7.659
S3	Asthma	11,255	3.27	11,255	2.106	0.0581	1.172	0.9944	1.381
S3	Food allergy	11,255	0.4442	11,255	0.2843	0.5161	1.159	0.742	1.811
S3	COPD	11,255	3.341	11,255	2.408	0.6843	1.033	0.8829	1.209
S3	VMR/AR	11,255	5.438	11,255	2.781	< 0.0001	1.514	1.321	1.736
S3	EoE	11,255	0.0888	11,255	0.0888	0.2654	1.917	0.598	6.145
S3	CS	11,255	3.003	11,255	1.555	< 0.0001	1.47	1.224	1.766
S4	BP	NA	NA	NA	NA	NA	NA	NA	NA
S4	AD	68,652	2.963	68,652	0.271	< 0.0001	10.042	8.642	11.669
S4	Asthma	68,652	4.698	68,652	1.478	< 0.0001	2.929	2.73	3.143
S4	Food allergy	68,652	0.644	68,652	0.267	< 0.0001	2.152	1.811	2.557
S4	COPD	68,652	3.827	68,652	1.369	< 0.0001	2.543	2.36	2.74
S4	VMR/AR	68,652	6.572	68,652	1.875	< 0.0001	3.278	3.081	3.487
S4	EoE	68,652	0.163	68,652	0.051	< 0.0001	2.832	1.937	4.14
S4	CS	68,652	3.671	68,652	1.229	< 0.0001	2.723	2.518	2.943
Adult	BP	53,323	0.0469	53,323	0.0188	0.0018	4.108	1.569	10.76

KM-Analysis: PN									
Analysis/Strata	Outcome	# (cases)	% (cases)	# (controls)	% (controls)	p-value	HR	HR (95%-CI lower limit)	HR (95%-CI upper limit)
Adult	AD	53,323	2.687	53,323	0.2494	< 0.0001	9.327	7.807	11.142
Adult	Asthma	53,323	4.889	53,323	1.581	< 0.0001	2.676	2.475	2.892
Adult	Food allergy	53,323	0.617	53,323	0.3094	< 0.0001	1.671	1.385	2.016
Adult	COPD	53,323	4.043	53,323	1.738	< 0.0001	1.956	1.811	2.114
Adult	VMR/AR	53,323	6.997	53,323	2.252	< 0.0001	2.752	2.578	2.937
Adult	EoE	53,323	0.1425	53,323	0.0694	0.0077	1.7	1.146	2.522
Adult	CS	53,323	4.22	53,323	1.38	< 0.0001	2.61	2.401	2.838
Adolescent	BP	1,086	0	1,086	0	1	NA	NA	NA
Adolescent	AD	NA	NA	NA	NA	NA	NA	NA	NA
Adolescent	Asthma	1,086	5.617	1,086	2.947	0.0046	1.841	1.199	2.827
Adolescent	Food allergy	NA	NA	NA	NA	NA	NA	NA	NA
Adolescent	COPD	NA	NA	NA	NA	NA	NA	NA	NA
Adolescent	VMR/AR	1,086	6.998	1,086	4.696	0.0334	1.468	1.028	2.095
Adolescent	EoE	NA	NA	NA	NA	NA	NA	NA	NA
Adolescent	CS	1,086	1.934	1,086	2.394	0.3521	0.761	0.427	1.356
Childhood	BP	1,243	0	1,243	0	1	NA	NA	NA
Childhood	AD	1,243	8.126	1,243	1.77	< 0.0001	4.755	2.998	7.543
Childhood	Asthma	1,243	6.677	1,243	5.551	0.3659	1.159	0.8416	1.596
Childhood	Food allergy	1,243	0.9654	1,243	0.8045	0.2265	1.817	0.6806	4.851
Childhood	COPD	1,243	0.8045	1,243	0	0.1726	NA	NA	NA
Childhood	VMR/AR	1,243	9.252	1,243	7.482	0.1819	1.205	0.9161	1.584
Childhood	EoE	1,243	0.8045	1,243	0.8045	0.0803	0.1817	0.0212	1.559
Childhood	CS	1,243	1.448	1,243	1.126	0.604	1.203	0.5975	2.423
Female	BP	22,375	0.0626	22,375	0.0447	0.0057	6.239	1.415	27.51
Female	AD	22,375	3.053	22,375	0.2682	< 0.0001	9.763	7.496	12.717
Female	Asthma	22,375	5.658	22,375	2.06	< 0.0001	2.362	2.122	2.629
Female	Food allergy	22,375	0.724	22,375	0.3397	< 0.0001	1.785	1.357	2.347
Female	COPD	22,375	3.651	22,375	1.774	< 0.0001	1.692	1.5	1.909
Female	VMR/AR	22,375	7.96	22,375	3.102	< 0.0001	2.252	2.062	2.459
Female	EoE	22,375	0.1073	22,375	0.0447	0.0685	1.968	0.9372	4.134
Female	CS	22,375	4.594	22,375	1.649	< 0.0001	2.376	2.108	2.677
Male	BP	25,410	0.0787	25,410	0.0394	0.0058	4.056	1.382	11.898
Male	AD	25,410	2.79	25,410	0.2519	< 0.0001	9.715	7.52	12.55
Male	Asthma	25,410	3.562	25,410	1.299	< 0.0001	2.359	2.079	2.677
Male	Food allergy	25,410	0.3621	25,410	0.181	< 0.0001	1.674	1.174	2.388
Male	COPD	25,410	4.459	25,410	2.18	< 0.0001	1.747	1.577	1.934
Male	VMR/AR	25,410	5.565	25,410	2.302	< 0.0001	2.127	1.931	2.342
Male	EoE	25,410	0.1653	25,410	0.059	0.0027	2.406	1.332	4.346
Male	CS	25,410	3.479	25,410	1.279	< 0.0001	2.343	2.063	2.662
White	BP	37,340	0.0884	37,340	0.0268	< 0.0001	6.932	2.451	19.604
White	AD	37,340	2.509	37,340	0.2678	< 0.0001	8.258	6.718	10.151
White	Asthma	37,340	4.858	37,340	1.679	< 0.0001	2.557	2.334	2.8
White	Food allergy	37,340	0.5892	37,340	0.3214	0.0001	1.542	1.234	1.928

KM-Analysis: PN									
**Analysis/Strata**	**Outcome**	**# (cases)**	**% (cases)**	**# (controls)**	**% (controls)**	**p-value**	**HR**	**HR (95%-CI lower limit)**	**HR (95%-CI upper limit)**
White	COPD	37,340	4.655	37,340	2.196	< 0.0001	1.831	1.684	1.99
White	VMR/AR	37,340	7.049	37,340	2.394	< 0.0001	2.658	2.464	2.868
White	EoE	37,340	0.1768	37,340	0.0616	0.0001	2.453	1.524	3.949
White	CS	37,340	4.646	37,340	1.695	< 0.0001	2.406	2.196	2.635
Black	BP	7,599	0.1316	7,599	0.1316	0.6786	1.654	0.1494	18.31
Black	AD	7,599	4.264	7,599	0.329	< 0.0001	10.263	6.827	15.427
Black	Asthma	7,599	7.001	7,599	2.474	< 0.0001	2.173	1.838	2.568
Black	Food allergy	7,599	1.04	7,599	0.5527	0.0793	1.4	0.9598	2.042
Black	COPD	7,599	4.685	7,599	2.04	< 0.0001	1.693	1.399	2.047
Black	VMR/AR	7,599	7.962	7,599	2.461	< 0.0001	2.571	2.181	3.032
Black	EoE	7,599	0.1316	7,599	0.1316	0.1303	4.484	0.5349	37.585
Black	CS	7,599	3.264	7,599	1.04	< 0.0001	2.334	1.809	3.012
Hispanic	BP	3,660	0.2732	3,660	0.2732	0.6669	1.685	0.1525	18.624
Hispanic	AD	3,660	2.404	3,660	0.4645	< 0.0001	4.165	2.475	7.01
Hispanic	Asthma	3,660	5.792	3,660	2.213	< 0.0001	2.082	1.61	2.692
Hispanic	Food allergy	3,660	0.7104	3,660	0.2732	0.0058	3.272	1.342	7.973
Hispanic	COPD	3,660	2.568	3,660	1.038	0.001	1.873	1.282	2.735
Hispanic	VMR/AR	3,660	9.454	3,660	3.415	< 0.0001	2.328	1.897	2.858
Hispanic	EoE	3,660	0.2732	3,660	0.2732	0.4541	2.325	0.2394	22.578
Hispanic	CS	3,660	3.115	3,660	1.339	0.0005	1.8	1.286	2.52

Supplementary Table 18

KM-Analysis: Asthma									
**Analysis/Strata**	**Outcome**	**# (cases)**	**% (cases)**	**# (controls)**	**% (controls)**	**p-value**	**HR**	**HR (95%-CI lower limit)**	**HR (95%-CI upper limit)**
Primary	BP	4,058,716	0.0041	4,058,716	0.0039	0.3493	1.11	0.8923	1.38
Primary	PN	4,058,716	0.05	4,058,716	0.0485	0.004	1.095	1.029	1.165
Primary	AD	4,058,716	2.937	4,058,716	2.544	< 0.0001	1.242	1.231	1.252
Primary	Food allergy	4,058,716	0.3876	4,058,716	0.3005	< 0.0001	1.374	1.342	1.407
Primary	COPD	4,058,716	1.861	4,058,716	0.705	< 0.0001	2.833	2.795	2.872
Primary	VMR/AR	4,058,716	0.4889	4,058,716	0.3497	< 0.0001	1.493	1.461	1.526
Primary	EoE	4,058,716	0.0739	4,058,716	0.0489	< 0.0001	1.606	1.518	1.7
Primary	CS	4,058,716	1.422	4,058,716	1.167	< 0.0001	1.304	1.288	1.32
Primary	Fracture of clavicle	4,205,640	0.394	4,208,829	0.369	< 0.0001	1.054	1.032	1.078
S1	BP	3,946,220	0.004	3,946,220	0.0037	0.316	1.122	0.8958	1.405
S1	PN	3,946,130	0.0493	3,946,073	0.0428	< 0.0001	1.192	1.117	1.273
S1	AD	3,933,375	2.706	3,932,976	2.255	< 0.0001	1.277	1.266	1.288
S1	Food allergy	3,944,493	0.4014	3,943,673	0.3359	< 0.0001	1.251	1.222	1.28
S1	COPD	3,933,403	1.609	3,942,611	0.6597	< 0.0001	2.58	2.543	2.618
S1	VMR/AR	3,944,371	0.4898	3,944,290	0.3512	< 0.0001	1.467	1.436	1.5
S1	EoE	3,945,791	0.0685	3,945,974	0.047	< 0.0001	1.517	1.43	1.609
S1	CS	3,940,494	1.384	3,941,609	1.157	< 0.0001	1.265	1.25	1.281
S2	BP	1,411,039	0.0063	1,411,039	0.0045	0.0022	1.647	1.192	2.276
S2	PN	1,411,039	0.059	1,411,039	0.0617	0.0553	1.097	0.9979	1.207
S2	AD	1,411,039	3.349	1,411,039	2.819	< 0.0001	1.348	1.331	1.367
S2	Food allergy	1,411,039	0.401	1,411,039	0.3434	< 0.0001	1.316	1.267	1.368
S2	COPD	1,411,039	2.378	1,411,039	0.9529	< 0.0001	2.83	2.774	2.887
S2	VMR/AR	1,411,039	0.5298	1,411,039	0.3964	< 0.0001	1.516	1.464	1.569
S2	EoE	1,411,039	0.0929	1,411,039	0.056	< 0.0001	1.884	1.724	2.058
S2	CS	1,411,039	1.927	1,411,039	1.442	< 0.0001	1.516	1.488	1.544
S3	BP	1,348,436	0.003	1,348,436	0.0048	0.0032	0.5596	0.3785	0.8275
S3	PN	1,348,436	0.0422	1,348,436	0.0589	< 0.0001	0.6314	0.5669	0.7032
S3	AD	1,348,436	2.105	1,348,436	3.117	< 0.0001	0.5988	0.5899	0.6079
S3	Food allergy	1,348,436	0.2742	1,348,436	0.3867	< 0.0001	0.6317	0.6056	0.6589
S3	COPD	1,348,436	1.355	1,348,436	0.8616	< 0.0001	1.393	1.361	1.426
S3	VMR/AR	1,348,436	0.3777	1,348,436	0.4435	< 0.0001	0.7581	0.7302	0.7869
S3	EoE	1,348,436	0.0585	1,348,436	0.067	< 0.0001	0.7712	0.7009	0.8486
S3	CS	1,348,436	1.11	1,348,436	1.566	< 0.0001	0.628	0.615	0.6413
S4	BP	2,124,548	0.01	2,124,548	0.007	< 0.0001	1.644	1.329	2.034
S4	PN	2,124,548	0.09	2,124,548	0.06	< 0.0001	1.627	1.516	1.746
S4	AD	2,124,548	3.243	2,124,548	1.955	< 0.0001	1.804	1.782	1.826
S4	Food allergy	2,124,548	0.419	2,124,548	0.258	< 0.0001	1.755	1.697	1.815
S4	COPD	2,124,548	3.776	2,124,548	0.985	< 0.0001	4.186	4.123	4.25
S4	VMR/AR	2,124,548	0.423	2,124,548	0.279	< 0.0001	1.645	1.592	1.7
S4	EoE	2,124,548	0.086	2,124,548	0.053	< 0.0001	1.751	1.625	1.886
S4	CS	2,124,548	2.097	2,124,548	1.339	< 0.0001	1.699	1.674	1.725
Adult	BP	2,893,083	0.005	2,893,083	0.0049	0.2672	1.14	0.9044	1.437

KM-Analysis: Asthma									
**Analysis/Strata**	**Outcome**	**# (cases)**	**% (cases)**	**# (controls)**	**% (controls)**	**p-value**	**HR**	**HR (95%-CI lower limit)**	**HR (95%-CI upper limit)**
Adult	PN	2,893,083	0.0704	2,893,083	0.066	< 0.0001	1.2	1.127	1.277
Adult	AD	2,893,083	2.548	2,893,083	2.441	< 0.0001	1.163	1.152	1.176
Adult	Food allergy	2,893,083	0.3453	2,893,083	0.3245	< 0.0001	1.184	1.151	1.218
Adult	COPD	2,893,083	2.463	2,893,083	0.9555	< 0.0001	2.891	2.851	2.931
Adult	VMR/AR	2,893,083	0.2914	2,893,083	0.2644	< 0.0001	1.231	1.193	1.269
Adult	EoE	2,893,083	0.0717	2,893,083	0.0515	< 0.0001	1.555	1.455	1.662
Adult	CS	2,893,083	1.673	2,893,083	1.357	< 0.0001	1.377	1.359	1.396
Adolescent	BP	NA	NA	NA	NA	NA	NA	NA	NA
Adolescent	PN	407,088	0.027	407,088	0.022	0.37	1.136	0.859	1.503
Adolescent	AD	407,088	6.574	407,088	4.545	< 0.0001	1.43	1.404	1.457
Adolescent	Food allergy	407,088	1.293	407,088	0.608	< 0.0001	2.027	1.933	2.127
Adolescent	COPD	407,088	0.231	407,088	0.036	< 0.0001	6.081	5.105	7.244
Adolescent	VMR/AR	407,088	1.087	407,088	0.787	< 0.0001	1.322	1.264	1.384
Adolescent	EoE	407,088	0.215	407,088	0.085	< 0.0001	2.426	2.142	2.748
Adolescent	CS	407,088	2.38	407,088	1.899	< 0.0001	1.203	1.168	1.24
Childhood	BP	854,305	0.0012	854,305	0.0012	0.1722	0.2446	0.0273	2.191
Childhood	PN	854,305	0.0191	854,305	0.016	0.1724	1.171	0.9331	1.471
Childhood	AD	854,305	7.373	854,305	4.98	< 0.0001	1.568	1.549	1.588
Childhood	Food allergy	854,305	1.265	854,305	0.6925	< 0.0001	1.838	1.781	1.898
Childhood	COPD	854,305	0.1455	854,305	0.022	< 0.0001	6.748	5.788	7.868
Childhood	VMR/AR	854,305	1.492	854,305	0.9906	< 0.0001	1.551	1.509	1.595
Childhood	EoE	854,305	0.1721	854,305	0.0707	< 0.0001	2.409	2.191	2.648
Childhood	CS	854,305	1.725	854,305	1.342	< 0.0001	1.313	1.281	1.345
Female	BP	1,848,079	0.005	1,848,079	0.0042	0.0539	1.345	0.994	1.819
Female	PN	1,848,079	0.058	1,848,079	0.0554	0.0006	1.161	1.066	1.265
Female	AD	1,848,079	0.5285	1,848,079	0.4162	< 0.0001	1.409	1.368	1.452
Female	Food allergy	1,848,079	0.5104	1,848,079	0.4278	< 0.0001	1.321	1.282	1.361
Female	COPD	1,848,079	2.078	1,848,079	0.7995	< 0.0001	2.897	2.842	2.952
Female	VMR/AR	1,848,079	3.252	1,848,079	2.992	< 0.0001	1.212	1.198	1.226
Female	EoE	1,848,079	0.0609	1,848,079	0.0426	< 0.0001	1.582	1.444	1.733
Female	CS	1,848,079	1.716	1,848,079	1.456	< 0.0001	1.312	1.291	1.333
Male	BP	1,426,597	0.0036	1,426,597	0.0029	0.1759	1.326	0.8801	1.997
Male	PN	1,426,597	0.0498	1,426,597	0.0411	< 0.0001	1.267	1.136	1.414
Male	AD	1,426,597	0.603	1,426,597	0.3931	< 0.0001	1.635	1.581	1.691
Male	Food allergy	1,426,597	0.4242	1,426,597	0.2985	< 0.0001	1.505	1.447	1.565
Male	COPD	1,426,597	1.798	1,426,597	0.6521	< 0.0001	2.946	2.876	3.016
Male	VMR/AR	1,426,597	3.283	1,426,597	2.454	< 0.0001	1.441	1.421	1.461
Male	EoE	1,426,597	0.1099	1,426,597	0.0745	< 0.0001	1.547	1.431	1.672
Male	CS	1,426,597	1.362	1,426,597	1.022	< 0.0001	1.419	1.389	1.45
White	BP	1,958,156	0.0051	1,958,156	0.0043	0.1141	1.264	0.9447	1.691
White	PN	1,958,156	0.0657	1,958,156	0.0483	< 0.0001	1.457	1.339	1.584
White	AD	1,958,156	0.4957	1,958,156	0.3558	< 0.0001	1.518	1.472	1.565
White	Food allergy	1,958,156	0.4886	1,958,156	0.3624	< 0.0001	1.463	1.418	1.508

KM-Analysis: Asthma									
**Analysis/Strata**	**Outcome**	**# (cases)**	**% (cases)**	**# (controls)**	**% (controls)**	**p-value**	**HR**	**HR (95%-CI lower limit)**	**HR (95%-CI upper limit)**
White	COPD	1,958,156	2.423	1,958,156	0.9033	< 0.0001	2.946	2.896	2.998
White	VMR/AR	1,958,156	3.468	1,958,156	2.634	< 0.0001	1.456	1.44	1.473
White	EoE	1,958,156	0.1145	1,958,156	0.0735	< 0.0001	1.674	1.567	1.789
White	CS	1,958,156	2.01	1,958,156	1.549	< 0.0001	1.421	1.4	1.442
Black	BP	864,700	0.0025	864,700	0.003	0.8706	0.9539	0.5406	1.683
Black	PN	864,700	0.0493	864,700	0.0593	0.3138	0.9361	0.8232	1.064
Black	AD	864,700	0.6822	864,700	0.5905	< 0.0001	1.306	1.258	1.356
Black	Food allergy	864,700	0.5515	864,700	0.5715	< 0.0001	1.088	1.046	1.132
Black	COPD	864,700	1.585	864,700	0.7215	< 0.0001	2.489	2.416	2.565
Black	VMR/AR	864,700	2.819	864,700	3.258	0.0158	0.9791	0.9625	0.996
Black	EoE	864,700	0.0305	864,700	0.0261	0.0021	1.32	1.105	1.577
Black	CS	864,700	0.804	864,700	0.9832	< 0.0001	0.9233	0.8945	0.9531
Hispanic	BP	434,424	0.0023	434,424	0.0037	0.2263	0.6072	0.2683	1.374
Hispanic	PN	434,424	0.0341	434,424	0.0394	0.4916	0.9257	0.7428	1.154
Hispanic	AD	434,424	0.7628	434,424	0.4747	< 0.0001	1.738	1.645	1.836
Hispanic	Food allergy	434,424	0.4311	434,424	0.3527	< 0.0001	1.315	1.229	1.407
Hispanic	COPD	434,424	0.9339	434,424	0.2988	< 0.0001	3.372	3.168	3.59
Hispanic	VMR/AR	434,424	3.912	434,424	3.276	< 0.0001	1.304	1.276	1.334
Hispanic	EoE	434,424	0.0467	434,424	0.0276	< 0.0001	1.804	1.44	2.261
Hispanic	CS	434,424	1.011	434,424	0.8008	< 0.0001	1.363	1.304	1.425

Supplementary Table 19

KM-Analysis: EoE									
Analysis/Strata	Outcome	# (cases)	% (cases)	# (controls)	% (controls)	p-value	HR	HR (95%-CI lower limit)	HR (95%-CI upper limit)
Primary	BP	66,822	0.015	66,822	0.015	0.1404	3.602	0.588	22.068
Primary	PN	66,822	0.0778	66,822	0.0374	< 0.0001	2.848	1.759	4.611
Primary	Asthma	66,822	3.56	66,822	1.709	< 0.0001	2.726	2.539	2.927
Primary	Food allergy	66,822	1.024	66,822	0.2574	< 0.0001	5.144	4.347	6.088
Primary	COPD	66,822	0.7183	66,822	0.6899	< 0.0001	1.356	1.192	1.542
Primary	VMR/AR	66,822	3.523	66,822	2.43	< 0.0001	1.878	1.762	2.002
Primary	AD	66,822	0.5013	66,822	0.2769	< 0.0001	2.393	1.996	2.868
Primary	CS	66,822	1.868	66,822	1.296	< 0.0001	1.905	1.746	2.079
Primary	Fracture of clavicle	78,821	0.308	78,885	0.447	0.2183	0.902	0.765	1.063
S1	BP	66,875	0.015	66,875	0.015	0.0868	3.851	0.7359	20.155
S1	PN	66,872	0.0852	66,873	0.0449	< 0.0001	2.711	1.736	4.232
S1	Asthma	66,594	3.25	66,717	1.413	< 0.0001	3.125	2.893	3.375
S1	Food allergy	66,767	0.9436	66,845	0.2783	< 0.0001	4.547	3.857	5.362
S1	COPD	66,810	0.6855	66,803	0.6856	< 0.0001	1.351	1.186	1.539
S1	VMR/AR	66,594	3.089	66,677	2.058	< 0.0001	2.007	1.874	2.15
S1	AD	66,841	0.4802	66,860	0.2453	< 0.0001	2.595	2.148	3.135
S1	CS	66,777	1.912	66,789	1.334	< 0.0001	1.943	1.784	2.116
S2	BP	29,396	0.034	29,396	0	0.2993	NA	NA	NA
S2	PN	29,396	0.1021	29,396	0.051	0.0008	2.792	1.494	5.216
S2	Asthma	29,396	3.752	29,396	2.109	< 0.0001	2.522	2.283	2.786
S2	Food allergy	29,396	0.9457	29,396	0.2109	< 0.0001	6.331	4.786	8.374
S2	COPD	29,396	0.8607	29,396	0.9423	0.0016	1.319	1.11	1.567
S2	VMR/AR	29,396	3.905	29,396	2.759	< 0.0001	2.034	1.857	2.229
S2	AD	29,396	0.5579	29,396	0.3164	< 0.0001	2.547	1.967	3.299
S2	CS	29,396	2.446	29,396	1.704	< 0.0001	2.054	1.83	2.306
S3	BP	20,070	0.0498	20,070	0.0498	0.8906	0.8442	0.0753	9.463
S3	PN	20,070	0.0498	20,070	0.0498	0.7835	1.135	0.4593	2.805
S3	Asthma	20,070	1.819	20,070	2.083	0.5245	1.047	0.9089	1.206
S3	Food allergy	20,070	0.5132	20,070	0.3139	< 0.0001	2.03	1.48	2.786
S3	COPD	20,070	0.2242	20,070	0.7125	< 0.0001	0.3744	0.2674	0.5241
S3	VMR/AR	20,070	1.669	20,070	2.651	< 0.0001	0.7354	0.6409	0.8437
S3	AD	20,070	0.2242	20,070	0.3288	0.3081	0.8202	0.5599	1.201
S3	CS	20,070	1.111	20,070	1.565	0.0589	0.8468	0.7124	1.006
S4	BP	NA	NA	NA	NA	NA	NA	NA	NA
S4	PN	79,042	0.086	79,042	0.052	< 0.0001	2.524	1.705	3.738
S4	Asthma	79,042	3.55	79,042	1.852	< 0.0001	2.732	2.563	2.913
S4	Food allergy	79,042	1.187	79,042	0.301	< 0.0001	5.439	4.712	6.276
S4	COPD	79,042	0.772	79,042	0.639	< 0.0001	1.717	1.524	1.935
S4	VMR/AR	79,042	3.546	79,042	2.161	< 0.0001	2.339	2.201	2.486
S4	AD	79,042	0.592	79,042	0.356	< 0.0001	2.421	2.084	2.812
S4	CS	79,042	2.05	79,042	1.335	< 0.0001	2.243	2.073	2.426
Adult	BP	50,051	0.02	50,051	0.02	0.6196	1.402	0.3669	5.359

KM-Analysis: EoE									
**Analysis/Strata**	**Outcome**	**# (cases)**	**% (cases)**	**# (controls)**	**% (controls)**	**p-value**	**HR**	**HR (95%-CI lower limit)**	**HR (95%-CI upper limit)**
Adult	PN	50,051	0.1179	50,051	0.0719	< 0.0001	2.352	1.545	3.579
Adult	Asthma	50,051	3.389	50,051	1.664	< 0.0001	2.658	2.444	2.89
Adult	Food allergy	50,051	0.6314	50,051	0.2198	< 0.0001	3.743	3.006	4.661
Adult	COPD	50,051	0.955	50,051	1.063	0.0066	1.189	1.049	1.347
Adult	VMR/AR	50,051	3.462	50,051	2.448	< 0.0001	1.841	1.71	1.982
Adult	AD	50,051	0.4256	50,051	0.2378	< 0.0001	2.39	1.904	3.001
Adult	CS	50,051	2.336	50,051	1.718	< 0.0001	1.78	1.629	1.945
Adolescent	BP	8,952	0	8,952	0	1	NA	NA	NA
Adolescent	PN	NA	NA	NA	NA	NA	NA	NA	NA
Adolescent	Asthma	8,952	4.245	8,952	3.117	< 0.0001	1.965	1.682	2.296
Adolescent	Food allergy	8,952	2.77	8,952	0.559	< 0.0001	7.229	5.329	9.806
Adolescent	COPD	NA	NA	NA	NA	NA	NA	NA	NA
Adolescent	VMR/AR	8,952	4.01	8,952	4.301	0.0001	1.325	1.147	1.532
Adolescent	AD	8,952	0.67	8,952	0.67	0.0495	1.431	0.999	2.049
Adolescent	CS	8,952	1.139	8,952	2.201	0.0202	0.753	0.592	0.957
Childhood	BP	10,095	0	10,095	0	1	NA	NA	NA
Childhood	PN	10,095	0.0991	10,095	0.0991	0.0827	5.616	0.6249	50.474
Childhood	Asthma	10,095	6.855	10,095	3.556	< 0.0001	2.758	2.427	3.136
Childhood	Food allergy	10,095	4.061	10,095	0.7628	< 0.0001	7.632	5.977	9.745
Childhood	COPD	10,095	0.109	10,095	0.0991	0.0531	2.592	0.9528	7.05
Childhood	VMR/AR	10,095	6.815	10,095	6.508	< 0.0001	1.464	1.315	1.63
Childhood	AD	10,095	1.486	10,095	0.9014	< 0.0001	2.265	1.745	2.941
Childhood	CS	10,095	1.506	10,095	1.902	0.3443	1.108	0.8954	1.372
Female	BP	25,351	0.0394	25,351	0.0394	0.8529	0.7939	0.0689	9.148
Female	PN	25,351	0.1144	25,351	0.0552	0.0003	3.056	1.606	5.813
Female	Asthma	25,351	4.576	25,351	2.292	< 0.0001	2.799	2.531	3.095
Female	Food allergy	25,351	1.475	25,351	0.4339	< 0.0001	4.646	3.749	5.756
Female	COPD	25,351	0.9664	25,351	0.8086	< 0.0001	1.687	1.399	2.035
Female	VMR/AR	25,351	4.394	25,351	2.887	< 0.0001	2.088	1.901	2.294
Female	AD	25,351	0.639	25,351	0.4497	< 0.0001	1.951	1.532	2.484
Female	CS	25,351	2.568	25,351	1.629	< 0.0001	2.238	1.976	2.536
Male	BP	41,883	0.0239	41,883	0.0239	0.0421	7.099	0.7875	63.993
Male	PN	41,883	0.0764	41,883	0.0645	0.0498	1.665	0.9951	2.787
Male	Asthma	41,883	2.918	41,883	1.514	< 0.0001	2.592	2.353	2.856
Male	Food allergy	41,883	1.022	41,883	0.2698	< 0.0001	4.918	3.993	6.057
Male	COPD	41,883	0.6638	41,883	0.7449	0.0241	1.206	1.025	1.419
Male	VMR/AR	41,883	3.178	41,883	2.335	< 0.0001	1.8	1.657	1.956
Male	AD	41,883	0.5109	41,883	0.2722	< 0.0001	2.573	2.046	3.237
Male	CS	41,883	1.717	41,883	1.263	< 0.0001	1.833	1.637	2.053
White	BP	58,450	0.0171	58,450	0.0171	0.1673	3.145	0.5679	17.415
White	PN	58,450	0.0941	58,450	0.0205	< 0.0001	6.774	3.612	12.702
White	Asthma	58,450	3.518	58,450	1.707	< 0.0001	2.817	2.61	3.04
White	Food allergy	58,450	1.076	58,450	0.3011	< 0.0001	4.816	4.07	5.699

KM-Analysis: EoE									
**Analysis/Strata**	**Outcome**	**# (cases)**	**% (cases)**	**# (controls)**	**% (controls)**	**p-value**	**HR**	**HR (95%-CI lower limit)**	**HR (95%-CI upper limit)**
White	COPD	58,450	0.7921	58,450	0.8195	< 0.0001	1.337	1.175	1.521
White	VMR/AR	58,450	3.521	58,450	2.469	< 0.0001	1.945	1.817	2.081
White	AD	58,450	0.4842	58,450	0.2943	< 0.0001	2.227	1.84	2.696
White	CS	58,450	2.18	58,450	1.547	< 0.0001	1.963	1.802	2.14
Black	BP	3,427	0.2918	3,427	0	0.1396	NA	NA	NA
Black	PN	3,427	0.2918	3,427	0.2918	0.6858	1.393	0.2776	6.991
Black	Asthma	3,427	6.332	3,427	2.393	< 0.0001	3.791	2.933	4.9
Black	Food allergy	3,427	2.364	3,427	0.4085	< 0.0001	8.156	4.612	14.422
Black	COPD	3,427	1.459	3,427	1.284	0.038	1.535	1.021	2.308
Black	VMR/AR	3,427	5.748	3,427	2.714	< 0.0001	2.91	2.27	3.73
Black	AD	3,427	1.284	3,427	0.4377	< 0.0001	4.043	2.242	7.29
Black	CS	3,427	2.276	3,427	0.8754	< 0.0001	3.588	2.346	5.489
Hispanic	BP	3,685	0	3,685	0	1	NA	NA	NA
Hispanic	PN	3,685	0.2714	3,685	0.2714	0.8512	1.158	0.2495	5.375
Hispanic	Asthma	3,685	4.505	3,685	2.008	< 0.0001	3.212	2.433	4.241
Hispanic	Food allergy	3,685	1.221	3,685	0.4071	< 0.0001	4.04	2.24	7.287
Hispanic	COPD	3,685	0.787	3,685	0.5427	0.0073	2.159	1.214	3.838
Hispanic	VMR/AR	3,685	4.722	3,685	3.175	< 0.0001	2.064	1.628	2.617
Hispanic	AD	3,685	0.9498	3,685	0.3528	< 0.0001	3.916	2.056	7.458
Hispanic	CS	3,685	1.682	3,685	1.085	< 0.0001	2.304	1.538	3.451

Supplementary Table 20

KM-Analysis: COPD									
Analysis/Strata	Outcome	# (cases)	% (cases)	# (controls)	% (controls)	p-value	HR	HR (95%-CI lower limit)	HR (95%-CI upper limit)
Primary	BP	1,906,021	0.0092	1,906,021	0.0167	0.0006	0.726	0.6035	0.8732
Primary	PN	1,906,021	0.059	1,906,021	0.0848	0.0201	0.9136	0.8466	0.9859
Primary	Asthma	1,906,021	2.362	1,906,021	1.364	< 0.0001	2.199	2.165	2.232
Primary	Food allergy	1,906,021	0.158	1,906,021	0.207	0.3876	0.9793	0.9339	1.027
Primary	AD	1,906,021	0.1323	1,906,021	0.2155	< 0.0001	0.7999	0.7612	0.8406
Primary	VMR/AR	1,906,021	1.251	1,906,021	2.14	< 0.0001	0.7503	0.7384	0.7624
Primary	EoE	1,906,021	0.0187	1,906,021	0.0322	< 0.0001	0.7546	0.6621	0.86
Primary	CS	1,906,021	0.8454	1,906,021	1.232	< 0.0001	0.8829	0.8654	0.9008
Primary	Fracture of clavicle	1,996,104	0.494	1,998,605	0.502	< 0.0001	1.211	1.177	1.245
S1	BP	1,806,561	0.009	1,806,547	0.0176	0.0009	0.728	0.6026	0.8794
S1	PN	1,806,494	0.0523	1,806,469	0.0762	0.3768	0.9633	0.8866	1.047
S1	Asthma	1,796,497	1.874	1,802,714	1.227	< 0.0001	2.106	2.071	2.142
S1	Food allergy	1,806,091	0.1595	1,805,815	0.2572	< 0.0001	0.8569	0.818	0.8978
S1	AD	1,806,371	0.1427	1,806,256	0.2451	< 0.0001	0.8163	0.7776	0.857
S1	VMR/AR	1,804,045	1.117	1,802,496	1.916	< 0.0001	0.8038	0.7899	0.8179
S1	EoE	1,806,542	0.0154	1,806,514	0.0294	< 0.0001	0.7321	0.6331	0.8465
S1	CS	1,804,743	0.7483	1,804,432	1.172	< 0.0001	0.8898	0.871	0.9089
S2	BP	697,656	0.0106	697,656	0.0155	0.4174	0.8847	0.6577	1.19
S2	PN	697,656	0.0906	697,656	0.1194	0.5684	0.9703	0.8748	1.076
S2	Asthma	697,656	2.772	697,656	1.516	< 0.0001	2.262	2.209	2.317
S2	Food allergy	697,656	0.1972	697,656	0.2606	0.1198	0.9458	0.8817	1.015
S2	AD	697,656	0.2	697,656	0.2792	0.0085	0.9117	0.851	0.9767
S2	VMR/AR	697,656	1.667	697,656	2.463	< 0.0001	0.8457	0.826	0.8658
S2	EoE	697,656	0.0245	697,656	0.0378	0.0381	0.8158	0.6727	0.9892
S2	CS	697,656	1.116	697,656	1.437	0.0897	0.9746	0.9462	1.004
S3	BP	635,437	0.0058	635,437	0.0219	< 0.0001	0.2754	0.1916	0.3958
S3	PN	635,437	0.0414	635,437	0.099	< 0.0001	0.4277	0.3704	0.494
S3	Asthma	635,437	1.14	635,437	1.658	< 0.0001	0.6937	0.6733	0.7148
S3	Food allergy	635,437	0.0705	635,437	0.2453	< 0.0001	0.291	0.2619	0.3232
S3	AD	635,437	0.0932	635,437	0.2652	< 0.0001	0.3593	0.3271	0.3945
S3	VMR/AR	635,437	0.7148	635,437	2.732	< 0.0001	0.2623	0.2539	0.271
S3	EoE	635,437	0.0091	635,437	0.0397	< 0.0001	0.2353	0.1769	0.3131
S3	CS	635,437	0.5318	635,437	1.645	< 0.0001	0.3259	0.3135	0.3388
S4	BP	1,632,835	0.012	1,632,835	0.014	0.7769	1.028	0.85	1.242
S4	PN	1,632,835	0.058	1,632,835	0.065	0.0189	1.111	1.017	1.212
S4	Asthma	1,632,835	2.722	1,632,835	1.091	< 0.0001	2.961	2.91	3.013
S4	Food allergy	1,632,835	0.195	1,632,835	0.175	< 0.0001	1.334	1.268	1.403
S4	AD	1,632,835	0.179	1,632,835	0.206	0.0093	1.068	1.016	1.123
S4	VMR/AR	1,632,835	1.531	1,632,835	1.522	< 0.0001	1.206	1.185	1.227
S4	EoE	1,632,835	0.022	1,632,835	0.032	0.0055	0.827	0.723	0.946
S4	CS	1,632,835	1.019	1,632,835	1.068	< 0.0001	1.143	1.119	1.168

Supplementary Table 21

KM-Analysis: BP									
Analysis/Strata	Outcome	# (cases)	% (cases)	# (controls)	% (controls)	p-value	HR	HR (95%-CI lower limit)	HR (95%-CI upper limit)
Primary	AD	6,458	0.8362	6,458	0.2168	< 0.0001	6.236	3.432	11.329
Primary	PN	6,458	0.2478	6,458	0.1548	0.0002	6.423	2.095	19.693
Primary	Asthma	6,458	1.022	6,458	1.177	0.0821	1.345	0.9619	1.88
Primary	Food allergy	6,458	0.2323	6,458	0.2787	0.3322	1.411	0.7011	2.841
Primary	COPD	6,458	1.967	6,458	2.354	0.0139	1.351	1.062	1.717
Primary	VMR/AR	6,458	1.517	6,458	2.183	0.4462	1.107	0.8521	1.438
Primary	EoE	6,458	0.1548	6,458	0.1548	0.5118	1.892	0.2756	12.983
Primary	CS	6,458	0.7587	6,458	0.8826	0.0674	1.435	0.9725	2.117
Primary	Fracture of clavicle	7,929	0.227	7,924	0.644	0.0304	0.553	0.321	0.953
S1	AD	6,839	0.7603	6,853	0.2335	< 0.0001	5.542	3.125	9.828
S1	PN	6,848	0.146	6,854	0.1459	0.0878	3.793	0.7325	19.643
S1	Asthma	6,845	1.023	6,844	1.096	0.0127	1.518	1.091	2.112
S1	Food allergy	6,849	0.219	6,849	0.146	0.0407	2.263	1.013	5.056
S1	COPD	6,834	1.932	6,835	2.721	0.2354	1.147	0.9145	1.438
S1	VMR/AR	6,844	1.373	6,839	1.682	0.0396	1.336	1.013	1.762
S1	EoE	6,854	0.1459	6,852	0	0.0029	NA	NA	NA
S1	CS	6,852	0.7881	6,841	0.8332	0.0154	1.585	1.089	2.308
S2	AD	2,849	0.9126	2,849	0.351	< 0.0001	5	2.148	11.639
S2	PN	2,849	0.351	2,849	0.351	0.0422	4.483	0.9224	21.785
S2	Asthma	2,849	1.404	2,849	0.9477	0.0008	2.29	1.391	3.768
S2	Food allergy	2,849	0.351	2,849	0.351	0.7596	1.157	0.4548	2.942
S2	COPD	2,849	2.527	2,849	2.843	0.0907	1.321	0.9559	1.827
S2	VMR/AR	2,849	1.86	2,849	1.931	0.0639	1.435	0.9774	2.108
S2	EoE	2,849	0.351	2,849	0	0.0226	NA	NA	NA
S2	CS	2,849	1.088	2,849	1.158	0.124	1.481	0.8953	2.45
S3	AD	1,802	0.5549	1,802	0.5549	0.03	4.901	1.006	23.885
S3	PN	1,802	0.5549	1,802	0.5549	0.3501	0.356	0.037	3.426
S3	Asthma	1,802	0.5549	1,802	1.332	0.0366	0.4483	0.2072	0.9703
S3	Food allergy	1,802	0.5549	1,802	0.5549	0.1347	0.2236	0.0261	1.919
S3	COPD	1,802	1.276	1,802	3.607	0.001	0.455	0.2815	0.7354
S3	VMR/AR	1,802	0.6659	1,802	2.83	< 0.0001	0.2863	0.1521	0.539
S3	EoE	1,802	0.5549	1,802	0.5549	0.3131	3.325	0.2848	38.831
S3	CS	1,802	0.5549	1,802	1.554	0.005	0.3212	0.1394	0.7401
S4	AD	7,955	0.943	7,955	0.214	< 0.0001	6.378	3.715	10.951
S4	PN	NA	NA	NA	NA	NA	NA	NA	NA
S4	Asthma	7,955	1.219	7,955	1.069	0.0008	1.653	1.229	2.223
S4	Food allergy	7,955	0.377	7,955	0.176	0.0001	3.37	1.76	6.452
S4	COPD	7,955	2.439	7,955	1.622	< 0.0001	2.065	1.649	2.587
S4	VMR/AR	7,955	1.483	7,955	1.571	0.0119	1.386	1.073	1.789
S4	EoE	NA	NA	NA	NA	NA	NA	NA	NA
S4	CS	7,955	0.855	7,955	0.754	0.0022	1.729	1.213	2.466
